# Crystal Surface Reactivity of Esterase@Zeolitic Imidazolate Framework Biocomposites

**DOI:** 10.1021/jacs.5c18572

**Published:** 2026-01-02

**Authors:** Emilio Borrego-Marin, Marta E. López-Viseras, Javier D. Martín-Romera, Rebecca Vismara, Francesco Carraro, Paolo Falcaro, Elisa Barea, Jorge A. R. Navarro

**Affiliations:** † Departamento de Química Inorgánica, 16741 Universidad de Granada, Av. Fuentenueva, S/N, 18071 Granada, Spain; ‡ Institute of Physical and Theoretical Chemistry, TU Graz, A-8010 Graz, Austria

## Abstract

Esterase@zeolitic imidazolate framework (Esterase@ZIF) biocomposites have been synthesized by biomineralization of pig liver esterase to explore the synergistic interplay between esterase and the ZIF framework on crystal surface reactivity and biocompatibility. The targeted Esterase@ZIF crystal phases cover a wide range of ZIF topologies, namely, Zn­(mIm)_2_ (ZIF-8, 3D sodalite, microporous, mImH = 2-methylimidazole), Zn­(mIm)_2_·(mImH)_0.5_ (ZIF-L, 2D layered, nonporous), and Zn­(mIm)­(CO_3_)_0.5_ (ZIF-C, 3D, nonporous). As model reactions, we have assessed the hydrolytic decontamination of the G-type nerve agent simulant diisopropylfluorophosphate (DIFP) and esterase activity toward indoxyl acetate hydrolysis. The results show a clear synergistic interplay between esterase and the ZIF framework, which is attributed to the stabilized open-lid conformation of esterase and the reactivity of the ZIF crystal surface, leading to enhanced hydrolytic activity. P–F bond hydrolysis also induces Esterase@ZIF crystal surface degradation releasing mImH and Zn^2+^ ions. Released imidazole moieties enable a nucleophilic attack to DIFP-inhibited acetylcholinesterase (DIFP@AChE), promoting AChE reactivation and thereby reversing organophosphorous poisoning. In vitro cytotoxicity assays toward human neuroblastoma cell lines are indicative of increased biocompatibility of Esterase@ZIF in comparison to pristine ZIF materials. These results also exemplify that biomineralization can be used not only to protect enzymes from harsh environments but to modulate crystal surface reactivity and biocompatibility.

## Graphical abstract

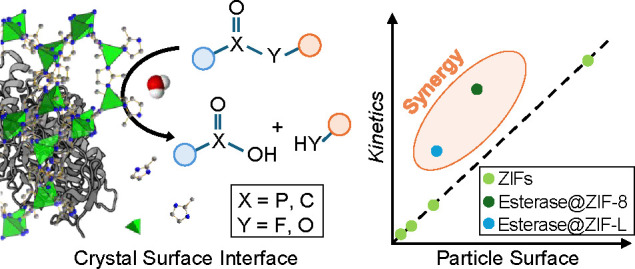

## Introduction

Metal–organic frameworks (MOFs) constitute a versatile class of porous crystalline materials, assembled from metal ions and organic linkers into three-dimensional architectures of exceptional chemical and structural diversity.^1,2^ This intrinsic versatility has endowed them with a wide range of applications, including gas capture/separation,^3–6^ catalysis,^7–10^ and drug delivery.^11–13^ More recently, alongside these established applications, increasing attention has also been directed toward exploring MOFs as matrices for biologically relevant biomacromolecules owing to their modular synthesis, high porosity, and tunable pore chemistries.^14^ The generation of MOF-based biocomposites through the encapsulation of proteins,^15,16^ nucleic acids,^17,18^ or cells,^19–21^ not only extends the functional landscape of reticular materials but also offers new opportunities for addressing challenges in biocatalysis,^22^ biosensing,^23^ delivery of therapeutics,^24^ and biotechnology. MOF–enzyme biocomposites have emerged as particularly compelling systems. Enzymes are highly efficient and selective biocatalysts, yet their inherent fragility, manifested in limited stability under nonphysiological conditions, often restricts their practical deployment.^25^ Encapsulation within MOFs offers a powerful strategy to overcome these limitations by providing a protective microenvironment that can prevent denaturation^26,27^ and in some cases even enhance catalytic performance.^28,29^ Such confinement effects highlight the potential of designed MOF–enzyme hybrid materials to act not only as protective supports but also as active participants that confer new functions to the composite, expanding its utility beyond that of the pristine MOF (synergistic effect).

Among the different classes of MOFs, zeolitic imidazolate frameworks (ZIFs) have emerged as particularly attractive candidates for the preparation of biocomposites.^30–32^ Their ability to form under mild,^33^ water-compatible conditions makes them especially suitable for the encapsulation and stabilization of sensitive enzymes. Biomineralization offers a simple, one-step route to these biocomposites, wherein the biomolecule itself templates MOF nucleation and growth, preserving structure and activity while enabling control over particle size, morphology, and crystallinity.^34^ Moreover, the diversity of available ZIF topologies, ranging from microporous three-dimensional structures to layered or dense polymorphs,^35^ offers a unique platform to investigate how framework architecture influences biomolecule incorporation and functional performance.

Previous work from our research group has shown that the reactivity of ZIFs toward organophosphorous nerve agents is strongly governed by the nature of the exposed crystal surface.^36,37^ Indeed, organophosphorus decontamination triggers ZIF crystal structural degradation as evidenced by the release of imidazole linkers. This aspect is of great importance considering the ability of imidazole-type nucleophilic ligands to reactivate the activity of organophosphorous-inhibited esterase-type enzymes.^36,37^ In this work, we have envisioned that biomineralization of functional biomolecules in ZIFs might be used not only to protect enzymes from harsh environments but also to modulate crystal surface reactivity and biocompatibility. In order to demonstrate this hypothesis, we have targeted three different esterase@zeolitic imidazolate framework (Esterase@ZIF) biocomposites covering a wide range of ZIF topologies, namely, Zn­(mIm)_2_ (ZIF-8, 3D sodalite, microporous, mImH = 2-methylimidazole),^38^ Zn­(mIm)_2_·(mImH)_0.5_ (ZIF-L, 2D layered, nonporous),^39^ and Zn­(mIm)­(CO_3_)_0.5_ (ZIF-C, 3D, nonporous)^40^ following a biomineralization process (Scheme 1). Crystallization of these architectures around the esterase enzyme enabled us to explore the synergy between esterase stabilization and ZIF crystal surface reactivity toward detoxification of the G-type nerve agent model compound diisopropylfluorophosphate (DIFP) as well as esterase activity toward indoxyl acetate hydrolysis (Scheme 1b, c).

**Scheme 1:**
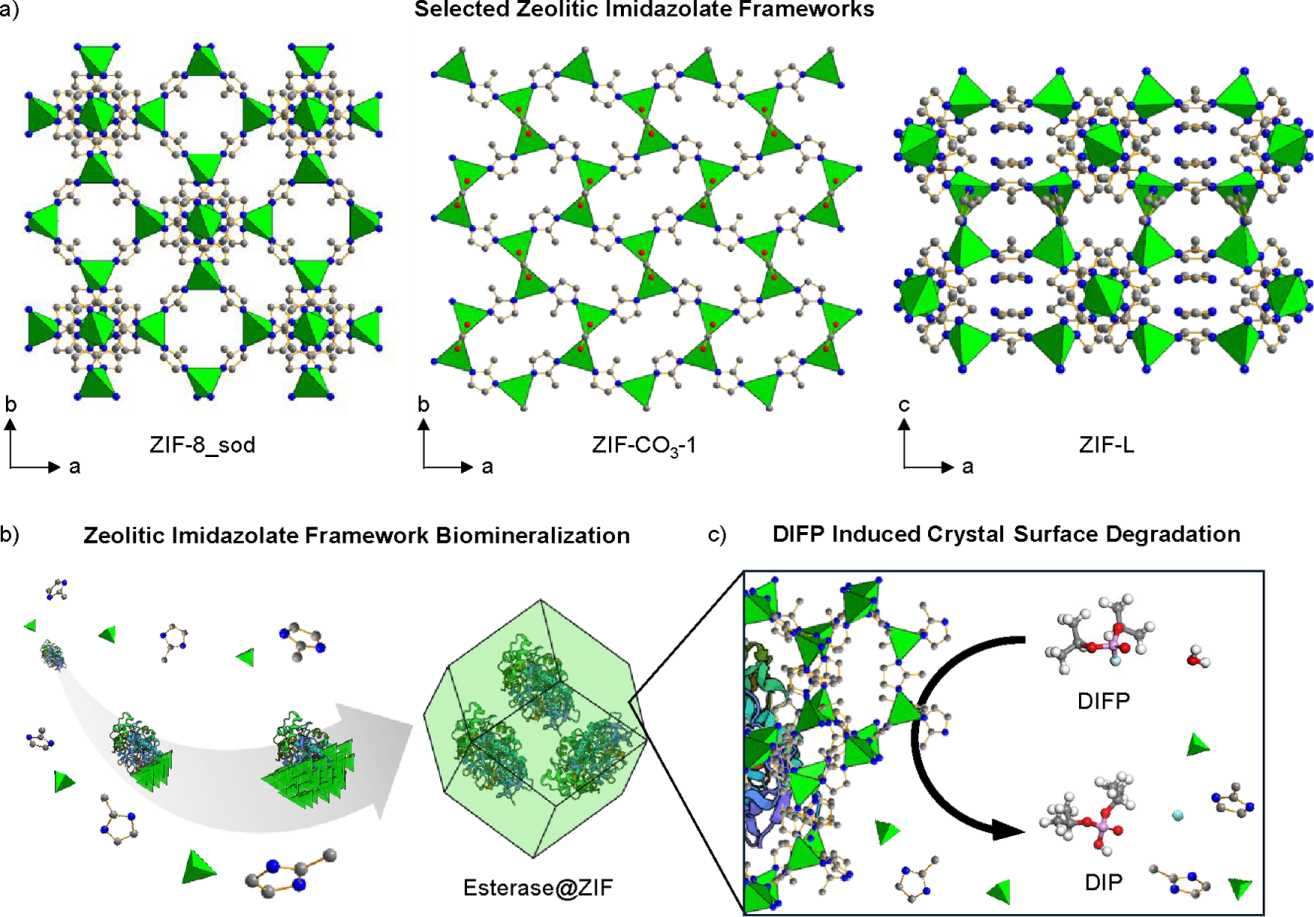
(a) Zeolitic Imidazolate Framework Crystal Structures Studied in This Work: ZIF-8_sod, ZIF-CO_3_-1, and ZIF-L. (b) Biomineralization Process of Zeolitic Imidazolate Frameworks (ZIFs) Around Esterase from 2-Methylimidazole (mImH) and Zn^2+^ Precursors. (c) Diisopropylfluorophosphate (DIFP) Hydrolysis to Nontoxic Diisopropylphosphate (DIP) with Concomitant Crystal Surface Degradation^a^

a Color code: C (gray), F (light blue), H (white), N (blue), O (red), and P (magenta). Zn ions have been represented as green tetrahedra.

## Results and Discussion

### Biocomposite Synthesis and Characterization

We have targeted the formation of three different Esterase@ZIF biocomposites: Esterase@ZIF-8, Esterase@ZIF-C, and Esterase@ZIF-L. We have followed the optimized procedure reported elsewhere for the synthesis of protein@ZIF biocomposites^34^ showing improved esterase loadings compared to literature reports (Table S9). Esterase biomineralization is highly favored under basic conditions of mImH aqueous solutions (pH = 11) due to the negative Z-potential of esterase (−20 mV; isoelectric point, pI = 5.0),^41,42^ which allows the anchoring of Zn^2+^ ions at its surface.^43,44^ Such Zn^2+^–esterase interaction facilitates ZIF biomineralization^45^ around esterase to yield Esterase@ZIF-8_120 nm, Esterase@ZIF-C, and Esterase@ZIF-L biocomposites (Figure 1, see the Supporting Information, Tables S1–S3). The biomineralization mechanism is confirmed by turbidometry assays showing the instantaneous formation of a cloudy suspension upon the addition of the metal salt to an aqueous solution of imidazole and esterase. A similar experiment in the absence of esterase leads to a clear solution (see Figures S1–S6, Movie S1). Further, esterase adsorbed on ZIF crystals leads to Esterase-on-ZIF materials with a significant drop (1 order of magnitude) in biomolecule loading compared to biomineralized materials, as gathered in Table S8.

**Figure 1:**
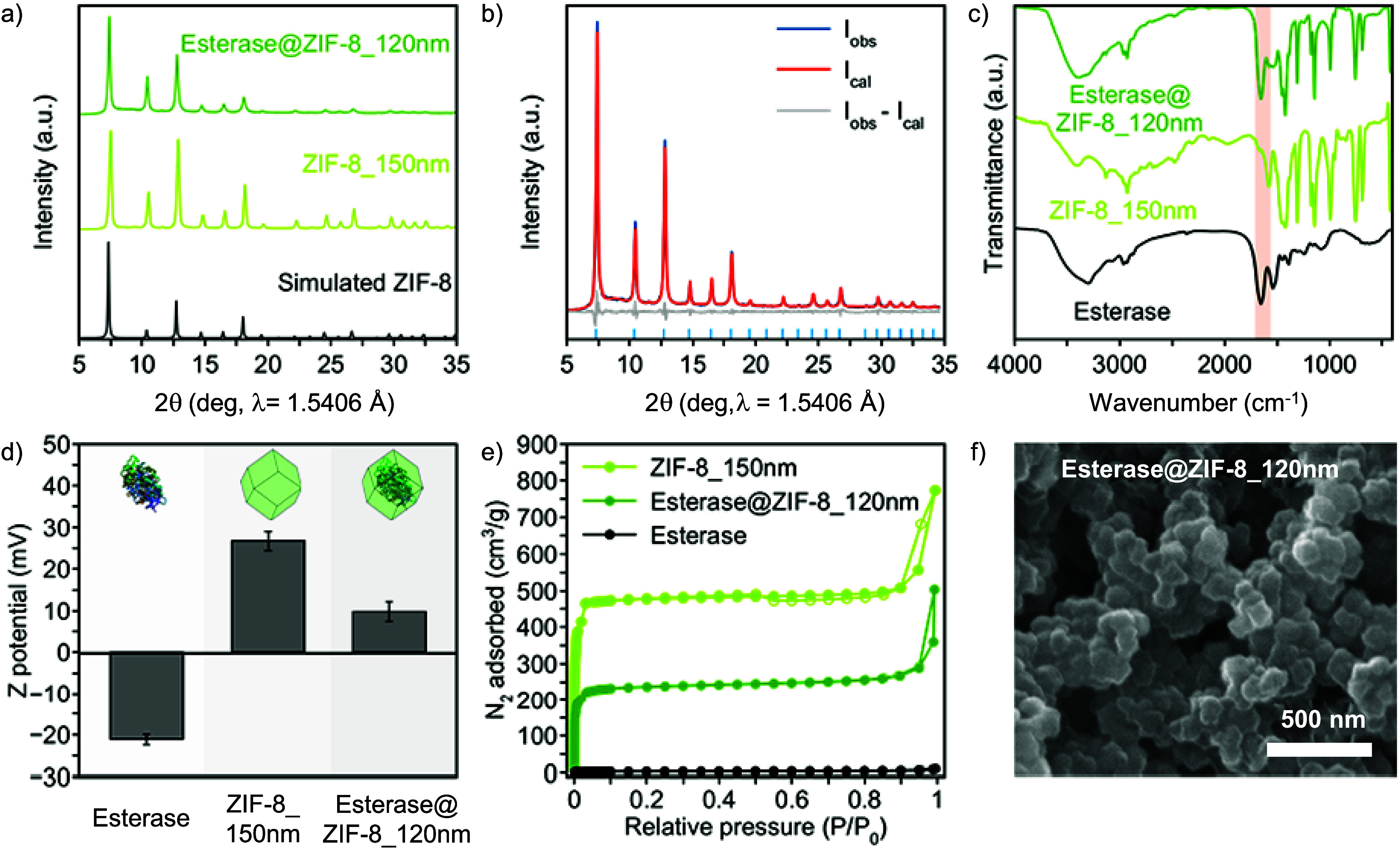
Esterase@ZIF-8_120 nm biomineralization process: (a) Powder X-ray diffraction patterns of ZIF-8_150 nm (experimental, light green; calculated, black) and Esterase@ZIF-8_120 nm biocomposites (experimental, dark green). (b) Graphical result of the whole powder pattern refinement carried out with the Le Bail method (*R*_p_ = 0.05738; *R*_wp_ = 0.07395) on the PXRD pattern of Esterase@ZIF-8_120 nm in terms of observed, calculated, and difference traces (blue, red, and gray, respectively). The positions of the Bragg reflections are indicated by blue ticks. (c) Fourier transform infrared spectra (FTIR) of Esterase (black), ZIF-8_150 nm (light green), and Esterase@ZIF-8_120 nm (dark green) materials. Amide I band (1700–1610 cm^–1^) of the peptide backbone of esterase highlighted in light orange. (d) Z-potential values of esterase in mImH solution (left), pristine ZIF-8_150 nm (middle), and Esterase@ZIF-8_120 nm biocomposites (right). (e) Nitrogen adsorption isotherms at 77 K of ZIF-8_150 nm, Esterase@ZIF-8_120 nm, and esterase materials. The adsorption and desorption process of each N_2_ isotherm is represented by filled and empty dots, respectively. (f) Scanning electron microscopy image of Esterase@ZIF-8_120 nm biocomposites.

Crystal phase purity and location and quantification of esterase in the biocomposite crystals were ascertained from different analytical tools. Powder X-ray diffraction (PXRD) of synthesized biocomposites confirmed the biomineralization in their crystal phase pure form (Figures 1a, b, and S7–S9). The presence of esterase in the biomineralized crystals was first demonstrated by Fourier transform infrared (FTIR) spectroscopy with the appearance of the amide I band of esterase in the range of 1700–1610 cm^–1^ (Figures 1c and S10–S12). The amount of enzyme encapsulated was determined using the Bradford assay (Table S8) and thermogravimetric analysis (TGA) (Figures S13–S15, Tables S12–S14). Overall, all three biomineralization processes achieved good esterase encapsulation efficiencies of 75% (i.e., protein loading capacity (LC) ∼ 0.37 mg_esterase_/mg_biocomposite_) for Esterase@ZIF-8_120 nm and Esterase@ZIF-L and 96% (LC ∼ 0.47 mg_esterase_/mg_biocomposite_) for Esterase@ZIF-C, as expected for a low pI protein (see Tables S8 and S9).^46^ Biocomposite crystals possess a significantly lower positive Z-potential than pristine ZIFs agreeing with the probable coexistence of Lewis acidic Zn^2+^ ions and esterase biomolecules close to their crystal surface (Figures 1d and S16). Indeed, X-ray photoelectron spectroscopy (XPS) analysis, with a 10 nm depth, shows a high S content (0.50–0.70 wt %) and a significant 2 to 3 fold depletion of Zn content in biocomposites with respect to pristine ZIFs and esterase/ZIF physical mixtures. These data agree with a significant amount of biomolecules near the crystal surface, which we estimate to increase from an average of 40% to 80–90% range (Figures S17–S19, Table S15). N_2_ adsorption isotherms at 77 K for Esterase@ZIF-8_120 nm are indicative of a 50% diminution of adsorption capacity related to the nonporous nature of esterase (Figure 1e). However, for nonporous Esterase@ZIF-C and Esterase@ZIF-L the respective BET values of 120 and 45 m^2^/g are significantly higher than the expected few square meters per gram for pristine nonporous ZIF crystal phases (Figures S20–S22, Table S16). The observed residual porosity is a probable consequence of the formation of residual interparticle macroporosity related to the smaller particle size of biocomposites and to a higher concentration of defects related to the heterogeneous nucleation of the biomineralization process.^34^ Scanning and transmission electron microscopy (SEM/TEM) were used to determine the shape and size of the biocomposites. SEM/TEM images show 120 ± 20 nm truncated cubic crystals for Esterase@ZIF-8_120 nm, 560 ± 80 nm leaf-shaped crystals for Esterase@ZIF-L and undefined aggregates for Esterase@ZIF-C (Figures 1f and S23–S25). The obtained particle sizes are two–ten times smaller than the typical synthesis of pristine ZIF frameworks, confirming the seeding effect of the protein during ZIF formation. Electron microscopy–energy dispersive X-ray spectroscopy (TEM/SEM-EDX) for Esterase@ZIF biocomposites exhibits highly homogeneously distributed S and P elements (absent in ZIFs) associated with the enzyme and indicative of the main role of the biomolecules in the biomineralization process (Figures S26–S30). For comparative purposes, we have also prepared analogue bovine serum albumin (BSA) biocomposites (lacking esterase function), employing similar synthetic conditions, leading to BSA@ZIF-8_120 nm, BSA@ZIF-8_1.2 μm, and BSA@ZIF-C hybrids (see the Supporting Information, Tables S4, S5).^34^ Crystal phase purity and BSA encapsulation were confirmed by PXRD, FTIR, N_2_ adsorption isotherms, Z-potential, SEM, and Bradford experiments (Figures S31–S40, Tables S8, S17).

### Synergistic Interplay of Esterase and ZIF on Biocomposite Function

Once we confirmed the successful synthesis of the Esterase@ZIF biocomposites, we proceeded to evaluate the possible synergistic interplay between esterase and the ZIF framework. With this aim, we have assessed the hydrolytic decontamination of the G-type nerve agent simulant diisopropylfluorophosphate (DIFP) into nontoxic diisopropylphosphate (DIP), as the model reaction (Figure 2a), as well as esterase activity toward indoxyl acetate hydrolysis. Esterase@ZIF biomaterials’ (0.084 mmol of ZIF) reactivity toward DIFP (0.029 M, 0.5 mL), under simulated biological conditions (0.1 M Tris-HCl aqueous buffer solution, pH = 7.4), was compared to their individual components, free esterase and pristine ZIF-8_150 nm, ZIF-L, and ZIF-C crystal phases (Figures 2b and S41–S43). Additionally, we have also evaluated the behavior of BSA@ZIF-8 (120 nm–1.2 μm) and BSA@ZIF-C as close models of Esterase@ZIF biocomposites lacking esterase activity. The results indicate a clear improvement in P–F bond hydrolysis for the Esterase@ZIF systems in comparison to their individual components and BSA@ZIF biocomposites (Figures 2a, b, and S44–S46). Indeed, free esterase is only able to degrade 37% of DIFP after 24 h (half-life time (*t*_1/2_) of 2,490 min). ZIF-8_150 nm and BSA@ZIF-8_120 nm exhibit moderate DIFP degradation *t*_1/2_ of 40 and 22 min, respectively (Figure 2b, Table S10). By contrast, the Esterase@ZIF-8_120 nm biocomposite exhibits a *t*_1/2_ of 8 min (Figure 2b, Table S10). Downsizing Esterase@ZIF-8 particles to 60 nm leads to a further shortening of *t*_1/2_ to 2.1 min (Figures S47–S50, Table S10). Precise control over particle size in batch syntheses of protein@ZIF biocomposites remains challenging; in this work, we therefore focus on particles of similar sizes (ca. 120–150 nm) to provide meaningful controls with matched dimensions across Esterase@ZIF-8_120 nm, ZIF-8_150 nm, and BSA@ZIF-8_120 nm. Similarly, Esterase@ZIF-C (*t*_1/2_ of 7.2 min) and Esterase@ZIF-L (*t*_1/2_ of 30 min) biocomposites exhibit one to 2 orders of magnitude lower half-life times than their individual components (Figures S41, S42, Table S10). Additionally, physical mixtures of ZIFs and the free esterase enzyme give rise to much lower reactivity (*t*_1/2_ of 54–700 min) than Esterase@ZIF biocomposites (Figures 2c and S51–S53). These results agree with the synergistic interplay of esterase and the host ZIF framework in the biocomposites.

**Figure 2:**
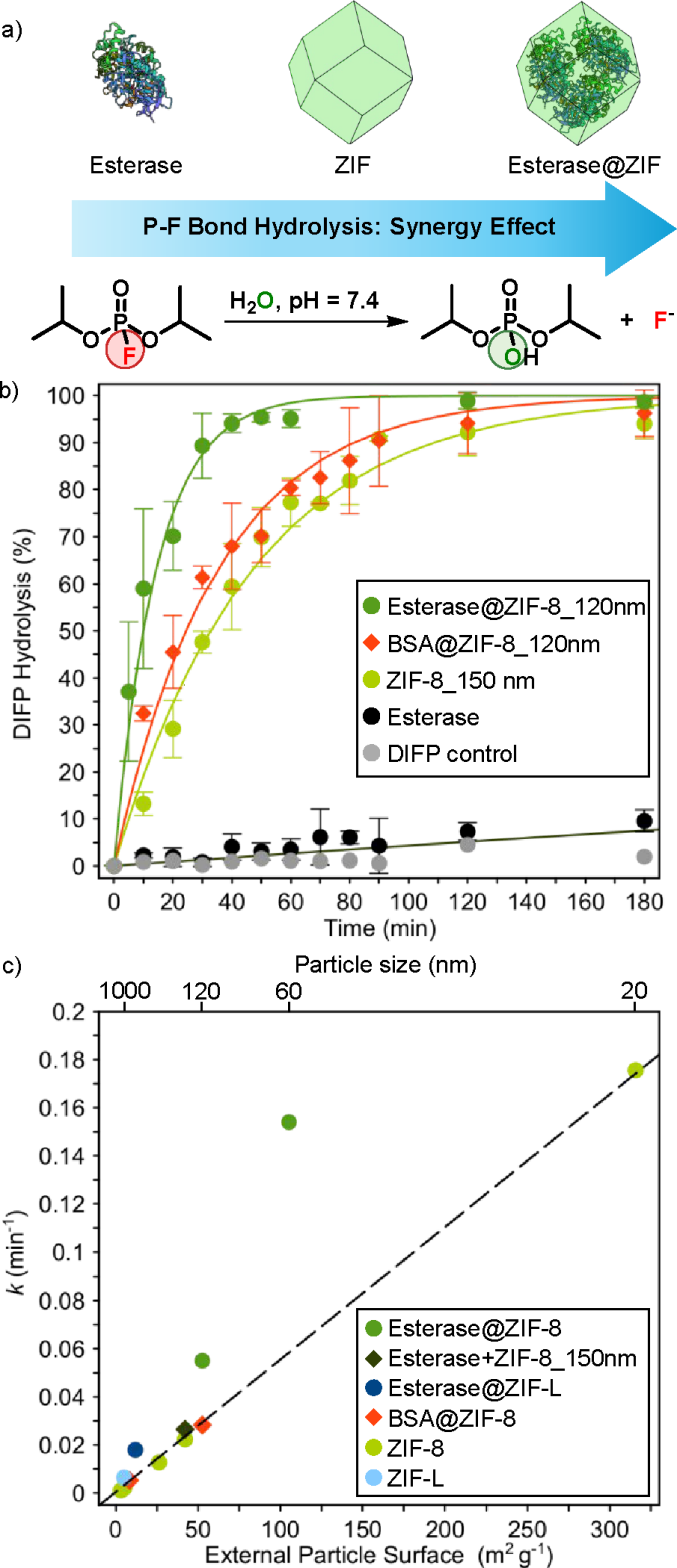
Synergistic impact of esterase biomineralization on crystal surface reactivity toward the diisopropylfluorophosphate (DIFP) nerve agent model compound: (a) Schematic of the P–F bond hydrolytic breakdown of the G-type simulant DIFP by Esterase@ZIF biocomposites. (b) Comparative profiles for DIFP hydrolytic degradation by esterase, ZIF-8_150 nm, BSA@ZIF-8_120 nm, and Esterase@ZIF-8_120 nm biomaterials in simulated biological conditions. (c) Correlation between the kinetics constant for DIFP degradation and the external crystal surface area for ZIF-8 particles (20 nm–2 μm), Esterase@ZIF-8 (60–120 nm), the esterase+ZIF-8_150 nm physical mixture, and Esterase@ZIF-L and BSA@ZIF-8 (120 nm–1.2 μm) biocomposites. The dotted line represents the tendency line for ZIF-8 particles (20 nm–2 μm). Experimental conditions: DIFP (0.029 M), 0.084 mmol of ZIF (for free esterase, we considered the amount of esterase biomineralized in the Esterase@ZIF materials), DMF (0.029 M, internal reference), and Tris-HCl (0.1 M, pH = 7.4, 0.5 mL) at room temperature.

Hot filtration tests show the halting of P–F bond hydrolysis, which agrees with the heterogeneity of the decontamination process (Figure S54). This is further confirmed by control reactions in the homogeneous phase with Zn­(OAc)_2_ and mImH precursors in 0.1 M Tris-HCl buffer (pH = 7.4) showing no detectable DIFP hydrolysis (Figure S55). In our previous work, we found a linear relationship between the kinetic constant for DIFP hydrolysis and the exposed external surface area of different ZIF-8 micro- and nanoparticles.^36^ Noteworthy, the kinetic constants for the two Esterase@ZIF-8 biocomposites (particle sizes 60 and 120 nm) and Esterase@ZIF-L (560 nm leaves) are located well above the expected linear trend. Remarkably, BSA@ZIF-8 (120 nm–1.2 μm) biocomposites, lacking esterase functionality, follow the expected linear trend (Figure 2c). These results confirm a synergy between the biocomposite components and that hydrolytic degradation of DIFP is taking place at the Esterase@ZIF crystal surface. By contrast, organophosphorus acid anhydrolase@Zr-MOF hybrids based on physisorbed enzymes on mesoporous Zr-MOFs show high DIFP hydrolysis at the enzyme site, with the main role of the MOF is to provide a protective environment that stabilizes the enzyme from harsh environments (Table S11).^47,48^

We have also evaluated the esterase activity of biocomposites toward indoxyl acetate hydrolysis. The results indicate that all three biomineralized crystalline phases retain significant esterase activity, namely, 38, 89, and 92%, for Esterase@ZIF-8_120 nm, Esterase@ZIF-L, and Esterase@ZIF-C biocomposites, respectively (see the Supporting Information, Figures S56, S57). By contrast, pristine ZIF crystalline materials do not show any esterase activity (Figure S57). The high esterase activity of Esterase@ZIF-L and Esterase@ZIF-C nonporous phases and the bulk of the substrate further point toward the exposed location of esterase near the crystal surface, in agreement with Z-potential and XPS analyses (see above). Additionally, we have also evaluated Esterase@ZIFs’ ability to preserve their enzymatic activity under inhospitable environments, such as elevated temperatures (60–90 °C). Enzymatic activity for free esterase significantly decreases above 70 °C, becoming nearly undetectable after 1 h of incubation at 80 °C (Figure S58). By contrast, Esterase@ZIF biocomposites can retain 50–70% of esterase activity across the entire temperature range (Figure S58). The preservation of esterase activity under extreme conditions can be related to a conformational stabilization of an open-lid state conformation taking place along the mineralization process, in agreement with literature reports for improved enzymatic activity of lipases^49–53^ and esterases^54^ immobilized on hydrophobic surfaces. Indeed, FTIR studies indicate that the α-helix content of free esterase decreases upon biomineralization, consistent with open-lid conformation, from 32% to 18.7%, 19.4%, and 23.6% on Esterase@ZIF-8_120 nm, Esterase@ZIF-C, and Esterase@ZIF-L, respectively (Figure S59, Tables S18, and S19). Furthermore, PXRD measurements of the Esterase@ZIF biocomposites reveal that the crystallinity of the various ZIFs remains unaltered across all tested temperature ranges (Figure S60). This suggests that the enhanced stability observed in these biomaterials is bidirectional, leading to stabilization of both enzyme conformation and ZIF crystal phase. Indeed, while pristine ZIF-L undergoes a crystal phase transition to the more stable sodalite-type ZIF-8 in the presence of ethanol at 60 °C after 72 h of incubation,^55^ the Esterase@ZIF-L biocomposite retains its crystal phase (Figure S61). In our biocomposites, we hypothesize that the synergistic interplay between the guest esterase and the host ZIF-8 matrix depends on three main components: the active crystal phase, stabilization of the active enzyme conformation, and the creation of surface defects (residual porosity) typically induced by heterogeneous nucleation events.^56^

### DIFP-Induced Crystal Surface Degradation, Esterase Reactivation, and Biocompatibility

A final aspect that we have studied is the coupled DIFP-induced crystal surface structural degradation of biocomposites and reactivation of organophosphorus-inhibited esterase by released nucleophilic imidazolates (Figures 3 and 4). At this point, it is important to highlight that acetylcholinesterase (AChE), in the neuronal system, is the biological target of nerve agents leading to neurotransmission disruption with paramount negative consequences.^57^

**Figure 3:**
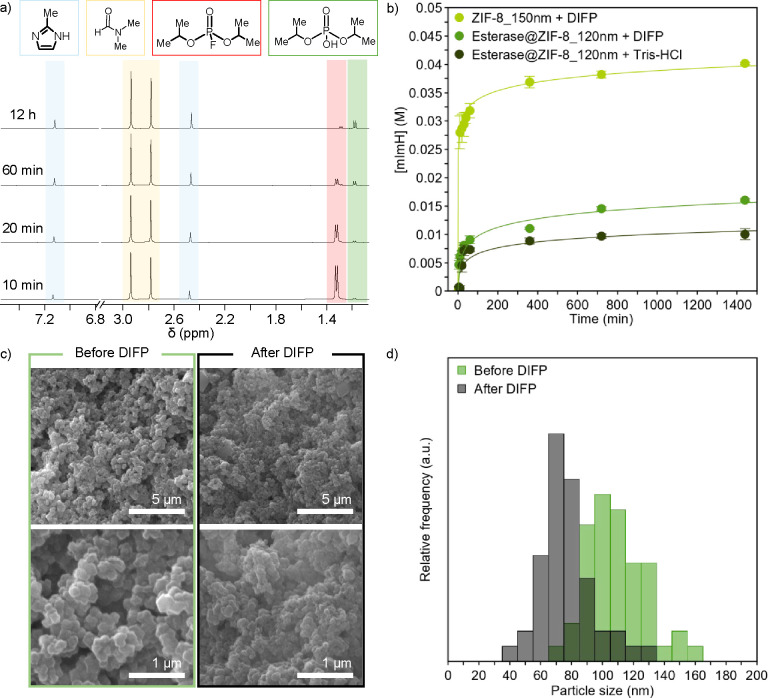
DIFP-induced crystal surface structural degradation: (a) ^1^H NMR follow-up of Esterase@ZIF-8_120 nm degradation upon exposure to DIFP (0.029 M) in Tris-DCl (0.1 M, pD = 7.8) showing the release of mImH and DIFP hydrolysis. (b) Profiles of mImH ligand release from ZIF-8_150 nm and the Esterase@ZIF-8_120 nm crystal surface after incubation with DIFP (0.029 M) in Tris-HCl (0.1 M, pH = 7.4) buffer solution. (c) Scanning electron microscopy (SEM) images of the Esterase@ZIF-8_120 nm biocomposite before and after exposition to DIFP (0.029 M) in Tris-HCl (0.1 M, pH = 7.4) at different magnifications. (d) Particle size distribution from SEM images of Esterase@ZIF-8_120 nm crystals before (green bars) and after (black bars) incubation with DIFP (0.029 M) in Tris-HCl (0.1 M, pH = 7.4).

**Figure 4:**
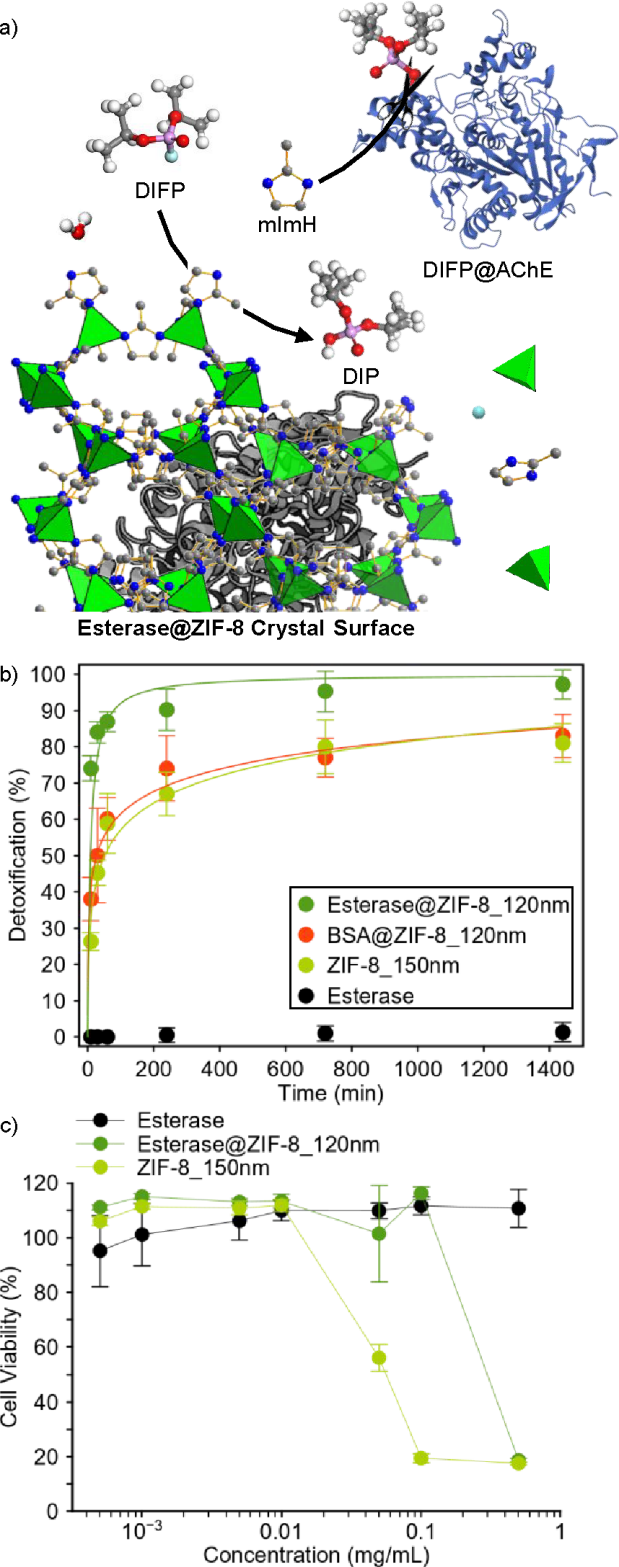
Detoxification of acetylcholinesterase and biocompatibility: (a) Schematic description of the organophosphorus detoxification process by the Esterase@ZIF-8 biocomposite consisting of (i) DIFP hydrolysis to nontoxic diisopropylphosphate (DIP), (ii) ZIF crystal surface-induced degradation releasing mImH molecules, and (iii) mImH nucleophilic reactivation of DIFP-inhibited acetylcholinesterase (DIFP@AChE). (b) Detoxification profile (AChE reactivation) by free esterase, ZIF-8_150 nm, BSA@ZIF-8_120 nm, and Esterase@ZIF-8_120 nm biocomposites in Tris–HCl buffer solution (0.1 M, pH = 7.4). (c) Cell culture viability assays of human neuroblastoma SH-SY5Y cells upon exposure to the esterase@ZIF-8_120 nm biocomposite and its individual components esterase and ZIF-8_150 nm after 24 h of exposure. Color code: C (gray), F (light blue), H (white), N (blue), O (red), and P (magenta). Zn^2+^ ions are represented as green tetrahedra.

^1^H NMR measurements of supernatants of Esterase@ZIF biocomposites exposed to a DIFP (0.029 M) solution in simulated biological conditions (0.1 M Tris-DCl buffer, pD = 7.8) show the simultaneous conversion of DIFP into nontoxic subproduct diisopropylphosphate (DIP) and the release of 2-methylimidazole, indicative of ZIF crystal surface degradation (Figures 3a, b, S62–S66 and Table S20). DIFP degradation by Esterase@ZIF-8_120 nm shows a lower-than-expected DIP signal, in the supernatant solution, indicative of DIP adsorption into biocomposite pores, during the decontamination reaction, as evidenced from ATR-FTIR 1000–1100 cm^–1^ broad absorption band characteristics of P–O stretches (see Figure S66). The slower DIFP hydrolysis rate in the NMR experiments compared to GC experiments (see above) is a consequence of the lack of stirring in the NMR tube and the slower hydrolysis in D_2_O compared to H_2_O.^58^ Notably, the imidazole release profile is slower than that of pristine ZIFs and is a further indication of the selective modulation of crystal surface reactivity of biocomposites (Figures 3b and S67). SEM images of the Esterase@ZIF-8_120 nm biocomposite before and after exposition to DIFP degradation (0.029 M, 0.5 mL, 0.1 M Tris-HCl, pH = 7.4) show a particle size reduction from 120 to 80 nm (Figure 3c, d). Full profile Le Bail fitting of powder X-ray diffraction (PXRD) patterns of Esterase@ZIF-8_120 nm before and after DIFP treatment is also indicative of crystal size domain diminution from 82 to 54 nm (Figures S68 and S69, Table S21). Similarly, smaller Esterase@ZIF-8_60 nm particles diminish to 40 nm (Figure S70) and the crystal size domain is reduced from 27 to 18 nm (Figures S71–S73, Table S22) as evidenced by electron microscopy and PXRD, respectively. Esterase@ZIF-L exhibits a similar behavior (Figures S65, S67, and S74–S76). By contrast, Esterase@ZIF-C exposure to DIFP leads to a phase shift to the sodalite ZIF-8 crystal phase (Figures S76–S78).

Remarkably, despite structural degradation of the ZIF crystal surface, no release of encapsulated esterase was observed for any of the Esterase@ZIF biocomposites (Bradford test, detection limit 100 μg/mL), which is related to the enhanced crystal phase stability of biocomposites under simulated biological conditions (0.1 M Tris-HCl, pH = 7.4) (Figure S79). By contrast, literature reports show that acidic conditions (e.g., pH ∼ 5.5) can trigger the quantitative release of proteins from protein@ZIF biocomposites.^59^

After determining the biocomposite crystal surface reactivity, we have studied the esterase activity inhibition by DIFP on pig liver esterase (esterase) and human AChE and the subsequent reactivation of AChE by released imidazole.

First, we demonstrated DIFP inhibition of the pig esterase enzyme across a range of concentrations from 10^–8^ to 10^–2^ M in simulated biological conditions (0.1 M Tris-HCl buffer solution, pH = 7.4) (Figure S80). IC_50_ and IC_90_ were approximately 5 × 10^–7^ and 10^–4^ M, respectively. Subsequently, we compared the retained esterase enzymatic activity of Esterase@ZIF biocomposites after incubation at the inhibitory concentrations IC_50_ and IC_90_ for 1 h at 37 °C (Figure S81). Noteworthy, at [DIFP] of 10^–4^ M (IC_90_), the retained enzymatic activity for Esterase@ZIF biocomposites is 5 to 8 fold higher than that for free esterase. A control experiment for a physical mixture of ZIFs and esterase reveals negligible differences with respect to the free enzyme (Figure S81). This observation reinforces the notion that immobilization of esterase within Esterase@ZIF hybrids provides protective effects for this enzyme activity.

A second experiment focused on assessing the impact of imidazole release, from biocomposite crystal surface degradation, on the reactivation of 50% inhibited AChE ([DIFP] of 5 × 10^–5^ M) (Figure S82). Such AChE reactivation can be taken as a proof-of-concept model for organophosphorus detoxification (Figure 4a). Previous studies of our group showed that imidazole release during the structural degradation of the ZIF crystal surface leads to reactivation of organophosphorous-inhibited AChE.^36^ Now, we have explored the advantage of employing Esterase@ZIF hybrid systems over their isolated components, Esterase and ZIFs. First, we examined the ability of mImH molecules to reactivate 50% inhibited AChE under simulated biological conditions (0.1 M Tris-HCl buffer solution, pH 7.4) (Figure S82). Subsequently, we demonstrated the dual functionality of Esterase@ZIF systems for DIFP simulant nerve agent hydrolysis (0.029 M, 0.5 mL), followed by the reactivation of AChE under simulated biological conditions (0.1 M Tris-HCl buffer solution, pH = 7.4). Noteworthy, Esterase@ZIF systems are able to reactivate DIFP-inhibited AChE with faster kinetics and to a greater extent than their individual components (Figures 4b and S83). Indeed, AChE detoxification half-life times follow the trend Esterase@ZIF-8_120 nm (*t*_1/2_ = 8.8 min) < BSA@ZIF-8_120 nm (*t*_1/2_ = 27 min) < ZIF-8_150 nm (*t*_1/2_ = 52 min) ≪ Esterase. Finally, we carried out in vitro cytotoxicity assays using human SH-SY5Y neuroblastoma cells to evaluate the biocompatibility of the Esterase@ZIF hybrid systems. The results indicate increased biocompatibility for all Esterase@ZIF materials: the IC_50_ values for Esterase@ZIF-8, Esterase@ZIF-L, and Esterase@ZIF-C fall in the 0.3–0.6 mg·mL^–1^ range, i.e., approximately 1 order of magnitude higher than for the corresponding pristine ZIF materials (IC_50_ = 0.03 mg·mL^–1^) (Figures 4c and S84). The enhanced biocompatibility should be related to the high sensitivity of neuroblastoma cells to Zn,^60^ with the crystal phase stabilization being beneficial, as well as the exposed esterase on the biocomposite crystal surface. The observed DIFP-induced cascade reaction process of DIFP hydrolysis, crystal surface degradation, AChE reactivation, and increased biocompatibility further highlights the synergistic effect arising from the formation of Esterase@ZIF hybrids.

## Conclusions

In this work, we successfully synthesized three different zeolitic imidazolate frameworks around an esterase enzyme using a biomimetic mineralization process, achieving high esterase loadings and good retained enzymatic activity. These Esterase@ZIF biocomposites showed improved performance in nerve agent surrogate hydrolysis in simulated biological conditions in comparison to individual esterase and ZIF components, irrespective of the porous and nonporous nature of the ZIF crystals. Indeed, P–F bond hydrolysis takes place at the Esterase@ZIF crystal surface and is responsible for ZIF crystal surface structural degradation with concomitant release of 2-methylimidazole molecules. The high esterase activity of nonporous phases and the bulk of DIFP and indoxyl acetate substrates point to the exposed location of esterase near the crystal surface. Finally, the released imidazole structural components being able to reactivate organophosphorus-inhibited acetylcholinesterase activity beyond the crystal surface exemplify the organophosphorus-triggered cascade events both on the biocomposite crystal surface and beyond it. Beyond enhancing DIFP hydrolysis and AChE reactivation, esterase biomineralization markedly improves cytocompatibility: for SH-SY5Y neuroblastoma cells, the IC_50_ of Esterase@ZIF-8 increases by approximately 1 order of magnitude relative to pristine ZIF-8 (0.3–0.6 vs 0.03 mg·mL^–1^), indicating substantially reduced Zn-related toxicity under the same conditions.

These results also exemplify that biomineralization might be used not only to protect enzymes from harsh environments but also to modulate crystal surface reactivity and biocompatibility. Future work is ongoing to show if this detoxification approach might be translated to real G-type agents and other organophosphorus compounds where surface-induced conformational changes and controllable structural component release might be exploited for additional applications.

## Supplementary Material





## Notes

Turbidometry assays (MOV)
Synthetic protocols, full characterization of Esterase@ZIF biocomposites, details of DIFP decontamination, esterase and AChE inhibition, reactivation assays and cell viability tests, additional references (PDF)
