# Supplementary material for: Crystal Surface Reactivity of Esterase@Zeolitic Imidazolate Framework Biocomposites
Source: J Am Chem Soc. 2026 Jan 2;148(1):1646–54. doi: 10.1021/jacs.5c18572 (PMC12814183; doi:10.1021/jacs.5c18572)
Supplement: Supplementary file 2 [file ja5c18572_si_002.pdf]

Supporting Information

Crystal surface reactivity of Esterase@Zeolitic Imidazolate Framework Biocomposites

Emilio Borrego-Marin[a], Marta E. López-Viseras[a], Javier D. Martín-Romera[a], Rebecca Vismara[a], Francesco Carraro[b], Paolo Falcaro[b], Elisa Barea[a] and Jorge A. R. Navarro[a]*

[a] Universidad de Granada, Departamento de Química Inorgánica, Av. Fuentenueva, S/N, 18071, Granada, Spain,

[b] Institute of Physical and Theoretical Chemistry, TU Graz, A-8010 Graz, Austria

Email: jarn@ugr.es

Table of Contents

S1. General Methods and Materials 3
S2. Protocols. 4
S2.1. Synthetic procedures 4
S2.1.1. Synthesis of Esterase@ZIF biocomposites. 4
S2.1.2. Synthesis of ZIF-8_150nm 5
S2.1.3. Synthesis of ZIF-L. 5
S2.1.4. Synthesis of ZIF-CO_3-1 5
S2.1.5. Synthesis of BSA@ZIF biocomposites. 5
S2.1.6. Synthesis of Esterase@ZIF-8_60nm 6
S2.1.7. Synthesis of Esterase adsorbed on ZIF 7
S2.2. Evaluation of protein encapsulation efficiency. 7
S2.2.1. Encapsulation efficiency from the supernatant. 7
S2.2.2. Encapsulation efficiency from digested ZIFs. 8
S2.3. Nerve agent simulant degradation 9
S2.3.1. DIFP degradation by ZIFs and Protein@ZIF biocomposites 9
S2.3.2. Esterase+ZIFs physical mixture. 10
S2.3.3. Hot filtration test. 10
S2.3.4. Control experiments. 10
S2.4. Enzymatic Activity. 11
S2.4.1. Determination of Michaelis-Menten constant for AChE and Esterase enzymes. 11
S2.4.2. Inhibition of enzymatic activity by diisopropylfluorophosphate. 11
S2.4.3. Retained enzymatic activity after Esterase biomineralization. 11
S2.4.4. Reactivation of the enzymatic activity by 2-methylimidazole. 12
S2.4.5. Retained enzymatic activity with temperature. 13
S2.4.6. Retained enzymatic activity at Inhibitory Concentrations. 13
S2.4.7. Detoxification experiments 14
S2.5. Cytotoxicity assay 15
S3. Experimental results 16
S3.1. Characterization of Esterase@ZIF biocomposites 16
S3.2. Characterization of BSA@ZIF biocomposites 47
S3.2. Synergistic interplay of esterase and ZIF on biocomposite function 57
S3.3. Esterase enzymatic activity of biocomposites 72
S3.4. DIFP induced ZIF crystal surface degradation 78
S3.4. Detoxification ability of Esterase@ZIF biocomposites 94
S4. References 99

S1. General Methods and Materials

Materials and reagents. All chemical reagents and solvents were commercially obtained from Sigma-Aldrich and were used without further purification.

Powder X-Ray Diffraction (PXRD) analysis. PXRD data were collected on a Bruker D2 PHASER diffractometer equipped with a Cu anode (Kα radiation, λ= 1.5418 Å), collecting with a 1mm slit in the 5-35° 2θ range with steps of 0.02° and a time between each step of 1 s.

Fourier Transformed Infrared (FTIR) analysis. FTIR spectra were recorded on a THERMO NICOLET IR200 equipped with an ATR module.

Nitrogen adsorption isotherms. Nitrogen adsorption isotherms were measured at 77 K on a Micromeritics 3Flex volumetric instrument. Prior to measurement, samples were heated at 393 K for 12 h and outgassed to 10^{-1} Pa in a Micromeritics SmartVacPrep Module.

Thermogravimetric Analysis (TGA). TGA experiments were performed in a METTLER TOLEDO equipment from the Scientific Instrumentation Centre (CIC) of the University of Granada. In a typical analysis, TGA measurements were conducted in an oxidative atmosphere (air). The weight change of the samples was recorded from 30 to 950 °C with a ramp rate of 5 °C min^{-1}.

Scanning Electron Microscopy (SEM) analysis. SEM images were obtained on a JEOL JSM-6490LV Microscope system from the Central Research Support Services of the University of Malaga.

Transmission Electron Microscopy- Energy Dispersive X-Ray Spectrometry (TEM-EDX). TEM-EDX images were obtained in a HAADF Thermo Fisher Scientific TALOS F200X in the Scientific Instrumentation Centre (CIC) of the University of Granada.

Z potential analysis. Z potential data was obtained on a Litesizer DLS 500 Anton-Paar employing Omega Cuvettes made from polycarbonate and filled with 600 µL of sample.

X-ray Photoelectron Spectroscopy (XPS). XPS analyses were obtained using a Kratos Axis Ultra-DLD instrument, located at the X-ray Photoelectron Spectroscopy Unit of the Scientific Instrumentation Center (CIC) at the University of Granada.

Diisopropylfluorophosphate (DIFP) degradation experiments. Simulant nerve agent degradation experiments were followed in an Agilent 8860 Gas Chromatograph. This chromatograph has a 16 port autosampler, a HP-5 column (50 m length, 0.320 mm diameter and 1.05 µm thickness) and a FID detector.

Esterase activity determination. Esterase activity was evaluated by using a colorimetric method which employs indoxyl acetate as a substrate that is converted into indigo blue. The concentration of the enzymatic product was calculated by UV−vis spectroscopy at λ = 620 nm using a Nanoquant model Inifinite M200 Pro with 24-well plate.

^{1}H and ^{31}P Nuclear Magnetic Resonance Spectroscopy (NMR). NMR data was recorded on a 400 MHz BRUKER Nanobay Avance III HD High-Definition spectrometer.

Turbidometry assays and UV-vis absorbance was measured in a Cary 60 UV-Vis Spectrophotometer.

S2. Protocols.

S2.1. Synthetic procedures

S2.1.1. Synthesis of Esterase@ZIF biocomposites.

Esterase@ZIF biocomposites were synthesized by varying the composition of zinc salt, 2-methylimidazole (mImH) and esterase in a fixed volume of water. In this way, we have carried out the biomineralization of three different Zeolitic Imidazolate Framework (ZIF) crystalline phases around a pig liver esterase (EC 3.1.1.1), which are sod ZIF-8, ZIF-CO_3-1 (abbreviated as ZIF-C) and ZIF-L. For this purpose, we prepared the following stock solutions:

- $Zn(OAc)_2{\cdot}2H_2O$ solution (160 mM). 0.3512 g of $Zn(OAc)_2{\cdot}2H_2O$ is dissolved in 10 mL of H_2O milli-Q.
- $Zn(NO_3)_2{\cdot}6H_2O$ solution (100 mM). 0.297 g of $Zn(NO_3)_2{\cdot}6H_2O$ is dissolved in 10 mL of H_2O milli-Q.
- mImH solution-1 (2560 mM). 2.1018 g of 2-methylimidazole is dissolved in 10 mL of H_2O milli-Q.
- mImH solution-2 (1000 mM). 0.821 g of 2-methylimidazole is dissolved in 10 mL of H_2O milli-Q.
- Esterase solution (26.3 mg/ml). 52.6 mg of esterase is dissolved in 2 mL of H_2O milli-Q.

In a typical experiment, the synthesis is carried out in 2 mL Eppendorf tubes (final reaction volume 2 mL), and 6 replicates for each Esterase@ZIF material are made. For each replicate, we prepared a “metal” and a “Ligand+esterase” solution in H_2O milli-Q according to tables S1-2.

Table S1. Volume (μL) of stock solutions to synthesize Esterase@ZIF-8_120nm and Esterase@ZIF-C biocomposites

Sample	Metal Solution		Ligand+esterase solution		
	$Zn(OAc)_2{\cdot}2H_2O$	H_2O	mImH solution-1	H_2O	Esterase
Esterase@ZIF-8_120nm	500	500	500	176	324
Esterase@ZIF-C	910	90	234	442	324

Table S2. Volume (μL) of stock solutions to synthesize Esterase@ZIF-L biocomposite.

Sample	Metal Solution		Ligand+esterase solution		
	$Zn(NO_3)_2{\cdot}6H_2O$	H_2O	mImH solution-2	H_2O	Esterase
Esterase@ZIF-L	500	500	400	276	324

“Metal” solution was added to “Ligand+esterase” solution slowly but steadily and mixed 3 times by inversion. The resulting solutions (Table S3) were then incubated at room temperature for 24 h in static conditions. Then, the samples were centrifuged at 15,000 rpm for 5 min and the first supernatant was collected for protein quantification using Bradford assay (to calculate encapsulation efficiency, see S2.2.1). The samples were washed with 1 mL of Milli-Q water and resuspended by mixing with a spatula and 10 s of vortexing afterwards. The washing was repeated 3 times in total. Finally, the samples were air dried at room temperature for 24 h.

Table S3. Final concentrations of Zinc salt, mImH and esterase in each Esterase@ZIF synthesis.

Sample	Zn (mM)	mImH (mM)	Esterase (mg/mL)
Esterase@ZIF-8_120nm	40	640	4.26
Esterase@ZIF-C	72.8	299	4.26
Esterase@ZIF-L	25	200	4.26

S2.1.2. Synthesis of ZIF-8_150nm

0.47 g of zinc nitrate hexahydrate, $Zn(NO_3)_2{\cdot}6H_2O$ (1.8 mmol), were dissolved in 10 mL of methanol and 10 mL of milli-Q water. Besides, 1.0 g of 2-methylimidazole, mImH (60 mmol), was dissolved in 10 mL of methanol. The two solutions were mixed and stirred for 2 h at room temperature. The resulting solid was recovered by centrifugation (10,000 rpm x 10 min) and washed once with methanol. The final product was dried at 110 ºC overnight.[1]

S2.1.3. Synthesis of ZIF-L.

0.540 g of zinc nitrate hexahydrate, $Zn(NO_3)_2{\cdot}6H_2O$ (1.8 mmol), were dissolved in 40 mL of milli-Q water and 1.3 g of 2-methylimidazole (15.8 mmol) were dissolved in a separate solution of 40 mL of milli-Q water. Both solutions were mixed and stirred for 24 hours at room temperature. The resulting white solid was washed with water (3 x 20 mL) and recovered by centrifugation (4,000 rpm, 10 min). Finally, the sample was air dried at room temperature for 24 h.

S2.1.4. Synthesis of ZIF-CO_3-1.

0.264 g of zinc nitrate hexahydrate, $Zn(NO_3)_2{\cdot}6H_2O$ (0.87 mmol), and 0.074 g of 2-methylimidazole (0.87 mmol) were dissolved in 17.6 mL of a mixture of DMF/H_2O (10:1, v/v). The solution was placed in a 23 mL Teflon-lined autoclave and the reaction was heated at 140 ºC for 24h. The resulting white solid was washed with DMF (3 x 20 mL) and MeOH (3 x 20 mL) and recovered by centrifugation (4,000 rpm, 10 min). Finally, the sample was air dried at room temperature for 24 h.

S2.1.5. Synthesis of BSA@ZIF biocomposites.

Similar to Esterase@ZIF biocomposites (see S2.1.1.), BSA@ZIF biocomposites were synthesize by varying the composition of zinc salt, 2-methylimidazole (mImH) and Bovine Serum Albumin (BSA) in a fixed volume of water. We prepare the following stock solutions:

- $Zn(OAc)_2{\cdot}2H_2O$ solution (160 mM). 0.3512 g of $Zn(OAc)_2{\cdot}2H_2O$ is dissolved in 10 mL of H_2O milli-Q.
- mImH solution-1 (2560 mM). 2.1018 g of 2-methylimidazole is dissolved in 10 mL of H_2O milli-Q.
- BSA solution (20 mg/mL). 100 mg of BSA is dissolved in 5 mL of H_2O milli-Q.

In a typical experiment, the synthesis is carried out in 2 mL Eppendorf tubes (final reaction volume 2 mL), and 6 replicates for each BSA@ZIF material are made. For each replicate, we prepared a “metal” and a “Ligand+BSA” solution in H_2O milli-Q according to table S4.

Table S4. Volume (μL) of stock solutions to synthesize BSA@ZIF-8_1.2μm and BSA@ZIF-C biocomposites

Sample	Metal Solution		Ligand+BSA solution		
	$Zn(OAc)_2·2H_2O$	H_2O	mImH solution-1	H_2O	BSA
BSA@ZIF-8_1.2μm	200	800	500	467	33
BSA@ZIF-C	350	650	145	531	324

"Metal" solution was added to "Ligand+BSA" solution slowly but steadily and mixed 3 times by inversion. The resulting solutions (Table S5) were then incubated at room temperature for 24 h. Then, the samples were centrifuged at 15,000 rpm for 5 min and the first supernatant was collected for protein quantification using Bradford assay (to calculate encapsulation efficiency). The sample was washed with 1 mL of Milli-Q water and resuspended by mixing with a spatula and 10 s of vortexing afterwards. The washing was repeated 3 times in total. Finally, the samples were air dried at room temperature for 24 h.

Table S5. Final concentrations of Zinc salt, mImH and BSA in each BSA@ZIF synthesis.

Sample	Zn (mM)	mImH (mM)	BSA (mg/mL)
BSA@ZIF-8_1.2μm	40	640	0.33
BSA@ZIF-C	28	185	3.24

Additionally, we synthesized BSA@ZIF-8 biocomposite with smaller particle size.[2] In this case, we added 200 mg of BSA to 10 mL of a mImH aqueous solution 1400 mM. The mImH/BSA solution was allowed to age for 1 h. Then, we added 10 mL of zinc acetate dihydrate, $Zn(OAc)_2·2H_2O$, aqueous solution 20 mM and this mixture was kept at room temperature for 16 h. When the reaction finished, we centrifugated at 5,000 rpm (30 min) and the supernatant was collected. This supernatant was further centrifuged at 14,000 rpm for 30 min and the resulting white precipitated was collected. This white solid was washed with water and allowed to air dry in order to obtain BSA@ZIF-8_120nm nanoparticles.

S2.1.6. Synthesis of Esterase@ZIF-8_60nm

In order to biomineralize the crystalline phase of sod ZIF-8 with a smaller particle size, we increased the ligand-to-metal ratio. For this purpose, we prepared the following stock solutions:

- $Zn(OAc)_2·2H_2O$ solution (160 mM). 0.3512 g of $Zn(OAc)_2·2H_2O$ is dissolved in 10 mL of H_2O milli-Q.
- mImH solution-1 (2560 mM). 2.1018 g of 2-methylimidazole is dissolved in 10 mL of H_2O milli-Q.
- Esterase solution (26.3 mg/ml). 52.6 mg of Esterase is dissolved in 2 mL of H_2O milli-Q.

In a typical experiment, the synthesis is carried out in 2 mL Eppendorf tubes (final reaction volume 2 mL), and 6 replicates are made. For each replicate, we prepared a "metal" and a "Ligand+esterase" solution in H_2O milli-Q according to table S6.

Table S6. Volumes (µL) of stock solutions to synthesize Esterase@ZIF-8_60nm biocomposite.

Sample	Metal Solution		Ligand+esterase solution		
	$Zn(OAc)_2 \cdot 2H_2O$	H_2O	mImH Solution-1	H_2O	Esterase
Esterase@ZIF-8_60nm	200	800	676	0	324

"Metal" solution was added to "Ligand+esterase" solution slowly but steadily and mixed 3 times by inversion. The resulting solutions (Table S7) were then incubated at room temperature for 24 h in static conditions. Then, the samples were centrifuged at 15,000 rpm for 5 min and the first supernatant was collected for protein quantification using Bradford assay (to calculate encapsulation efficiency, see S2.2.). The samples were washed with 1 mL of Milli-Q water and resuspended by mixing with a spatula and 10 s of vortexing afterwards. The washing was repeated 3 times in total. Finally, the samples were air dried at room temperature for 24 h.

Table S7. Final concentrations of Zinc salt, mImH and Esterase in Esterase@ZIF-8_60nm synthesis.

Sample	Zn (mM)	mImH (mM)	Esterase (mg/mL)
Esterase@ZIF-8_60nm	16	865	4.26

The encapsulation efficiency of this new material is 84 % and possesses a loading capacity of 0.20 mg esterase/mg biocomposite.

S2.1.7. Synthesis of Esterase adsorbed on ZIF

In order to highlight the amount of esterase encapsulated within the ZIFs during the biomimetic mineralization process, we prepared hybrid materials containing esterase adsorbed onto the ZIFs surface. These materials are referred to as Esterase-on-ZIF (Esterase-on-ZIF-8_150nm, Esterase-on-ZIF-L and Esterase-on-ZIF-C). The synthesis was carried out by incubating 20 mg of ZIF (ZIF-8_150 nm, ZIF-L or ZIF-C) in 2 mL of an aqueous esterase solution (4.26 mg/mL) for 24 h. Then, the materials were centrifuged (15,000 rpm, 5 min) and washed with Milli-Q H_2O (x3, 15,000 rpm, 5 min). Finally, the materials were air dried at room temperature.

S2.2. Evaluation of protein encapsulation efficiency.

S2.2.1. Encapsulation efficiency from the supernatant.

We evaluated protein (BSA or esterase) encapsulation of each Esterase/BSA@ZIF and Esterase-on-ZIF biocomposite material using the Bradford assay. In this case, we have quantified the encapsulated protein by difference with the protein remaining in the supernatants after each synthesis. After 24 h of synthesis, the solid product is separated via centrifugation (15,000 rpm for 5 min) and the supernatant (~ 2 mL) is recovered by micropipette. 33.3 µL of this supernatant is mixed with 1 mL of Bradford reagent. This mixture is shaken gently and incubated for 30 min at room temperature. Finally, we measured the absorbance at 590 nm in a UV-Vis spectrophotometer. All the experiments were performed in sixfold to calculate the encapsulation efficiency (EE) and protein loading capacity (LC) (see Table S8).

$$EE\ (\%) = \frac{[Total\ Protein\ added] - [Protein\ in\ supernatant]}{[Total\ Protein\ added]} \times 100$$

$$LC = \frac{mg\ protein\ encapsulated}{mg\ biocomposite}$$

S2.2.2. Encapsulation efficiency from digested ZIFs.

We also evaluated the encapsulation efficiency for Protein@ZIF materials by digesting the ZIF component of the synthesized Protein@ZIF biocomposite materials. For this purpose, 5 mg of each Protein@ZIF material was incubated in 1 mL of citrate buffer solution (0.1 M, pH = 5.5) for 24 h. Then, 33.3 µL of this solution is mixed with 1 mL of Bradford reagent. This mixture is shaken gently and incubated for 30 min at room temperature. Finally, we measure the absorbance at 590 nm in a UV-Vis spectrophotometer. All the experiments were performed in sixfold to calculate the encapsulation efficiency (EE) (see Table S8).

$$EE\ (\%) = \frac{[Protein\ in\ supernatant\ after\ digestion]}{[Total\ Protein\ added]}\ x\ 100$$

Table S8. Comparison of encapsulation efficiency and loading capacity for Protein@ZIF biocomposites between the supernatant and digested experiments

	Supernatant		Digested	
Sample	EE (%)	LC ($mg_{protein}/mg_{Biocomposite}$)	EE (%)	LC
Esterase@ZIF-8_120nm	76 ± 2	0.37 ± 0.01	11*	0.06*
Esterase@ZIF-C	96 ± 1	0.47 ± 0.03	87	0.43
Esterase@ZIF-L	74 ± 2	0.37 ± 0.01	76	0.38
BSA@ZIF-8_1.2µm	99 ± 1	0.50 ± 0.01	-	-
BSA@ZIF-8_120nm	70 ± 3	0.44 ± 0.20	-	-
BSA@ZIF-C	36 ± 4.7	0.11 ± 0.04	-	-
Esterase-on-ZIF-8_150nm	6 ± 3	0.026 ± 0.010	-	-
Esterase-on-ZIF-L	10 ± 4	0.044 ± 0.016	-	-
Esterase-on-ZIF-C	5 ± 1	0.020 ± 0.003	-	-

* Digested values calculated for Esterase@ZIF-8_120nm biocomposite does not match with the supernatant because of greater stability of sod ZIF-8 phase in citrate buffer (see results from TGA).

Table S9. Summary of reported Esterase Loading Capacities (LC) in Metal–Organic Frameworks.

Enzyme	MOF	Synthetic method	Reported LC (wt%)	Ref.
Esterase from porcine liver	CaIDC	Biomimetic mineralization	4.2	[3]
Esterase from porcine liver	ZIF-90	Biomimetic mineralization	19.5	[4]
Esterase from porcine liver	MAF-6	Biomimetic mineralization	3.1	[4]
Esterase from porcine liver	ZIF-8	Biomimetic mineralization	4.2	[4]
Aryloxyphenoxypropionate -hydrolase, esterase	ZIF-10	Biomimetic mineralization	15.2	[5]
Aryloxyphenoxypropionate -hydrolase, esterase	ZIF-8	Biomimetic mineralization	19.7	[5]

Esterase from *A. acidocaldarius*	NU-1000	Post-synthetic infiltration	17.3	6
Esterase from porcine liver	ZIF-8	Biomimetic mineralization	37 (120nm) 20 (60 nm)	This work
Esterase from porcine liver	ZIF-L	Biomimetic mineralization	37	This work
Esterase from porcine liver	ZIF-C	Biomimetic mineralization	47	This work

S2.3. Nerve agent simulant degradation.

S2.3.1. DIFP degradation by ZIFs and Protein@ZIF biocomposites

The hydrolytic degradation of nerve agent simulant diisopropylfluorophosphate (DIFP) was evaluated using gas chromatography-flame ionization detector (GC-FID) analysis. In a typical experiment, we incubated 2.5 μL of DIFP (0.015 mmol) with Esterase@ZIFs, BSA@ZIFs and ZIFs materials using a DIFP:ZIF ratio of 1:6 (0.084 mmol of ZIF), together with 1.08 μL dimethylformamide (DMF, 0.015 mmol, used as an internal reference) in 0.5 mL of Tris−HCl buffer solution (0.1 M, pH = 7.4). The reaction was stirred at room temperature, and DIFP degradation was monitored via GC-FID analysis at different times (Table S10).

Therefore, for the Esterase@ZIFs and BSA@ZIFs hybrids, we considered the proportion of ZIF present in the hybrids previously calculated (see Table S8).

Table S10. Half-life times and kinetic constants values for diisopropylfluorophosphate degradation reaction.

Sample	$t_{1/2}$ (min)	Kinetic constant
Esterase@ZIF-8_120nm	8.0	0.054 min^{-1}
Esterase@ZIF-8_60nm	2.1	0.154 min^{-1}
ZIF-8_150nm	40	0.0224 min^{-1}
Esterase@ZIF-C	7.2	0.044 min^{-1}
ZIF-C	1382	0.0005 min^{-1}
Esterase@ZIF-L	30	0.017 min^{-1}
ZIF-L	75	0.0065 min^{-1}
BSA@ZIF-8_1.2μm	174	0.0041 min^{-1}
BSA@ZIF-8_120nm	22	0.0283 min^{-1}
BSA@ZIF-C	78	0.0101 min^{-1}
Esterase	2490	0.014 $M^{-1}\cdot min^{-1}$

Table S11. Reported organophosphate degradation by MOF-based biocomposites

Biocomposite	Organophosphorous substrate	Half-life time (min)	Ref.
Organophosphorus acid anhydrolase (OPAA)@PCN-128y	Diisopropylfluorophos phate	~ 2-5	7
OPAA@PCN-128y	Soman	20	7
OPAA@NU-1003-300nm	Diisopropylfluorophos phate	Not mentioned (100 % deg. after 2 min)	8
OPAA@NU-1003-300nm	Soman	2 (initial half-life)	8

S2.3.2. Esterase+ZIFs physical mixture.

In order to determine the synergistic effect on Esterase@ZIF biocomposites, we carried out DIFP degradation experiments of the physical mixture between ZIFs and esterase.

In a typical experiment, we incubated 2.5 μL of DIFP (0.015 mmol) with a mixture of esterase + ZIF using the same proportions of each component as in Esterase@ZIF experiments, together with 1.08 μL dimethylformamide (DMF, 0.015 mmol) in 0.5 mL of Tris−HCl buffer solution (0.1 M, pH = 7.4). The reaction was stirred at room temperature, and DIFP degradation was monitored via GC-FID analysis at different times.

S2.3.3. Hot filtration test.

To demonstrate the heterogeneity of the process, we performed hot filtration tests. For this purpose, in the middle of the reaction course between Esterase@ZIF biocomposites and DIFP, the reaction solid was filtered.

In a typical experiment, we incubated 2.5 μL of DIFP (0.015 mmol) with Esterase@ZIF biocomposites (0.084 mmol of ZIF), together with 1.08 μL dimethylformamide (DMF, 0.015 mmol, used as an internal reference) in 0.5 mL of Tris−HCl buffer solution (0.1 M, pH = 7.4). When DIFP degradation is around 50 %, this solution is filtered. After this, the presence of DIFP in the buffer solution was monitored via GC-FID analysis at different times.

S2.3.4. Control experiments

We also carried out some control experiments to demonstrate that DIFP degradation is carried out by the studied materials.

On the one hand, we checked DIFP concentration in Tris-HCl buffer solution throughout the whole experiment. In a typical experiment, we incubated 2.5 μL of DIFP (0.015 mmol) with 1.08 μL dimethylformamide (DMF, 0.015 mmol, used as an internal reference) in 0.5 mL of Tris−HCl buffer solution (0.1 M, pH = 7.4). The presence of DIFP in the buffer solution was monitored via GC-FID analysis at different times.

On the other hand, we monitored the possible degradation of DIFP by ZIFs precursors: 2-methylimidazole and $Zn(OAc)_2{\cdot}2H_2O$. In a typical experiment, we incubated 2.5 μL of DIFP (0.015 mmol) with 0.084 mmol of mImH/$Zn(OAc)_2{\cdot}2H_2O$, together with 1.08 μL dimethylformamide (DMF, 0.015 mmol, used as an internal reference) in 0.5 mL of Tris−HCl buffer solution (0.1 M, pH = 7.4). The presence of DIFP in the buffer solution was monitored via GC-FID analysis at different times.

S2.4. Enzymatic Activity.

S2.4.1. Determination of Michaelis-Menten constant for AChE and Esterase enzymes.

The enzymatic activity was determined by the indoxyl acetate colorimetric method. [9]In this method, the concentration of the enzymatic product, indigo blue, was determined via UV−vis spectroscopy at a wavelength of 620 nm, with the molar extinction coefficient (ϵ) set at 22,140 $M^{-1}cm^{-1}$. This approach was chosen over the traditional Ellman method due to the susceptibility of acetylthiocholine to nucleophiles, such as oximes or imidazolate ligands, which can result in false-positive readings.

In a typical experiment, 24-well culture plates were filled with 925 µL of Tris−HCl (0.1 M, pH = 7.4) buffer solution, 25 µL of an Esterase/AChE aqueous solution (75 U/mL); and they were incubated for 30 min at 37 ºC. Afterwards, we added 50 µL of indoxyl acetate solution in isopropanol with different concentrations (10^{-8} M, 10^{-7} M, 10^{-6} M, 10^{-5} M, 10^{-4} M, $2.5 \cdot 10^{-4}$ M, $5 \cdot 10^{-4}$ M, $7.5 \cdot 10^{-4}$ M, 10^{-3} M, $2.5 \cdot 10^{-3}$ M, $5 \cdot 10^{-3}$ M, $7.5 \cdot 10^{-3}$ M and 10^{-2} M) and the mixtures were incubated for an additional 30 min. Finally, 3.33 mL of dimethyl sulfoxide (DMSO) was added to solubilize the enzymatic product (indigo blue) and stop the reaction. The absorbance of the solutions was measured at λ = 620 nm.

S2.4.2. Inhibition of enzymatic activity by diisopropylfluorophosphate.

We evaluated Esterase and AChE inhibition by nerve agent simulant diisopropylfluorophosphate (DIFP) under simulated biological conditions. For this purpose, we incubated free Esterase/AChE solutions with different concentrations of DIFP. In a typical experiment, 24-well culture plates were filled with 825 µL of Tris−HCl (0.1 M, pH = 7.4) buffer solution, 25 µL of an Esterase/AChE aqueous solution (75 U/mL); and they were incubated for 30 min at 37 ºC. Then, we added 100 µL of aqueous DIFP solution with different concentrations ($5 \cdot 10^{-7}$ M, 10^{-6} M, $5 \cdot 10^{-6}$ M, 10^{-5} M, 10^{-4} M, 10^{-3} M, 10^{-2} M and 10^{-1} M) and this mixture was incubated for 1 h. Afterwards, 50 µL of indoxyl acetate solution in isopropanol (3.0 mM) was added and the mixtures were incubated for an additional 30 min. Finally, 3.33 mL of dimethyl sulfoxide (DMSO) was added to solubilize the enzymatic product (indigo blue) and stop the reaction. The absorbance of the solutions was measured at λ = 620 nm. The corresponding inhibition percentage was calculated according to the following equation:

$$\text{Inhibition (\%)} = 100 - \left(\frac{Inh.\ Enzymatic\ Activity}{Free\ Enzymatic\ Activity}\right) \text{ x } 100$$

Inh. Enzymatic Activity = Enzymatic activity for Esterase/AChE incubated with DIFP at different concentrations

Free Enzymatic Activity = Enzymatic activity for free Esterase/AChE at 37 ºC.

S2.4.3. Retained enzymatic activity after Esterase biomineralization.

We determined the retained enzymatic activity after Esterase@ZIF biocomposites synthesis. To do this, we first determined the enzymatic activity of free Esterase at 37 °C. In a typical experiment, 24-well culture plates were filled with 925 µL of Tris−HCl (0.1 M, pH = 7.4) buffer solution, 25 µL of an Esterase aqueous solution (75 U/mL) and they were incubated for 1 h at 37 ºC. Then, 50 µL of indoxyl acetate solution in isopropanol (3.0 mM) was added and the mixtures were incubated for an additional 30 min. Finally, 3.33 mL of dimethyl sulfoxide (DMSO) was added to solubilize the enzymatic product (indigo blue) and stop the reaction. The absorbance of the solutions was measured at λ = 620 nm.

Secondly, we determined the enzymatic activity of Esterase@ZIF biocomposites. We incubated Esterase@ZIFs materials in 1 mL of Tris−HCl (0.1 M, pH = 7.4) buffer solution (1 h at 37 ºC) so that the final concentration of Esterase in the suspension was 1.87 U/mL (we considered the esterase loading capacity in each Esterase@ZIF calculated by the Bradford assay; see Table S8). Afterwards, 50 µL of indoxyl acetate solution in isopropanol (3.0 mM) was added and the mixtures were incubated for an additional 30 min. Then, we centrifuged this suspension, and the supernatants were added to 24-well culture plates. Finally, 3.33 mL of dimethyl sulfoxide (DMSO) was added to solubilize the enzymatic product (indigo blue) and stop the reaction. The absorbance of the solutions was measured at λ = 620 nm.

Additionally, we have also performed enzymatic activity assays for Zeolitic Imidazolate Frameworks (ZIF-8_150nm, ZIF-L and ZIF-C) as control experiments. In a typical experiment, we incubated ZIFs materials in 1 mL of Tris−HCl (0.1 M, pH = 7.4) buffer solution (1 h at 37 ºC) so that the final concentration of each ZIF in the suspension was the same as ZIF concentration in Esterase@ZIF experiments (see Table S8). Afterwards, 50 µL of indoxyl acetate solution in isopropanol (3.0 mM) was added and the mixtures were incubated for an additional 30 min. Then, we centrifuged this suspension and added the supernatants to 24-well culture plates. Finally, 3.33 mL of dimethyl sulfoxide (DMSO) was added to solubilize the enzymatic product (indigo blue) and stop the reaction. The absorbance of the solutions was measured at λ = 620 nm.

$$\text{Retained Enzymatic Activity after Synthesis (\%)} = \frac{Enzymatic\ Activity\ Bio/ZIFs}{Free\ Enzymatic\ Activity} \times 100$$

Enzymatic Activity Bio/ZIFs = Enzymatic activity for Esterase@ZIF biocomposites or ZIFs materials at 37 ºC

Free Enzymatic Activity = Enzymatic activity for free Esterase at 37 ºC

S2.4.4. Reactivation of the enzymatic activity by 2-methylimidazole.

We evaluated the reactivation of the enzymatic activity of AChE by 2-methylimidazole linker (mImH) under simulated biological conditions. For this purpose, we incubated 50 % DIFP inhibited AChE solutions with mImH at different concentrations. In a typical experiment, 24-well culture plates were filled with 725 µL of Tris−HCl (0.1 M, pH = 7.4) buffer solution, 25 µL of AChE aqueous solution (75 U/mL) and 100 µL of DIFP aqueous solution corresponding to 50% inhibited enzyme (see S2.4.2); and they were incubated for 1 h at 37 ºC. Then, we added 100 µL of mImH solution with different concentrations (10^{-8} M, 10^{-7} M, 10^{-6} M, 10^{-5} M, 10^{-4} M, 10^{-3} M, 10^{-2} M and 10^{-1} M) and this mixture was incubated for 1 h. Afterwards, 50 µL of indoxyl acetate solution in isopropanol (3.0 mM) was added and the mixtures were incubated for an additional 30 min. Finally, 3.33 mL of dimethyl sulfoxide (DMSO) was added to solubilize the enzymatic product (indigo blue) and stop the reaction. The absorbance of the solutions was measured at λ = 620 nm. The corresponding reactivation percentage was calculated according to the following equation:

$$\text{Reactivation (\%)} = \left(\frac{React.\ Enzymatic\ Activity - Enzymatic\ Activity\ 50\%}{Free\ Enzymatic\ Activity - Enz\qquad Activity\ 50\%}\right) \times 100$$

React. Enzymatic Activity = Enzymatic activity for AChE incubated with mImH at different concentrations

Enzymatic Activity 50 % = Enzymatic activity for 50% inhibited AChE at 37 ºC.

Free Enzymatic Activity = Enzymatic activity for free AChE at 37 ºC.

S2.4.5. Retained enzymatic activity with temperature.

Here we studied how temperature affects the enzymatic activity of free Esterase and Esterase@ZIF biocomposites. Regarding free esterase, 24-well culture plates were filled with 925 μL of Tris−HCl (0.1 M, pH = 7.4) buffer solution, 25 μL of an Esterase aqueous solution (75 U/mL) and they were incubated at two different times (30 min and 1 h) and different temperatures (60 ºC, 70 ºC, 80 ºC and 90 ºC). Then, 50 μL of indoxyl acetate solution in isopropanol (3.0 mM) was added and the mixtures were incubated for an additional 30 min. Finally, 3.33 mL of dimethyl sulfoxide (DMSO) was added to solubilize the enzymatic product (indigo blue) and stop the reaction. The absorbance of the solutions was measured at λ = 620 nm.

Regarding the Esterase@ZIF biocomposites, we incubated them (1.87 U/mL Esterase final concentration) in 1 mL of Tris−HCl (0.1 M, pH = 7.4) buffer solution at two different times (30 min and 1 h) and different temperatures (60 ºC, 70 ºC, 80 ºC and 90 ºC). Then, 50 μL of indoxyl acetate solution in isopropanol (3.0 mM) was added and the mixtures were incubated for an additional 30 min at room temperature. Finally, 3.33 mL of dimethyl sulfoxide (DMSO) was added to solubilize the enzymatic product (indigo blue) and stop the reaction. The absorbance of the solutions was measured at λ = 620 nm. The corresponding retained enzymatic activity with temperature was calculated according to the following equation:

$$\text{Retained Enzymatic Activity with Temperature (\%)} = \frac{Enzymatic\ Activity\ Temp.}{Free\ Enzymatic\ Activity} \times 100$$

Enzymatic Activity Temp. = Enzymatic activity for free Esterase or Esterase@ZIF biocomposites at a specific temperature (60, 70, 80 and 90 ºC).

Free Enzymatic Activity = Enzymatic activity for free Esterase or Esterase@ZIF biocomposites at 37 ºC.

S2.4.6. Retained enzymatic activity at Inhibitory Concentrations.

We then evaluated Esterase@ZIF biocomposites retained enzymatic activity towards DIFP inhibitory concentrations IC50 ($5\cdot10^{-7}$ M) and IC90 (10^{-4} M). In a typical experiment, we incubated 850 μL of Tris−HCl (0.1 M, pH = 7.4) buffer solution, 1.87 U/mL of each Esterase@ZIF material and 100 μL of DIFP aqueous solution ($5\cdot10^{-6}$ M for IC50 and 10^{-3} M for IC90) for 1 h at 37 ºC. Afterwards, 50 μL of indoxyl acetate solution in isopropanol (3.0 mM) was added and the mixtures were incubated for an additional 30 min. Then, we centrifuged this suspension and added the supernatants to 24-well culture plates. Finally, 3.33 mL of dimethyl sulfoxide (DMSO) was added to solubilize the enzymatic product (indigo blue) and stop the reaction. The absorbance of the solutions was measured at λ = 620 nm. The corresponding retained enzymatic activity percentage was calculated according to the following equation:

$$\text{Retained Enzymatic Activity at IC (\%)} = \frac{Enzymatic\ Activity\ IC}{Enzymatic\ Activity\ Bio} \times 100$$

Enzymatic Activity IC = Enzymatic activity for Esterase@ZIF biocomposites at different DIFP Inhibitory Concentrations.

Enzymatic Activity Bio= Enzymatic activity for Esterase@ZIF biocomposites at 37 ºC.

We also performed control experiments studying the retained Esterase enzyme activity against DIFP inhibitory concentrations IC50 ($5 \cdot 10^{-7}$ M) and IC90 (10^{-4} M) using a physical mixture of ZIFs and Esterase. In a typical experiment, we incubated 25 µL of an Esterase aqueous solution (75 U/mL) and the different ZIFs in 825 µL of Tris−HCl (0.1 M, pH = 7.4) buffer solution (1 h at 37 ºC) so that the final concentration of each ZIF (ZIF-8_150nm, ZIF-L or ZIF-C) in the suspension was the same as ZIF concentration in Esterase@ZIF experiments. Then, we added 100 µL of DIFP solution ($5 \cdot 10^{-6}$ M for IC50 and 10^{-3} M for IC90) and this mixture was incubated for 1 h. Then, we centrifuged this suspension and added the supernatants to 24-well culture plates. Afterwards, 50 µL of indoxyl acetate solution in isopropanol (3.0 mM) was added and the mixtures were incubated for an additional 30 min. Finally, 3.33 mL of dimethyl sulfoxide (DMSO) was added to solubilize the enzymatic product (indigo blue) and stop the reaction. The absorbance of the solutions was measured at λ = 620 nm. The corresponding retained enzymatic activity percentage was calculated according to the following equation:

$$\text{Retained Enzymatic Activity at IC (\%)} = \frac{Enzymatic\ Activity\ IC}{Free\ Enzymatic\ Activity} \times 100$$

Enzymatic Activity IC = Enzymatic activity for Esterase+ZIF physical mixture at different DIFP Inhibitory Concentrations.

Free Enzymatic Activity = Enzymatic activity for free Esterase at 37 ºC.

S2.4.7. Detoxification experiments

In this case, additional enzymatic assays were carried out to compare Esterase@ZIFs and ZIFs ability to reduce the inhibitory effect of DIFP on AChE activity. In a typical experiment, 0.084 mmol of Esterase@ZIFs (Esterase@ZIF-8_120nm, Esterase@ZIF-L and Esterase@ZIF-C) were mixed with DIFP (0.029 M) in 0.5 mL of Tris−HCl (0.1 M, pH = 7.4) and stirred at room temperature. After different time frames (10 min, 30 min, 60 min, 4 h, 12 h and 24 h) the supernatant was separated from the solid by centrifugation (15,000 rpm × 5 min) and diluted 580 times. Then, a 24-well culture plate was filled with 825 µL of Tris−HCl (0.1 M, pH = 7.4), 25 µL of an aqueous solution of AChE (75 U/mL), and 100 µL of the diluted supernatants. The mixtures were then incubated for 1 h at 37 °C. Afterward, 50 µL of indoxyl acetate solution in isopropanol (3.0 mM) was added and the mixtures were incubated for an additional 30 min. Finally, 3.33 mL of DMSO was added to solubilize the enzymatic product (indigo blue) and the absorbance of the solutions at λ = 620 nm was collected. Additional experiments were carried out to estimate the enzymatic activity of uninhibited AChE and 50 % inhibited AChE with DIFP, following the same protocol as in the inhibition assays (see S2.4.2.). The corresponding detoxification percentage was calculated according to the following equation:

$$\text{Detoxification (\%)} = \left(\frac{Enzymatic\ Activity\ Detox. - Enzymati\ \ Activity\ 50\%}{Free\ Enzymatic\ Activity - Enzymatic\ Activity\ 50\%} \right) \times 100$$

Enzymatic Activity Detox. = Enzymatic activity for AChE after incubation with Esterase@ZIFs/ZIFs

Enzymatic Activity 50 % = Enzymatic activity for 50% inhibited AChE at 37 ºC.

Free Enzymatic Activity = Enzymatic activity for uninhibited AChE at 37 ºC.

The same experiment was carried out with ZIF-8_150nm. In this case, 0.084 mmol of ZIF-8_150nm were mixed with DIFP (0.029 M) in 0.5 mL of Tris−HCl (0.1 M, pH = 7.4) and stirred at room temperature.

S2.5. Cytotoxicity assay

We evaluated cytotoxicity of esterase, ZIFs and Esterase@ZIFs materials towards human neuroblastoma cell culture SH-SY5Y (reference number: ECACC Nº: 94030304; batch nº 98I033), obtained from the Centre of Scientific Instrumentation of the University of Granada. These cells were cultivated as a suspension in Ham's F12:EMEM (EBSS) (1:1) + 2mM Glutamine + 1% Non-Essential Amino Acids (NEAA) + 15% Fetal Bovine Serum (FBS) at 37 °C in a humidified atmosphere with 5% CO_2 and maintained using standard cell culture techniques. Cell viability was evaluated by treating SH-SY5Y cells with increasing concentrations ($5·10^{-4}$ mg/mL, 10^{-3} mg/mL, $5·10^{-3}$ mg/mL, 10^{-2} mg/mL, $5·10^{-2}$ mg/mL, 10^{-1} mg/mL and $5·10^{-1}$ mg/mL) of esterase, ZIFs (ZIF-8_150nm, ZIF-L and ZIF-C) and Esterase@ZIFs (Esterase@ZIF-8_120nm, Esterase@ZIF-L and Esterase@ZIF-C) after 24h of exposure colorimetric MTS assay.

S3. Experimental results

S3.1. Characterization of Esterase@ZIF biocomposites

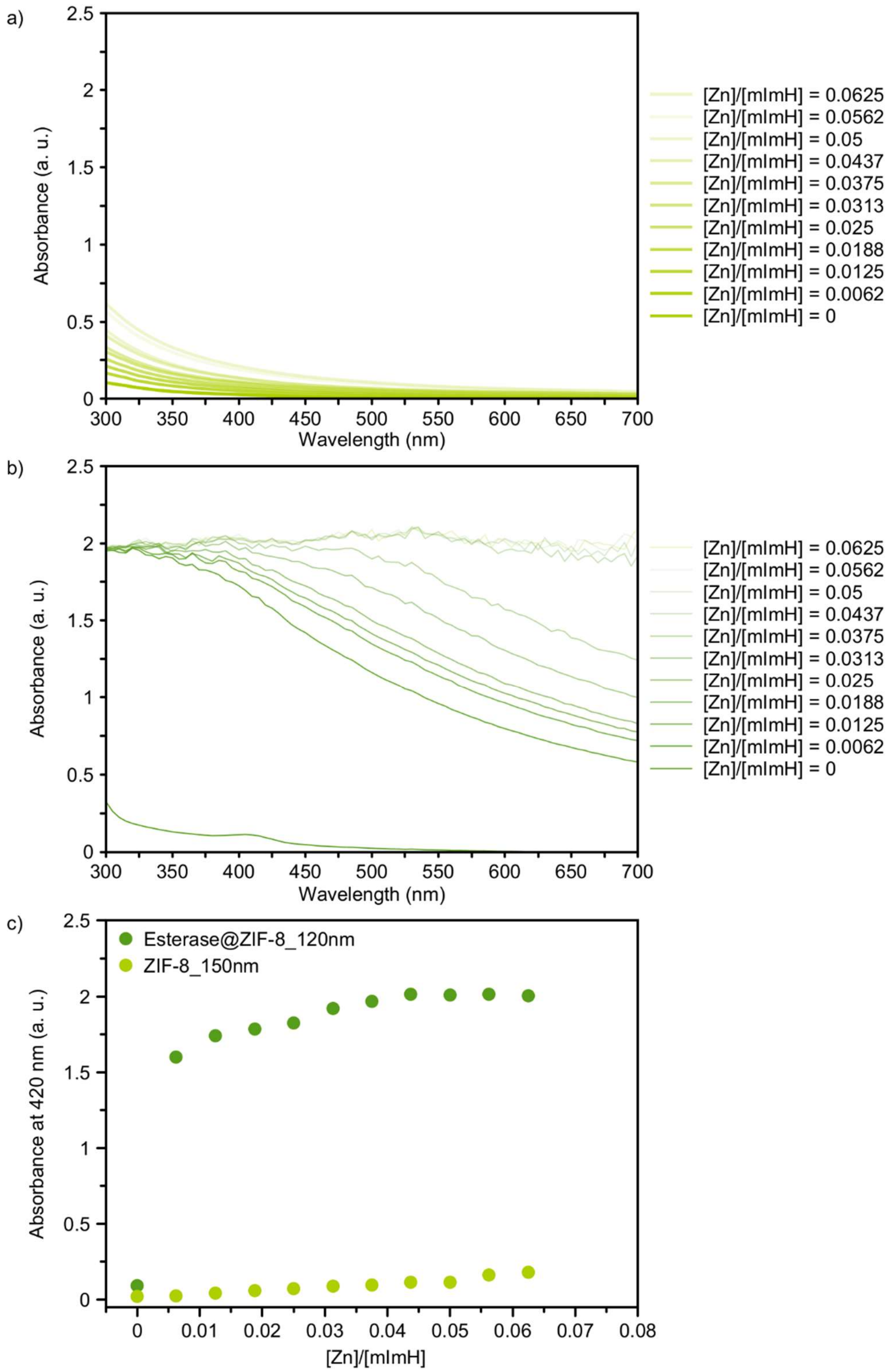

Figure S1. Turbidimetry assay for ZIF-8 formation. (a) UV-vis absorption spectra during ZIF-8 synthesis in the absence of esterase, with a total of 10 injections. (b) UV-vis absorption spectra during Esterase@ZIF-8_120nm synthesis in the presence of esterase, with a total of 10 injections. (c) Change in absorption at 420 nm during ZIF-8 mineralization in the absence or presence of enzyme, as determined from the turbidimetry assays.

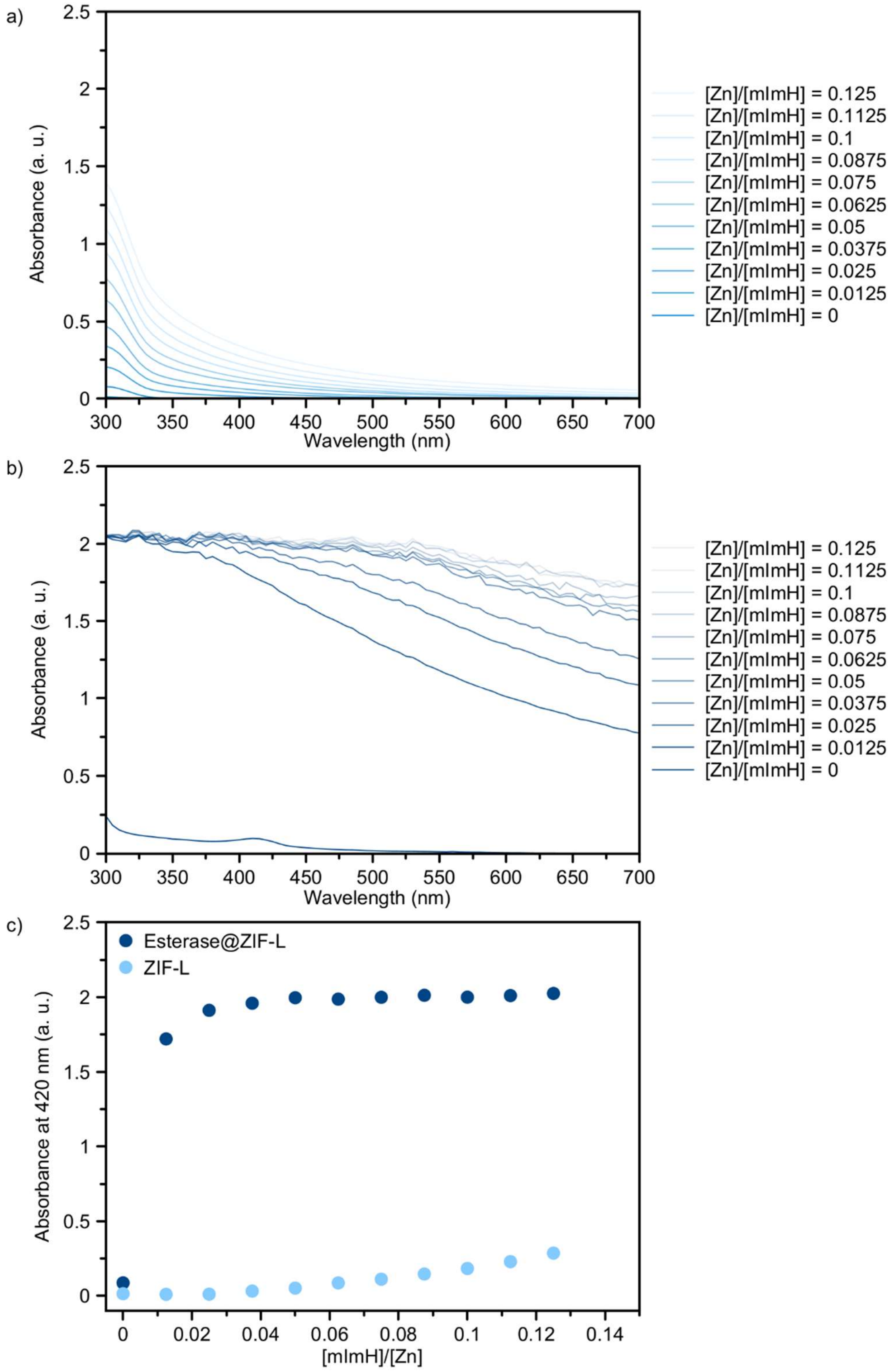

Figure S2. Turbidimetry assay for ZIF-L formation. (a) UV-vis absorption spectra during ZIF-L synthesis in the absence of esterase, with a total of 10 injections. (b) UV-vis absorption spectra during Esterase@ZIF-L synthesis in the presence of esterase, with a total of 10 injections. (c) Change in absorption at 420 nm during ZIF-L mineralization in the absence or presence of enzyme, as determined from the turbidimetry assays.

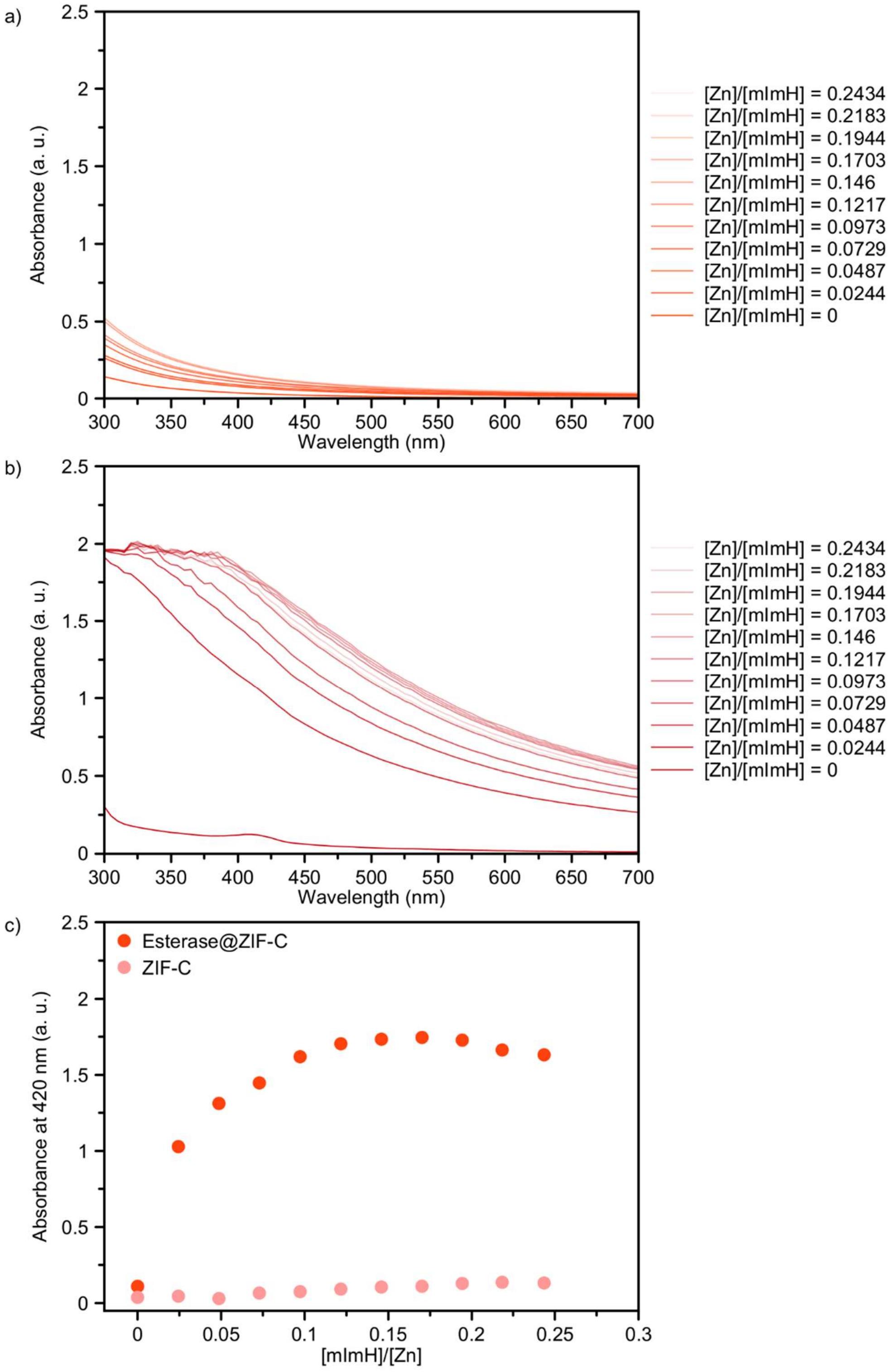

Figure S3. Turbidimetry assay for ZIF-C formation. (a) UV-vis absorption spectra during ZIF-C synthesis in the absence of esterase, with a total of 10 injections. (b) UV-vis absorption spectra during Esterase@ZIF-C synthesis in the presence of esterase, with a total of 10 injections. (c) Change in absorption at 420 nm during ZIF-C mineralization in the absence or presence of enzyme, as determined from the turbidimetry assays.

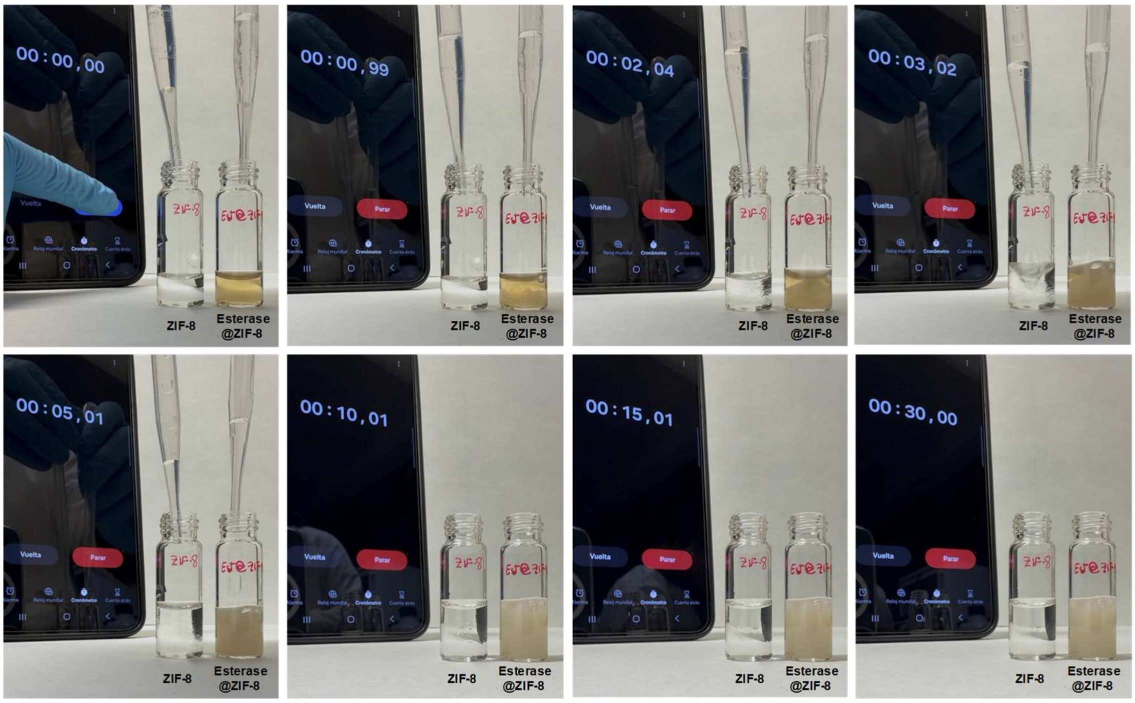

Figure S4. Sequential video frames illustrating the rapid formation of ZIF-8 via a biomimetic mineralization strategy. Right vial (Esterase@ZIF-8_120nm), reaction carried out by adding Zn (80 mM) metal solution into the aqueous mImH (1280 mM) + esterase (8.52 mg/mL) solution. Left vial (ZIF-8), control reaction containing the same precursor solutions in the absence of esterase. In the presence of the esterase, the rapid biomineralization of the MOF leads to the immediate formation of a precipitate. In contrast, in the absence of esterase, the reaction is much slower, and the solution remains transparent.

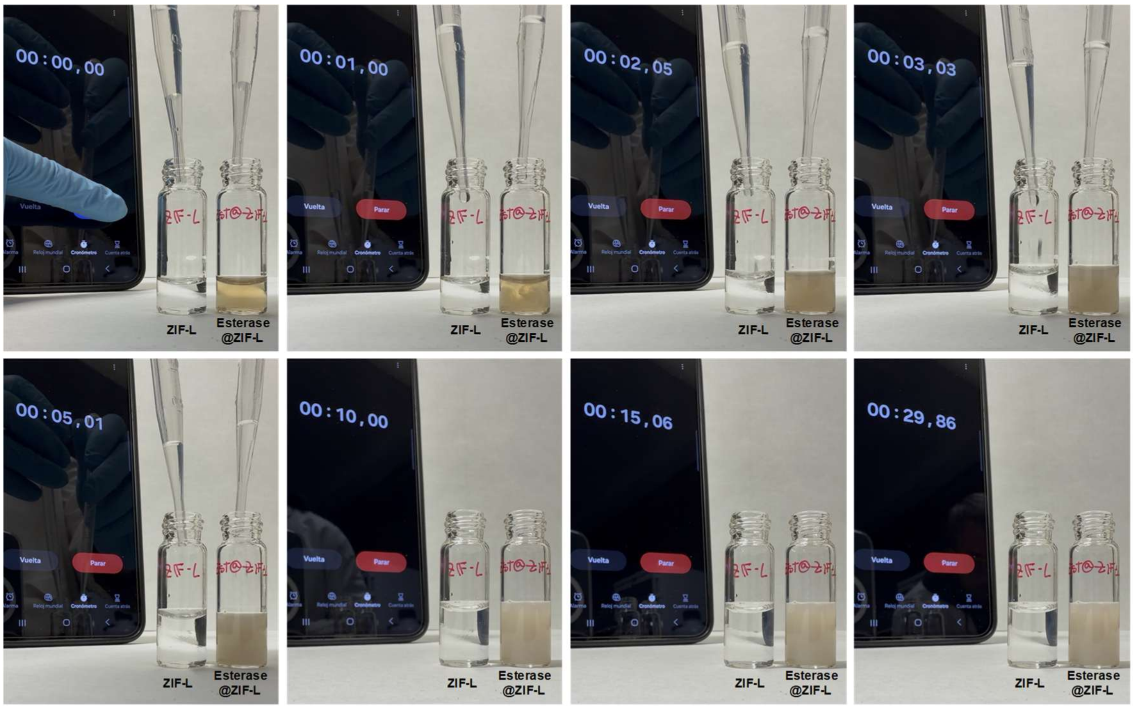

Figure S5. Sequential video frames illustrating the rapid formation of ZIF-L via a biomimetic mineralization strategy. Right vial (Esterase@ZIF-L), reaction carried out by adding Zn metal solution (50 mM) into the aqueous mImH (400 mM) + esterase (8.52 mg/mL) solution. Left vial (ZIF-L), control reaction containing the same precursor solutions in the absence of esterase. In the presence of the esterase, the rapid biomineralization of the MOF leads to the immediate formation of a precipitate. In contrast, in the absence of esterase, the reaction is much slower, and the solution remains transparent.

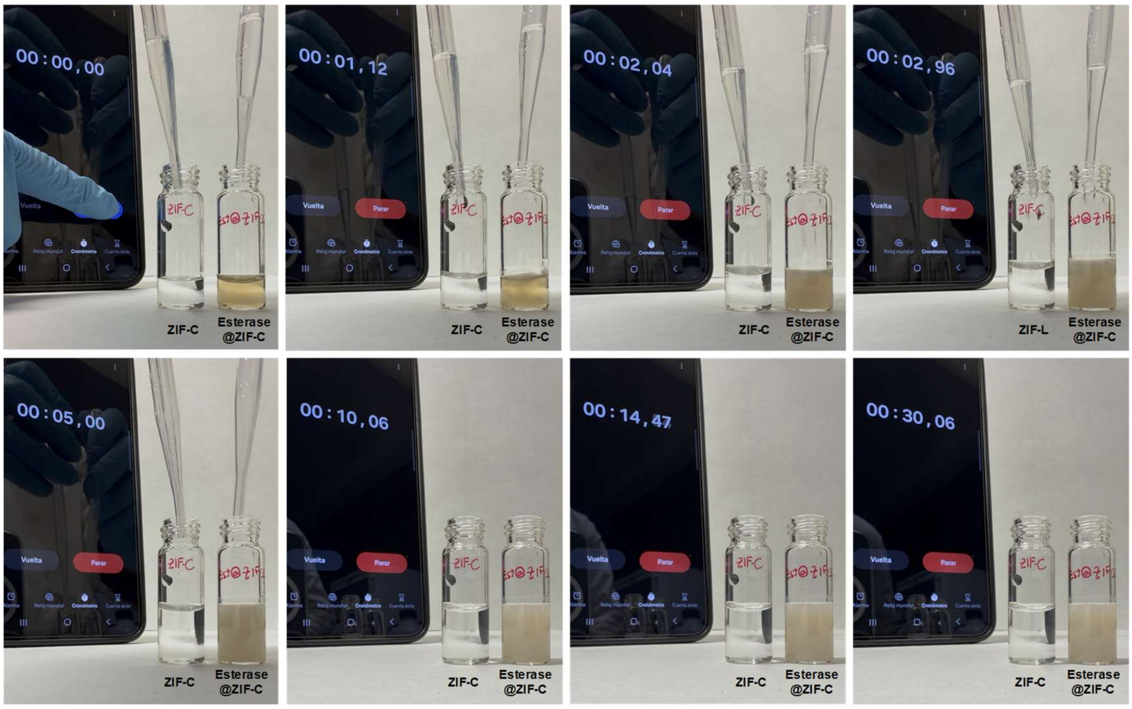

Figure S6. Sequential video frames illustrating the rapid formation of ZIF-C via a biomimetic mineralization strategy. Right vial (Esterase@ZIF-C), reaction carried out by adding Zn (145 mM) metal solution into the aqueous mImH (548 mM) + esterase (8.52 mg/mL) solution. Left vial (ZIF-C), control reaction containing the same precursor solutions in the absence of esterase. In the presence of the esterase, the rapid biomineralization of the MOF leads to the immediate formation of a precipitate. In contrast, in the absence of esterase, the reaction is much slower, and the solution remains transparent.

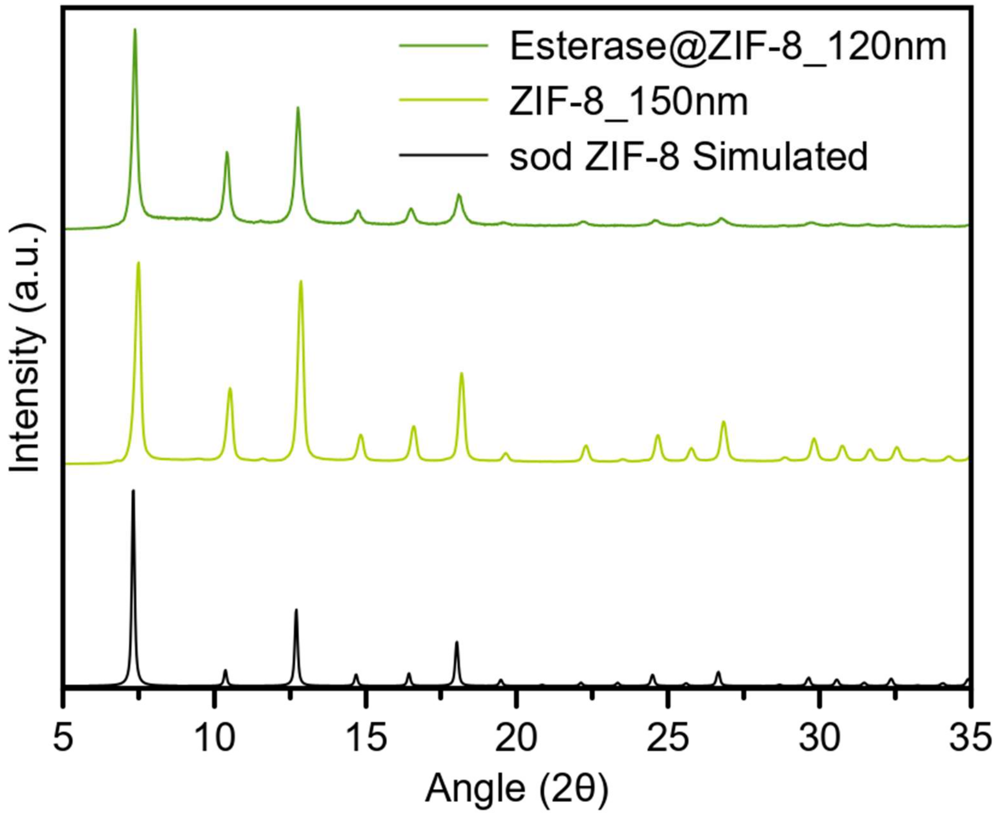

Figure S7. Powder X-Ray Diffraction patterns of Esterase@ZIF-8_120nm biocomposite (experimental, green), ZIF-8_150nm (experimental, light green) and simulated sod ZIF-8 (calculated, black).

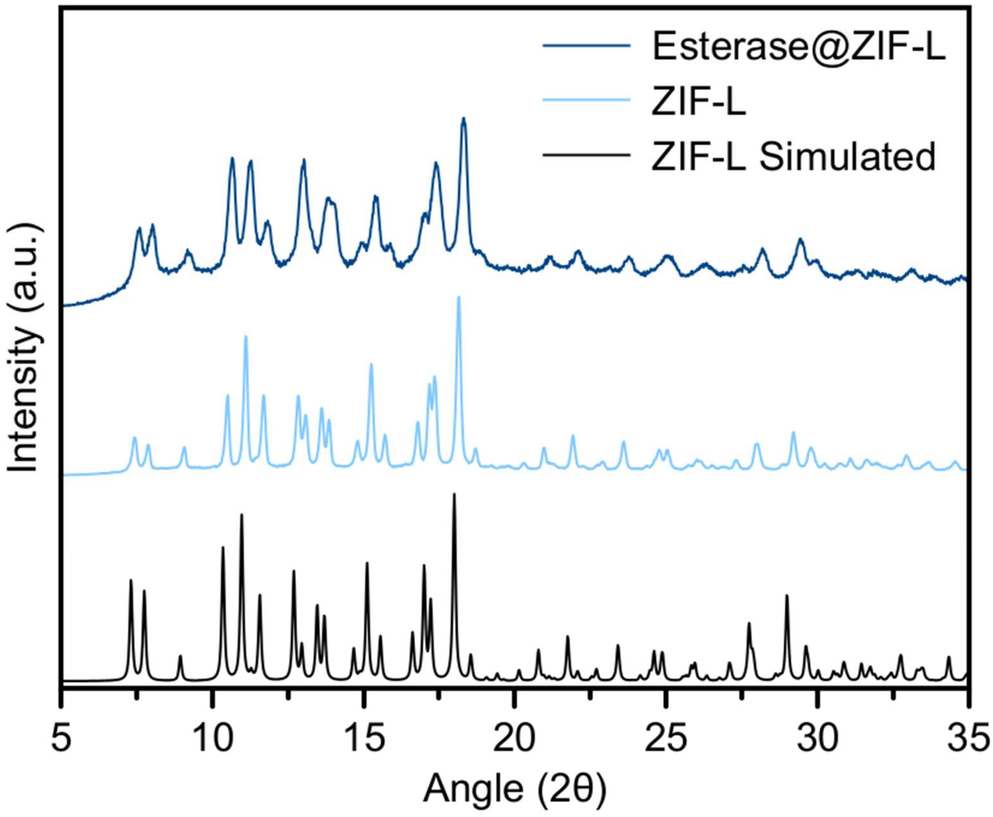

Figure S8. Powder X-Ray Diffraction patterns of Esterase@ZIF-L biocomposite (experimental, dark blue), ZIF-L (experimental, light blue) and simulated ZIF-L (calculated, black).

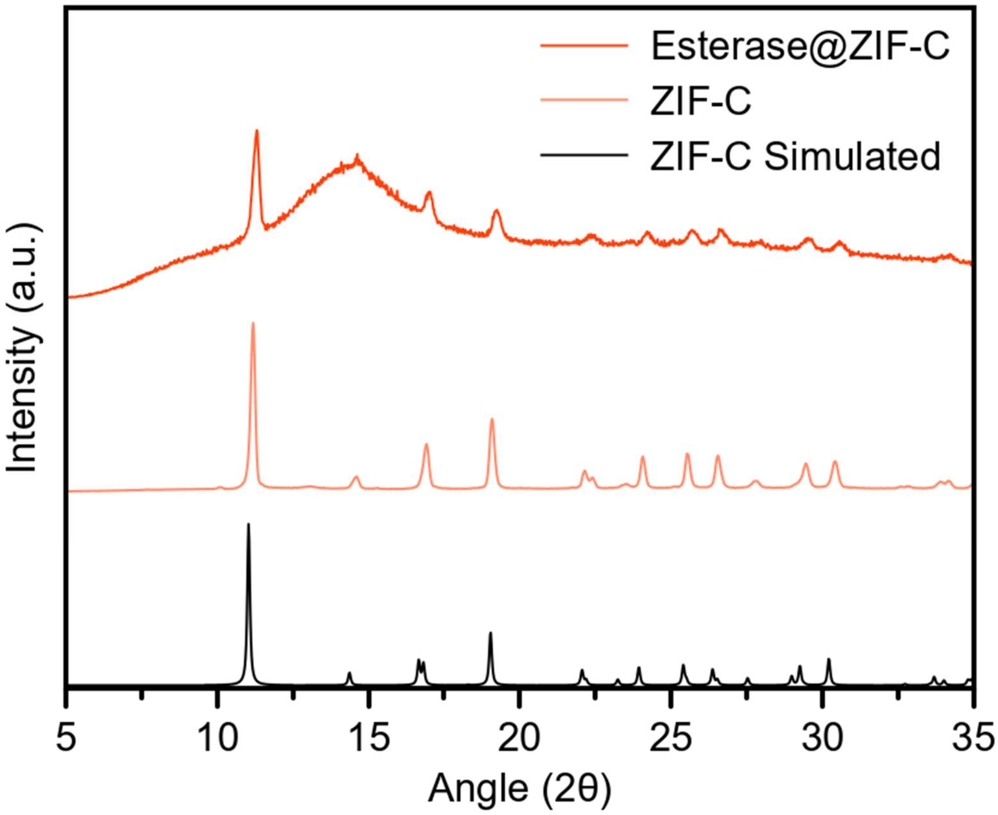

Figure S9. Powder X-Ray Diffraction patterns of Esterase@ZIF-C biocomposite (experimental, red), ZIF-C (experimental, light red) and simulated ZIF-C (calculated, black).

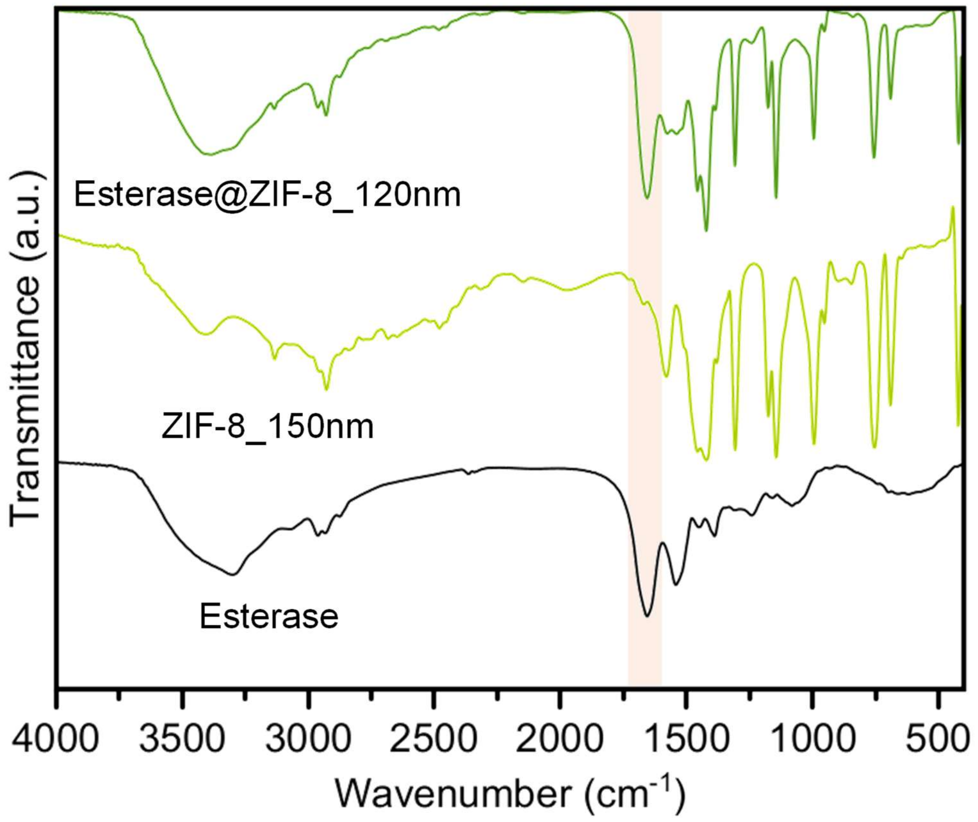

Figure S10. Fourier Transformed Infrared spectra (FTIR) of Esterase (black), ZIF-8_150nm (light green) and Esterase@ZIF-8_120nm (dark green) materials. The analysis of the FTIR data confirms the presence of the characteristic Amide I band (1700-1610 cm^{-1}) of the peptide backbone of Esterase highlighted in light orange.

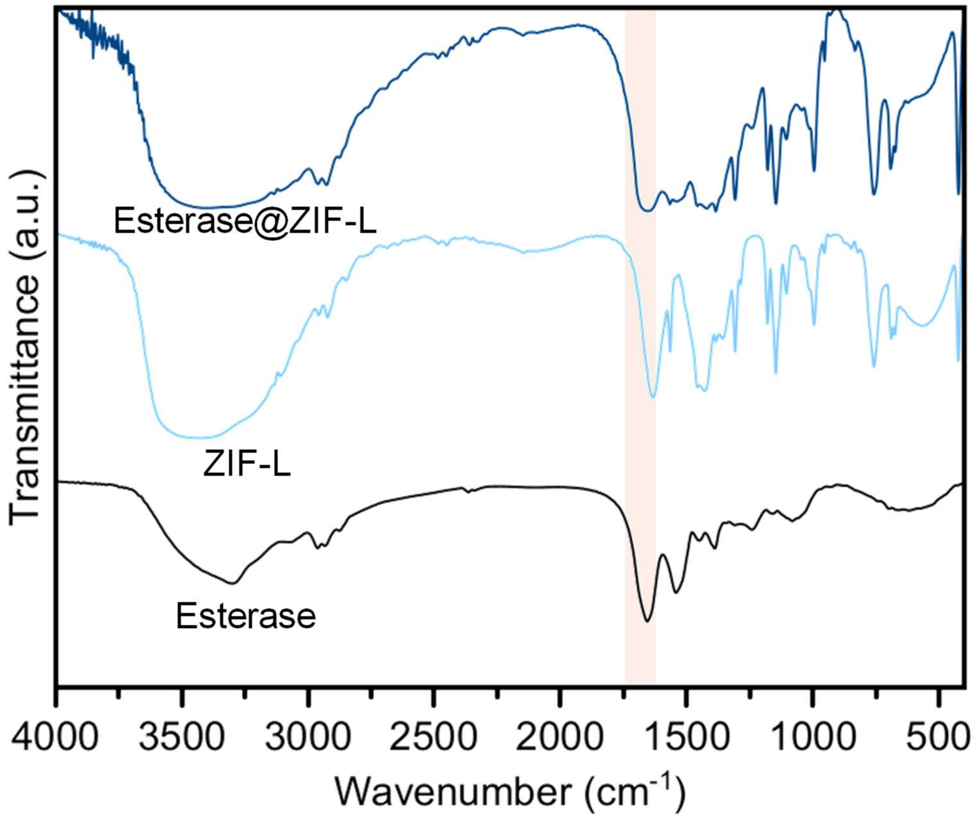

Figure S11. Fourier Transformed Infrared spectra (FTIR) of Esterase (black), ZIF-L (light blue) and Esterase@ZIF-L (dark blue) materials. The analysis of the FTIR data confirms the presence of the characteristic Amide I band (1700-1610 cm^{-1}) of the peptide backbone of Esterase highlighted in light orange.

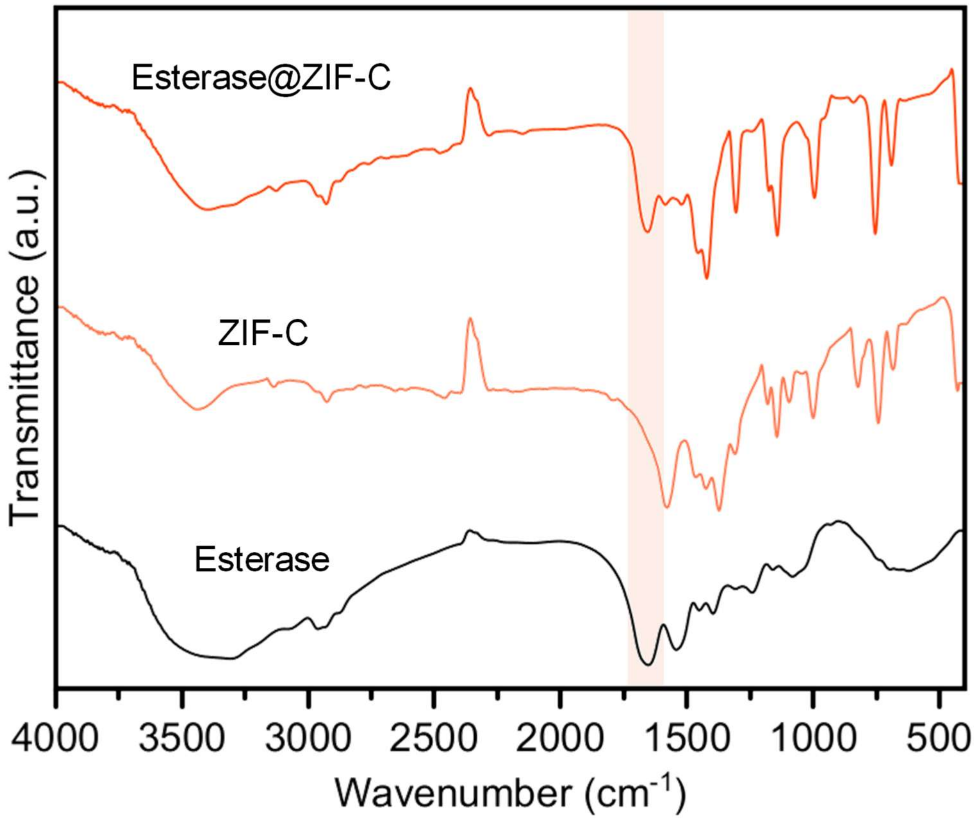

Figure S12. Fourier Transformed Infrared spectra (FTIR) of Esterase (black), ZIF-C (light red) and Esterase@ZIF-C (red) materials. The analysis of the FTIR data confirms the presence of the characteristic Amide I band (1700-1610 cm^{-1}) of the peptide backbone of Esterase highlighted in light orange.

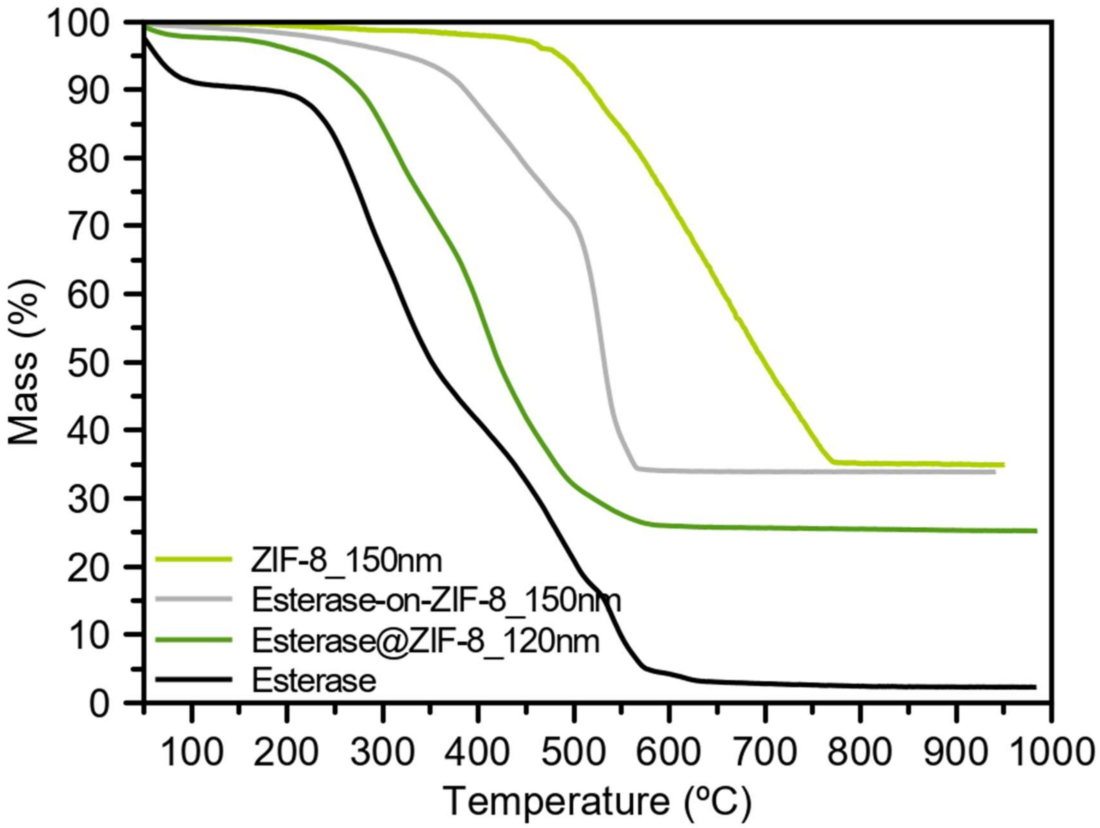

Figure S13. TGA profiles of ZIF-8_150nm (light green), Esterase-on-ZIF-8_150nm (grey), Esterase@ZIF-8_120nm (green) and Esterase (black) in a range from 30 to 950 °C under an oxidative atmosphere (air) with a ramp rate of 5 °C min^{-1}.

From thermogravimetric analyses of ZIF-8_150nm, Esterase-on-ZIF-8_150nm, Esterase@ZIF-8_120nm and Esterase we calculated the percentage of Esterase loading. The mass loss associated was taken into account as a function of their decomposition temperatures. Loading percentage of esterase by TG was calculated according to mass loss at esterase temperature of decomposition. Finally, we also compared Esterase loading in TGA with the Bradford assay obtaining very similar results (Table S12). The total weight percentage of Zn (wt_{Zn}) was calculated considering that the final solid residue (at 950 °C) corresponds to ZnO.

Table S12. Decomposition temperature and mass loss for ZIF-8_150nm, Esterase-on-ZIF-8_150nm, Esterase@ZIF-8_120nm and Esterase.

	$T_{Decomposition}$ (ºC)	$\Delta Mass_{150-950ºC}$ (mg)	Mass loss (%)	% Loading (TG)	% Loading (Brad.)	wt_{Zn} (%)
ZIF-8_150nm	625	8.0	65	-	-	29.5
Esterase-on-ZIF-8_150nm	522	7.9	65	4.0	2.6	27.3
Esterase@ ZIF-8_120nm	411	8.3	70	35	37	20.2
Esterase	280	8.6	88	-	-	-

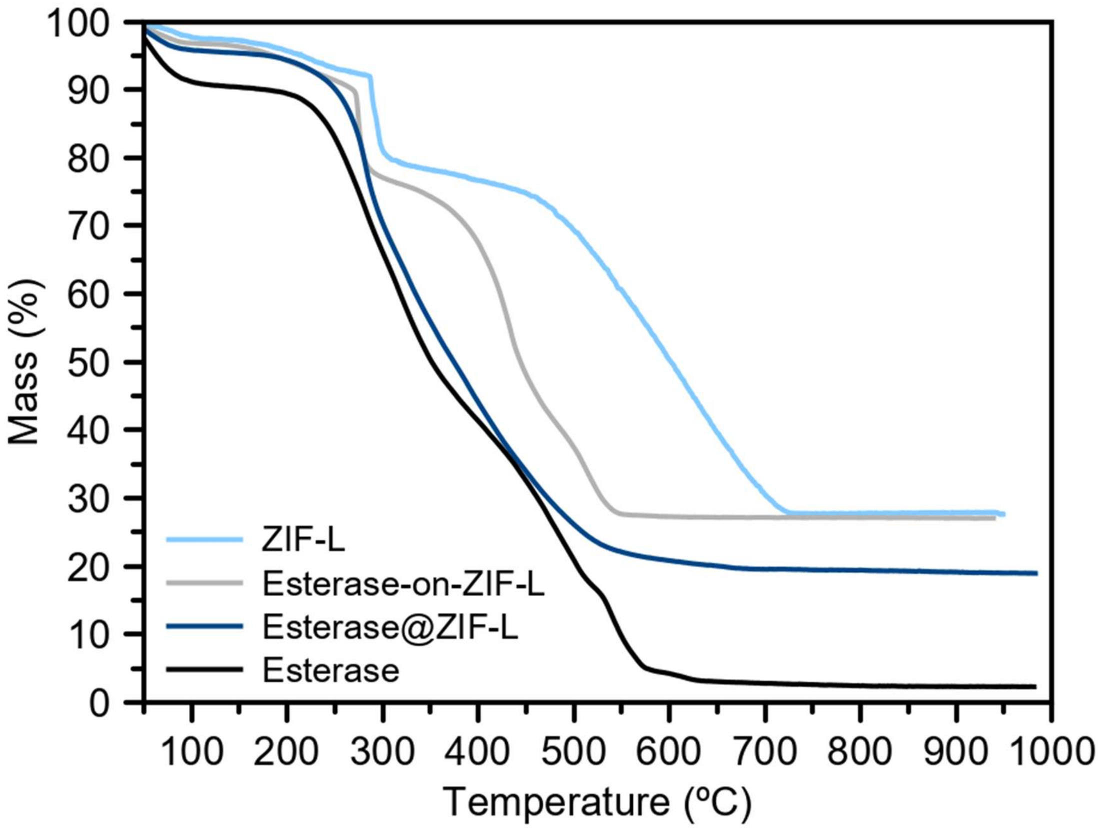

Figure S14. TGA profiles of ZIF-L (light blue), Esterase-on-ZIF-L (grey), Esterase@ZIF-L (dark blue) and Esterase (black) in a range from 30 to 950 °C under an oxidative atmosphere (air) with a ramp rate of 5 °C min^{-1}.

From thermogravimetric analyses of ZIF-L, Esterase-on-ZIF-L, Esterase@ZIF-L and Esterase we calculated the percentage of Esterase loading. The mass loss associated was taken into account as a function of their decomposition temperatures. Loading percentage of esterase by TG was calculated according to mass loss at esterase temperature of decomposition. Finally, we also compared Esterase loading in TGA with the Bradford assay obtaining very similar results (Table S13). The total weight percentage of Zn (wt_{Zn}) was calculated considering that the final solid residue (at 950 °C) corresponds to ZnO.

Table S13. Decomposition temperature and mass loss for ZIF-L, Esterase-on-ZIF-L, Esterase@ZIF-L and Esterase.

	$T_{Decomposition}$ (ºC)	$\Delta Mass_{150-950ºC}$ (mg)	Mass loss (%)	% Loading (TG)	% Loading (Brad.)	wt_{Zn} (%)
ZIF-L	550	6.3	70	-	-	24.7
Esterase -on-ZIF-L	406	7.3	69	5.7	4.4	25.3
Esterase @ZIF-L	330	7.0	76	36	37	15.2
Esterase	280	8.6	88	-	-	-

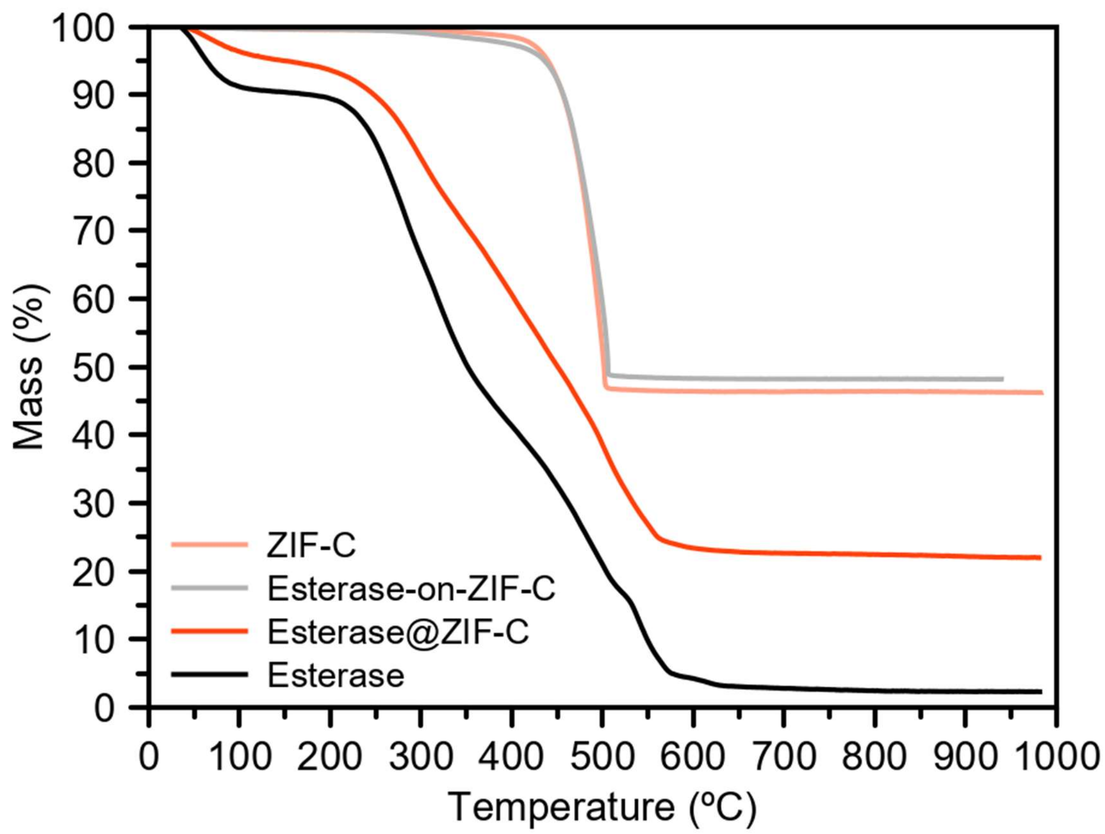

Figure S15. TGA profiles of ZIF-C (light red), Esterase-on-ZIF-C (grey), Esterase@ZIF-C (red) and Esterase (black) in a range from 30 to 950 °C under an oxidative atmosphere (air) with a ramp rate of 5 °C min^{-1}.

From thermogravimetric analyses of ZIF-C, Esterase-on-ZIF-C, Esterase@ZIF-C and Esterase we calculated the percentage of Esterase loading. The mass loss associated was taken into account as a function of their decomposition temperatures. Loading percentage of esterase by TG was calculated according to mass loss at esterase temperature of decomposition. Finally, we also compared Esterase loading in TGA with the Bradford assay obtaining very similar results (Table S14). The total weight percentage of Zn (wt_{Zn}) was calculated considering that the final solid residue (at 950 °C) corresponds to ZnO.

Table S14. Decomposition temperature and mass loss for ZIF-C, Esterase-on-ZIF-C, Esterase@ZIF-C and Esterase.

	$T_{Decomposition}$ (ºC)	$\Delta Mass_{150-950ºC}$ (mg)	Mass loss (%)	% Loading (TG)	% Loading (Brad.)	wt_{Zn} (%)
ZIF-C	490	5.2	54	-	-	37.2
Esterase-on-ZIF-C	488	5.3	51	1.9	2.0	38.8
Esterase @ZIF-C	370	7.5	73	41	47	17.7
Esterase	280	8.6	88	-	-	-

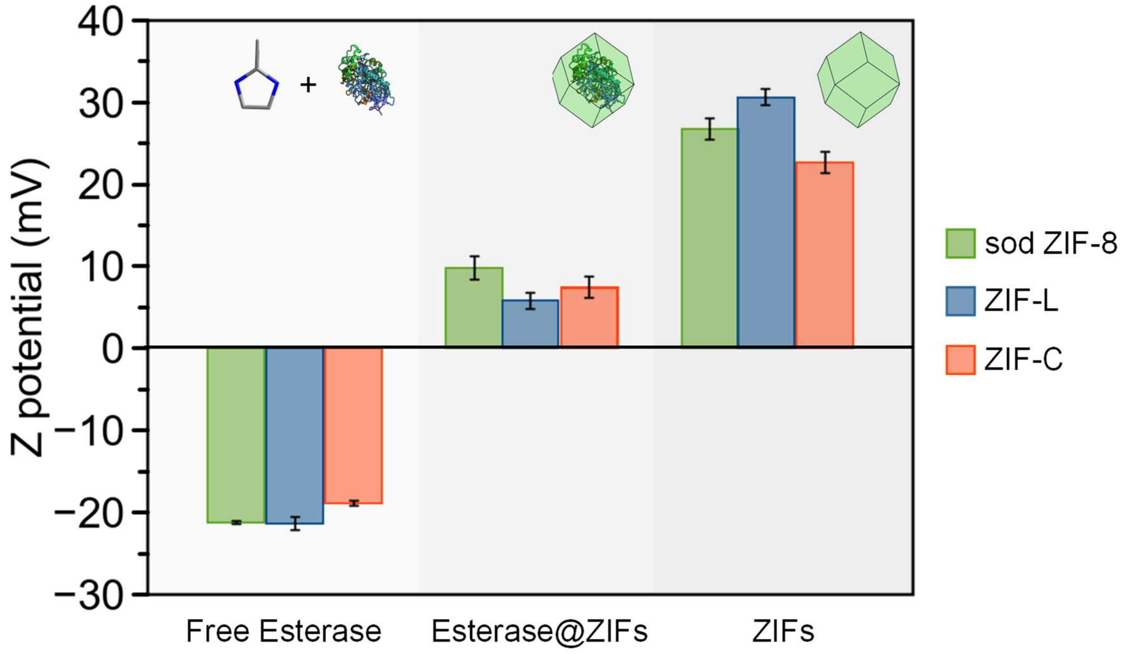

Figure S16. Z potential values of free Esterase in precursor ligand solutions (left), Esterase@ZIFs biomineralized (middle) and Zeolitic Imidazolate Frameworks alone (right).

Since Esterase has an isoelectric point of 5.0[10], in an aqueous solution (pH ≈ 7) it possesses a net negative charge. When Esterase is added to a basic 2-methylimidazole ligand solution (see synthetic section S2.1.1.), the surface charge of this protein becomes even more negative (green, blue and red bars for the protein + ZIF-8_150nm, ZIF-L and ZIF-C ligand solutions, respectively). After the addition of the metal solution and biomineralization of the ZIFs, the net charge of the particles in all cases is positive due to the Zn ions in their crystalline structural surface. We can appreciate that ZIFs mineralized without Esterase have a more positive Z potential value because of the absence of this enzyme.

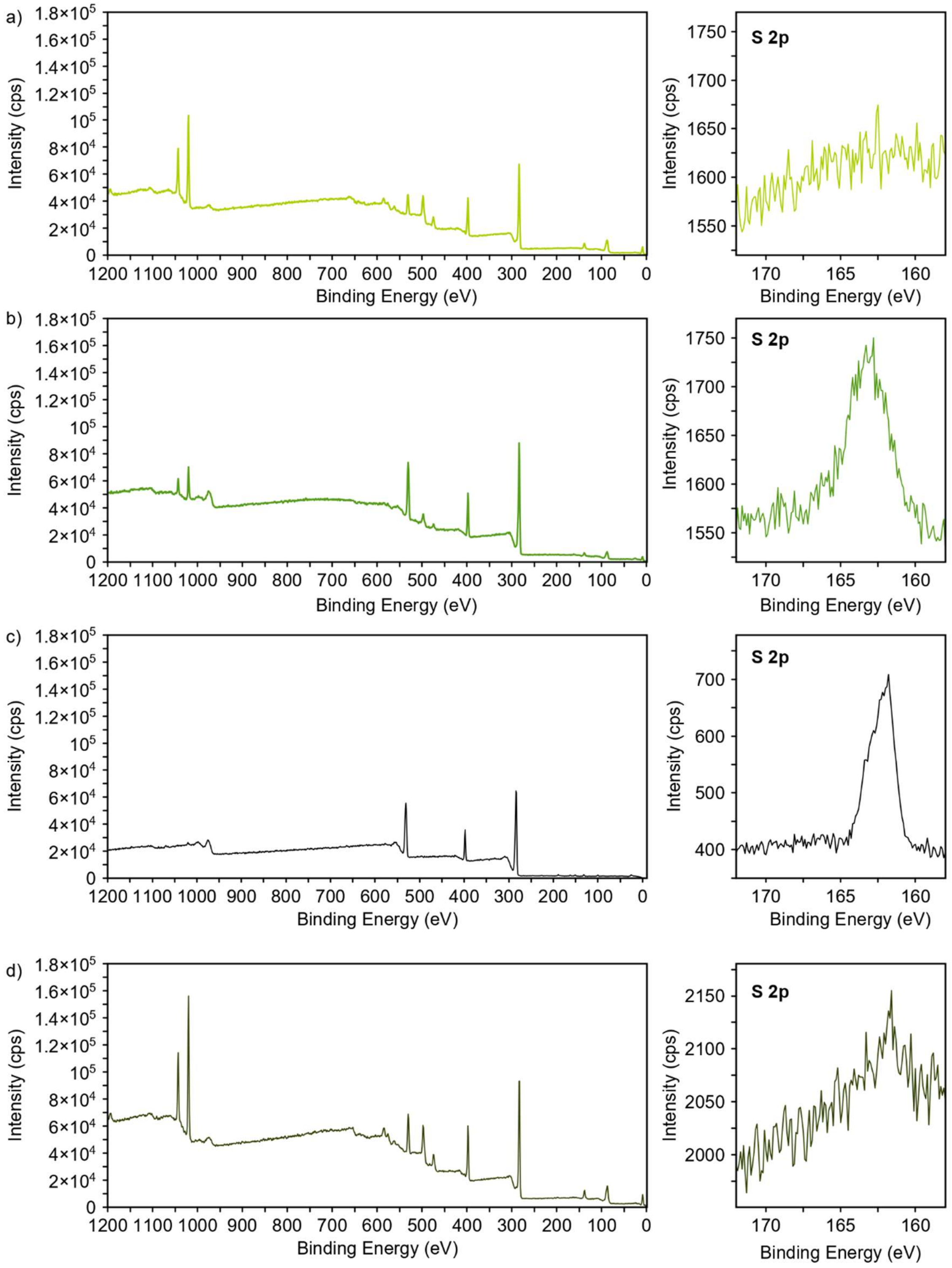

Figure S17. Experimental X-ray Photoelectron Spectroscopy (XPS) analysis together with the peak associated with the broadened S 2p signal of (a) ZIF-8_150nm, (b) Esterase@ZIF-8_120nm, (c) esterase and (d) ZIF-8_150nm+esterase physical mixture.

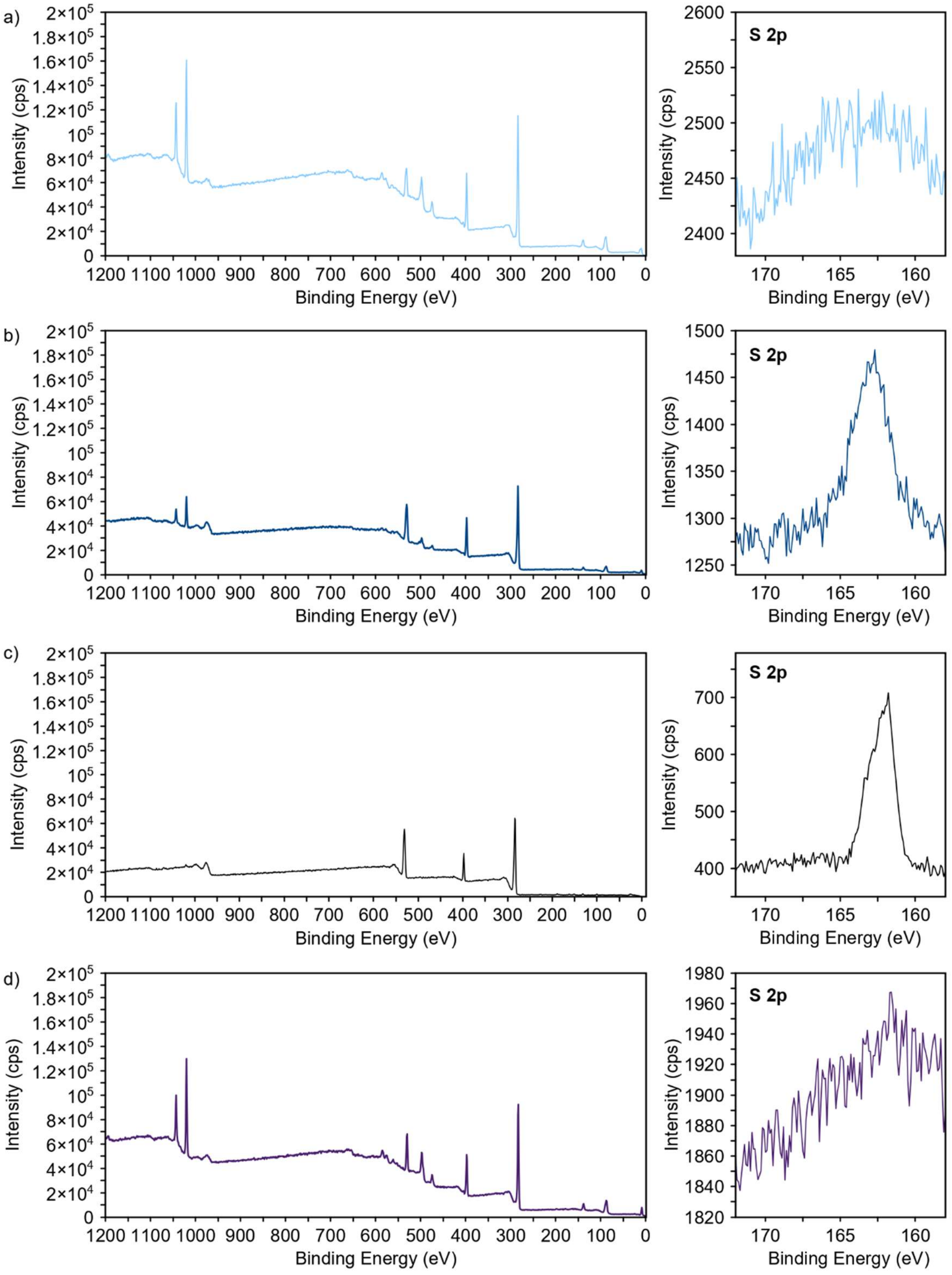

Figure S18. Experimental X-ray Photoelectron Spectroscopy (XPS) analysis together with the peak associated with the broadened S 2p signal of (a) ZIF-L, (b) Esterase@ZIF-L, (c) esterase and (d) ZIF-L+esterase physical mixture.

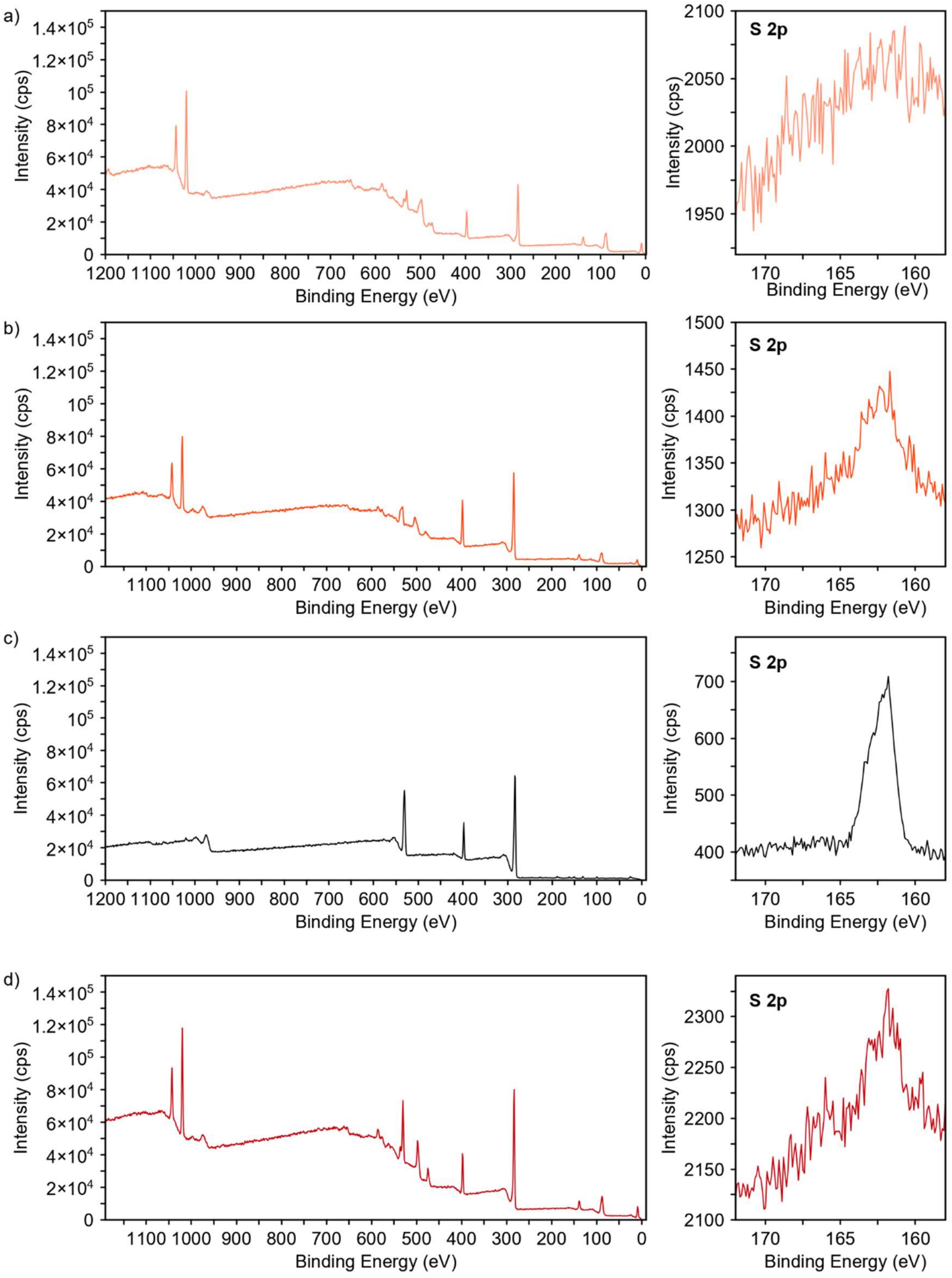

Figure S19. Experimental X-ray Photoelectron Spectroscopy (XPS) analysis together with the peak associated with the broadened S 2p signal of (a) ZIF-C, (b) Esterase@ZIF-C, (c) esterase and (d) ZIF-C+esterase physical mixture.

Table S15. Quantification in wt% of the different elements from XPS analysis (10 nm depth).

Sample	C	O	N	Zn	S
ZIF-8_150nm	50.2	6.72	16.06	27.02	0
Esterase@ZIF-8_120nm	60.3	17.11	13.79	8.3	0.48
Esterase+ZIF-8_150nm Physical Mixture	49.56	8.99	14.75	26.33	0.37
ZIF-L	50.76	8.97	14.76	25.51	0
Esterase@ZIF-L	57.41	17.34	15.83	8.84	0.58
Esterase+ZIF-L Physical Mixture	52.05	10.01	15.15	22.57	0.22
ZIF-C	42.3	9.93	10.74	37.03	0
Esterase@ZIF-C	50.74	10.64	17.15	20.76	0.71
Esterase+ZIF-C Physical Mixture	50.66	15.92	10.98	21.58	0.52
Esterase	62.92	21.8	11.27	0.92	0.45

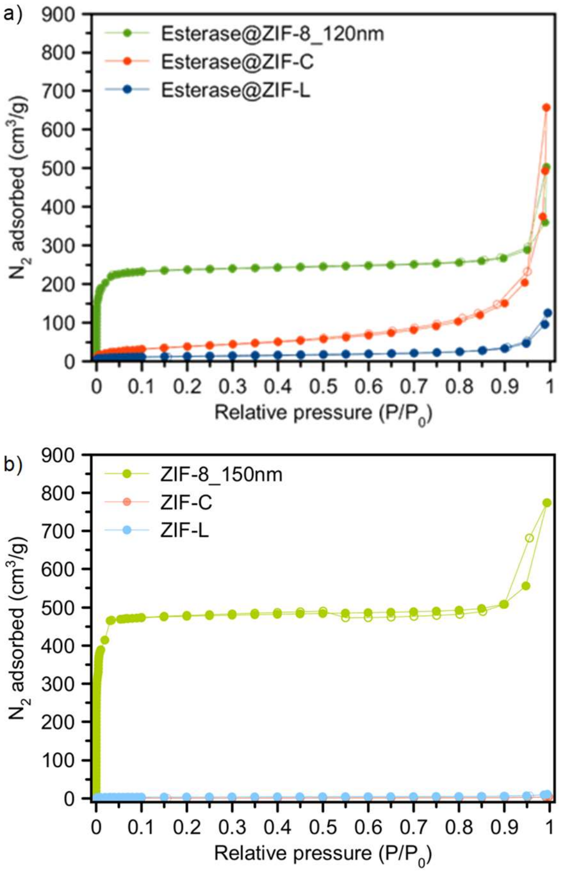

Figure S20. Nitrogen adsorption isotherms at 77 K of (a) Esterase@ZIF biocomposites and (b) ZIFs alone. Each sample was activated at 120 ºC for 12 h under dynamic vacuum. The adsorption and desorption process of each N_2 isotherm are represented by filled and empty dots, respectively.

Table S16. BET surface area of Esterase@ZIF biocomposites in m^2 per gram of total material and per gram of MOF and their comparison to the ZIF original phases.

	S_{BET} (m^2/g)	S_{BET}(m^2/g) normalized to MOF mass	S_{BET} (m^2/g_{MOF})
Esterase@ZIF-8_120nm	950	1460	-
Esterase@ZIF-C	120	240	-
Esterase@ZIF-L	45	120	-
ZIF-8_150nm	-	-	1890
ZIF-C	-	-	2
ZIF-L	-	-	11

BET values obtained are referred to the Biocomposites taking into account both the ZIF and Esterase mass (column 1). We have carried out the calculation of the BET surface area related to the amount of ZIF present in the total material (column 2) and comparison to ZIF materials alone (column 3). To do this, we have considered the percentage of ZIF of each biocomposite (see Table S8).

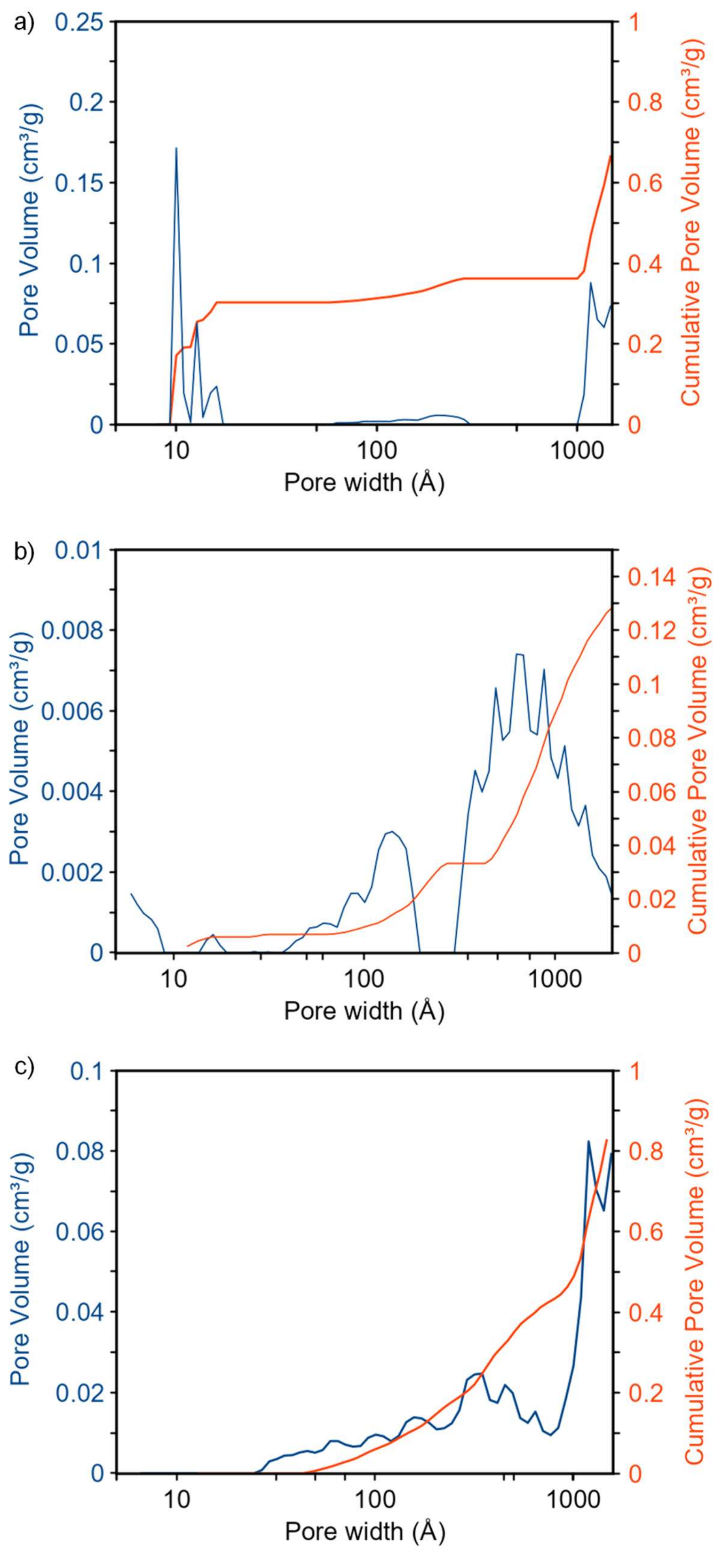

Figure S21. Pore volume distribution and cumulative pore volume calculated by Density Functional Theory from the experimental nitrogen adsorption isotherm curves for (a) Esterase@ZIF-8_120nm, (b) Esterase@ZIF-L and (c) Esterase@ZIF-C biocomposites.

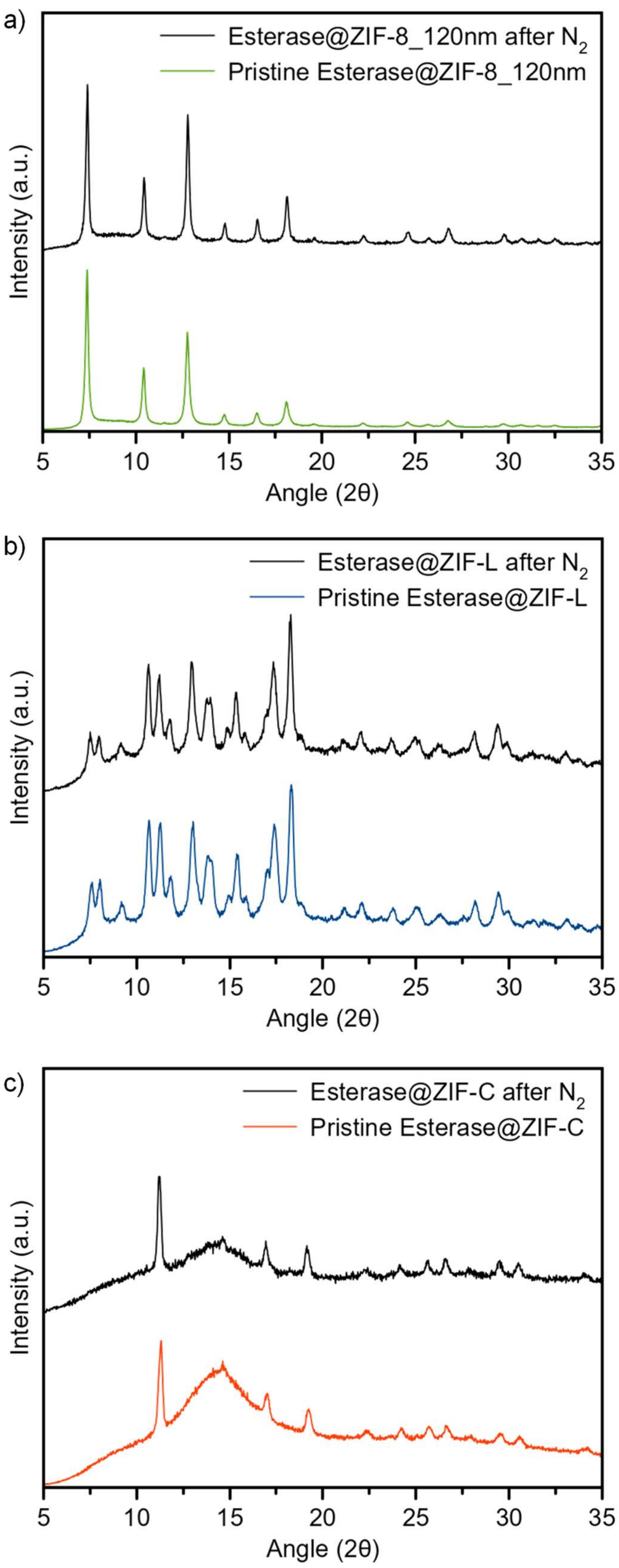

Figure S22. Powder X-Ray Diffraction patterns of (a) Esterase@ZIF-8_120nm, (b) Esterase@ZIF-L and (c) Esterase@ZIF-C biocomposites after nitrogen adsorption isotherm protocol. The comparison between each material before and after the activation protocol indicates that crystal phase is well maintained for each Esterase@ZIF.

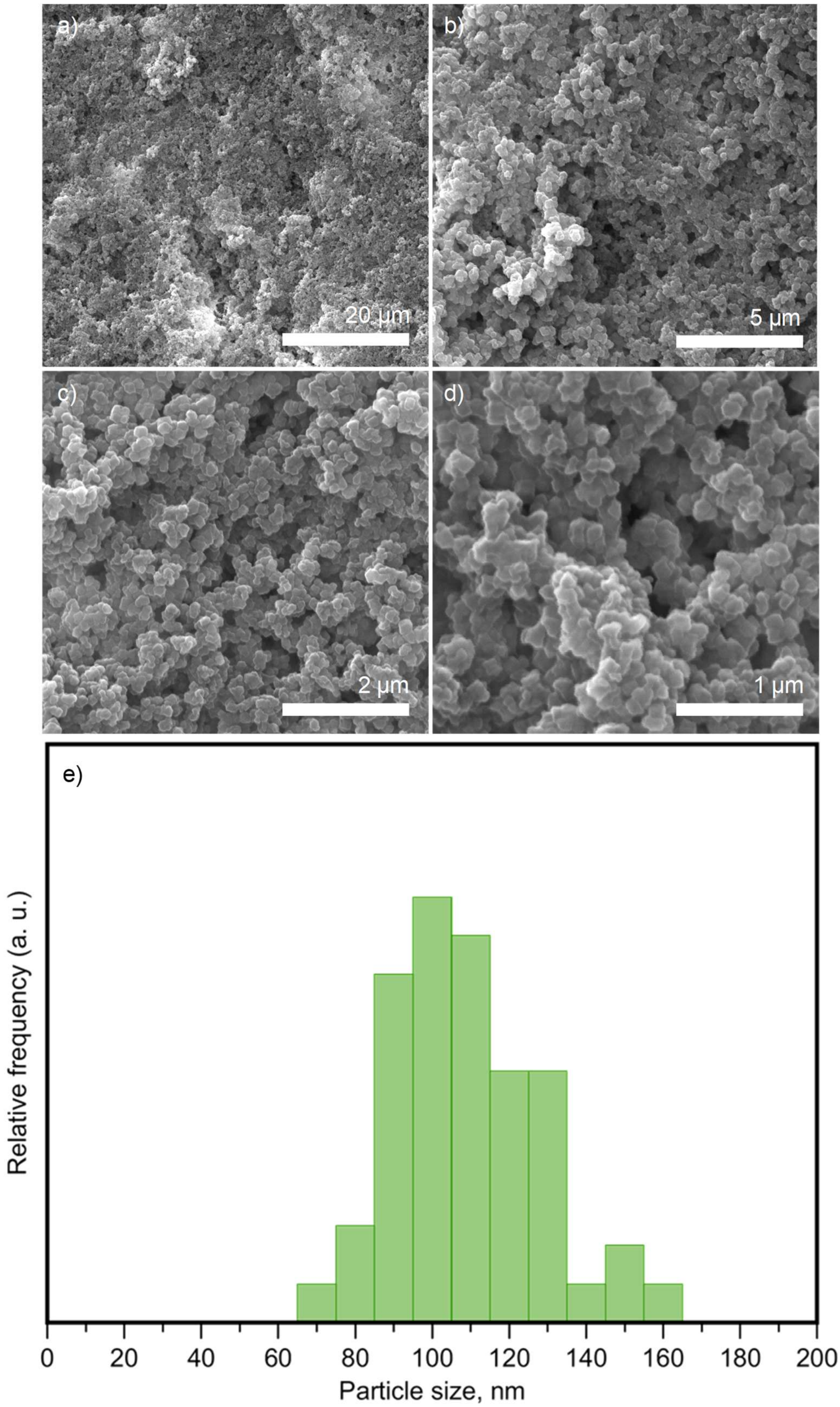

Figure S23. Scanning Electron Microscopy (SEM) images of Esterase@ZIF-8_120nm at different magnifications: a) 20 µm, b) 5 µm, c) 2 µm and d) 1 µm. (e) Particle size distribution of Esterase@ZIF-8_120nm nanoparticles was calculated based on SEM images.

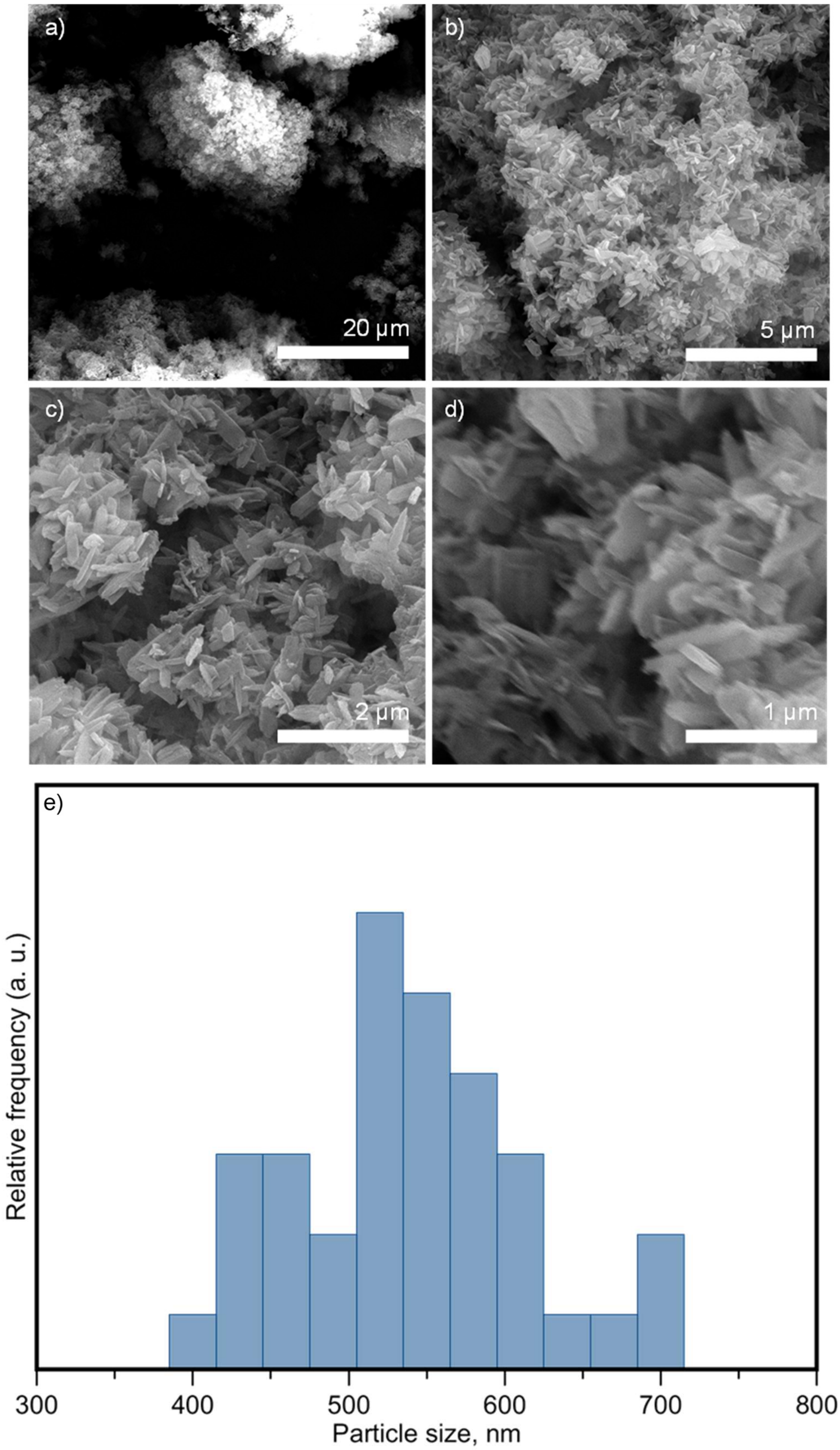

Figure S24. Scanning Electron Microscopy (SEM) images of Esterase@ZIF-L at different magnifications: a) 20 µm, b) 5 µm, c) 2 µm and d) 1 µm. (e) Particle size distribution of Esterase@ZIF-L nanoparticles was calculated based on SEM images.

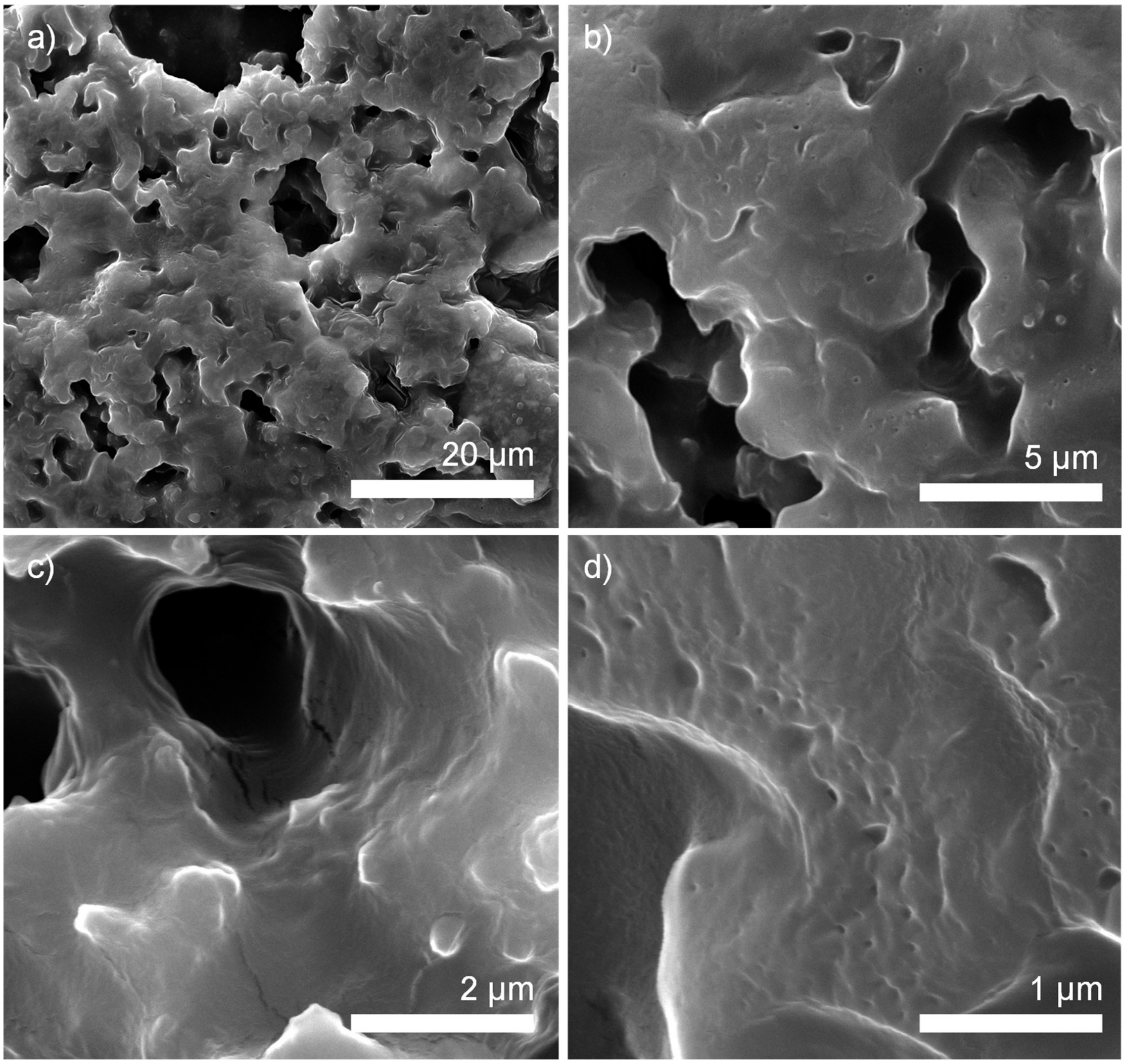

Figure S25. Scanning Electron Microscopy (SEM) images of Esterase@ZIF-C at different magnifications: a) 20 μm, b) 5 μm, c) 2 μm and d) 1 μm.

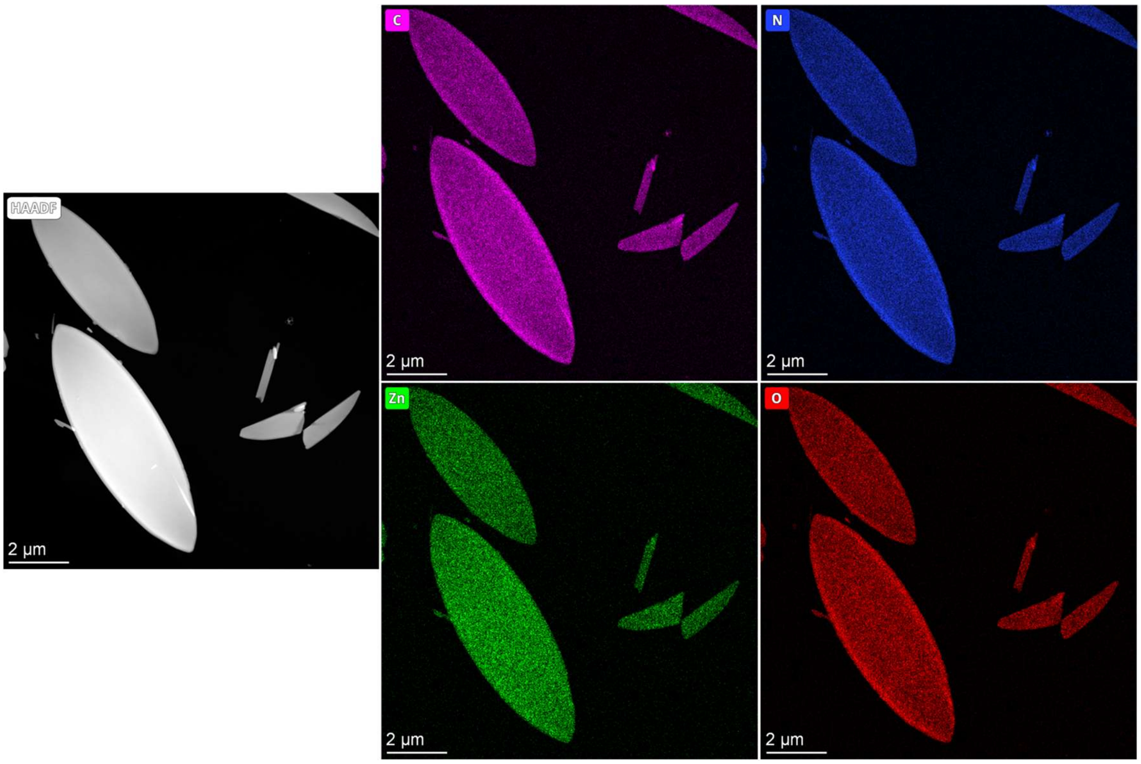

Figure S26. Transmission Electron Microscopy-Energy Dispersive X-ray spectroscopy (TEM-EDX) elemental maps of ZIF-L microparticles.

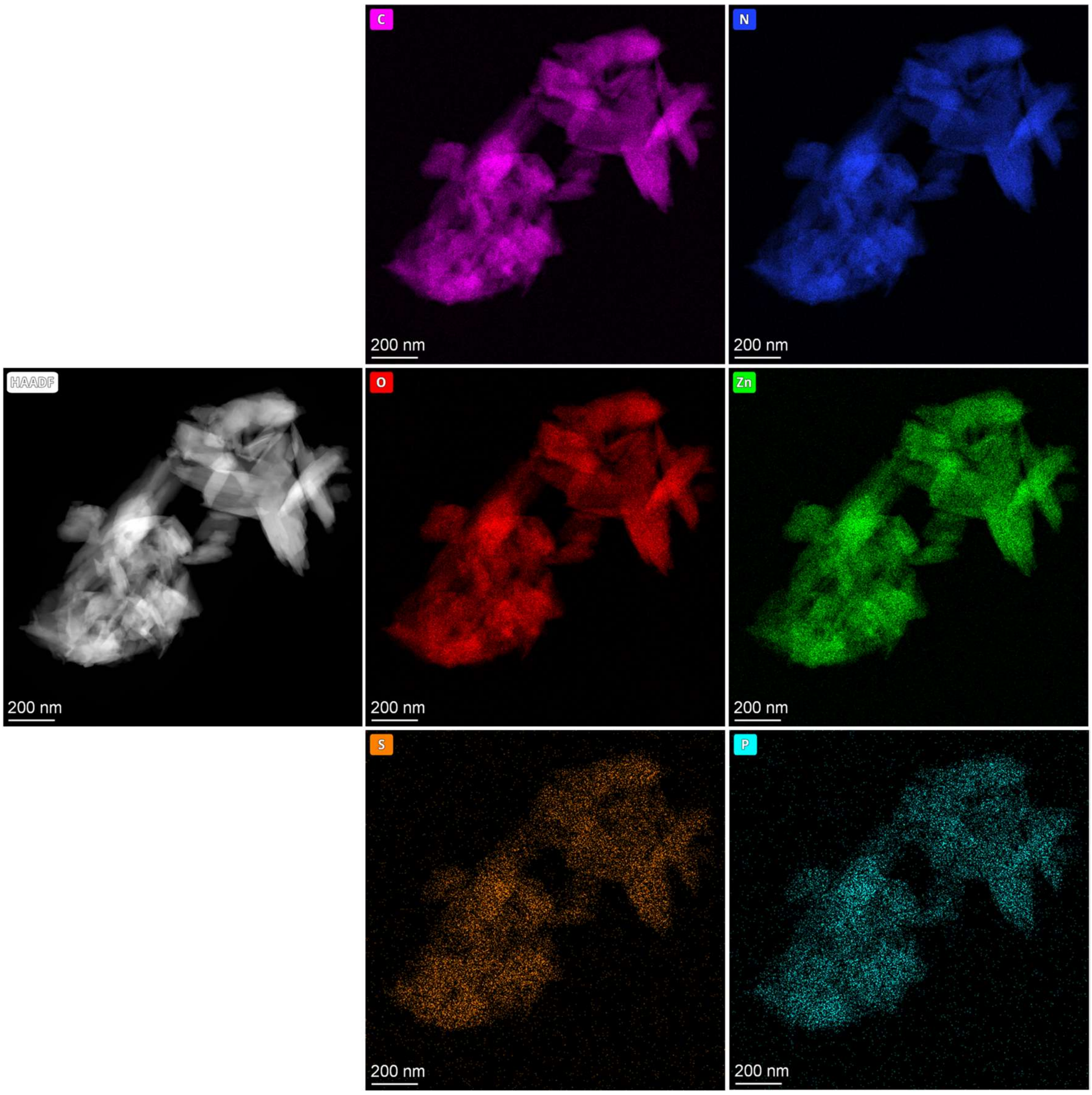

Figure S27. Transmission Electron Microscopy-Energy Dispersive X-ray spectroscopy (TEM-EDX) elemental maps of Esterase@ZIF-L nanoparticles.

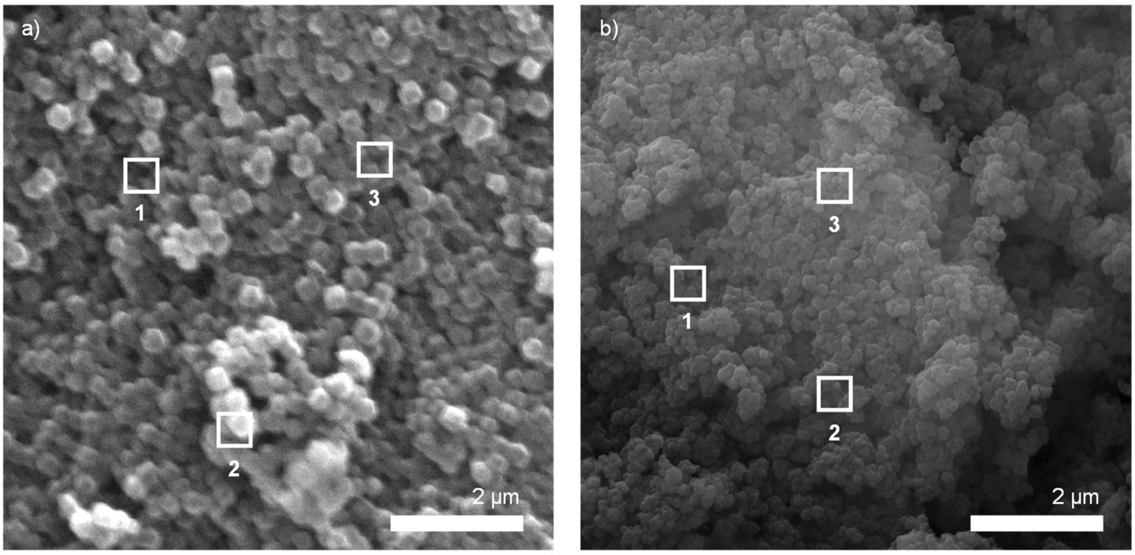

Area	N	O	P	S	Zn
1	50.83	34.56	0.00	0.00	14.61
2	39.00	44.56	0.00	0.06	16.38
3	48.49	33.89	0.12	0.10	17.40

Area	N	O	P	S	Zn
1	49.43	38.15	0.26	0.46	11.71
2	60.62	21.22	0.08	0.75	17.34
3	57.63	23.00	0.39	0.56	18.42

Figure S28. Scanning Electron Microscopy-Energy Dispersive X-ray spectroscopy (SEM-EDX) elemental analysis of (a) ZIF-8_150nm and (b) Esterase@ZIF-8_120nm.

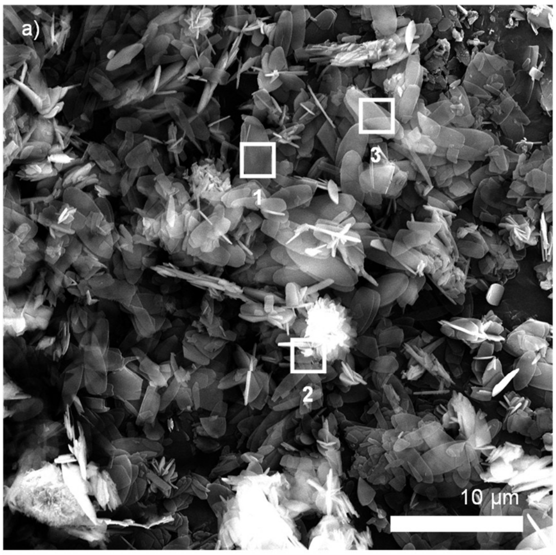

Area	N	O	P	S	Zn
1	40.57	48.56	0.00	0.09	10.78
2	44.21	44.13	0.00	0.03	11.63
3	36.17	52.42	0.03	0.02	10.96

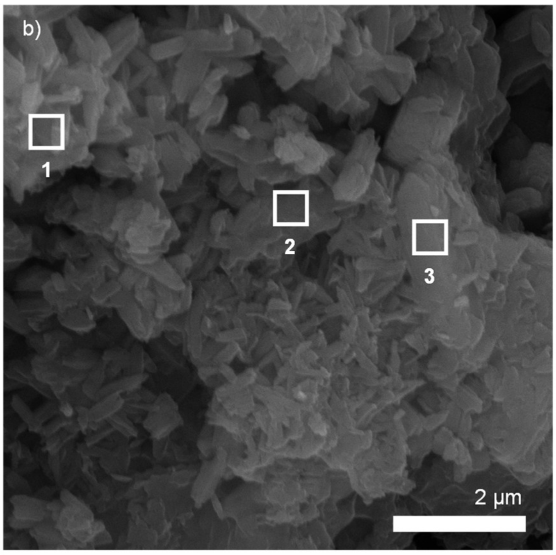

Area	N	O	P	S	Zn
1	52.61	34.98	0.34	1.07	11.01
2	54.46	25.15	0.51	0.33	19.55
3	59.56	29.41	0.18	0.38	10.47

Figure S29. Scanning Electron Microscopy-Energy Dispersive X-ray spectroscopy (SEM-EDX) elemental analysis of (a) ZIF-L and (b) Esterase@ZIF-L.

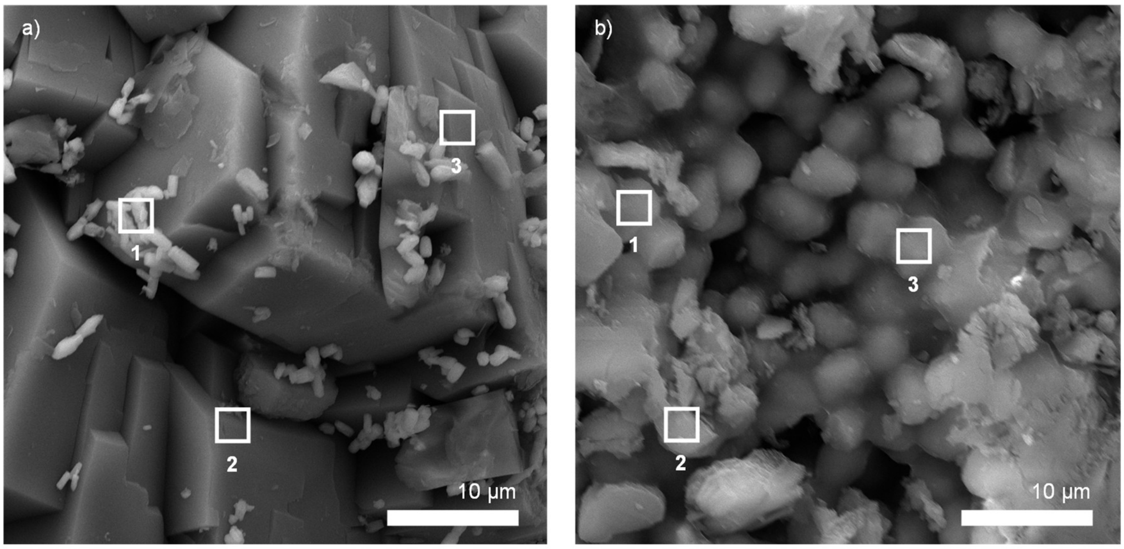

Area	N	O	P	S	Zn
1	12.98	14.27	0.12	0.00	72.63
2	26.44	31.08	0.03	0.21	42.23
3	12.31	14.75	0.00	0.01	72.92

Area	N	O	P	S	Zn
1	38.47	38.43	0.49	0.57	22.05
2	37.77	49.12	0.08	0.22	12.81
3	41.43	38.33	0.30	1.03	18.91

Figure S30. Scanning Electron Microscopy-Energy Dispersive X-ray spectroscopy (SEM-EDX) elemental analysis of (a) ZIF-C and (b) Esterase@ZIF-C.

S3.2. Characterization of BSA@ZIF biocomposites

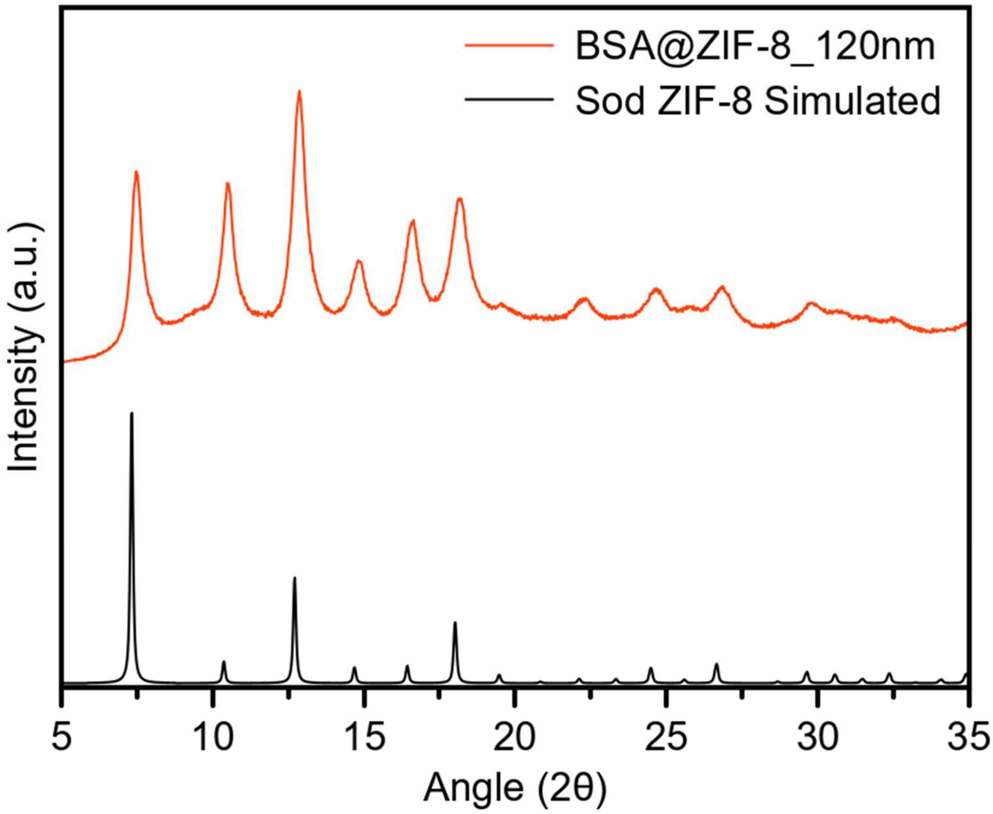

Figure S31. Powder X-Ray Diffraction pattern of BSA@ZIF-8_120nm biocomposite (experimental, red; sod ZIF-8 calculated, black).

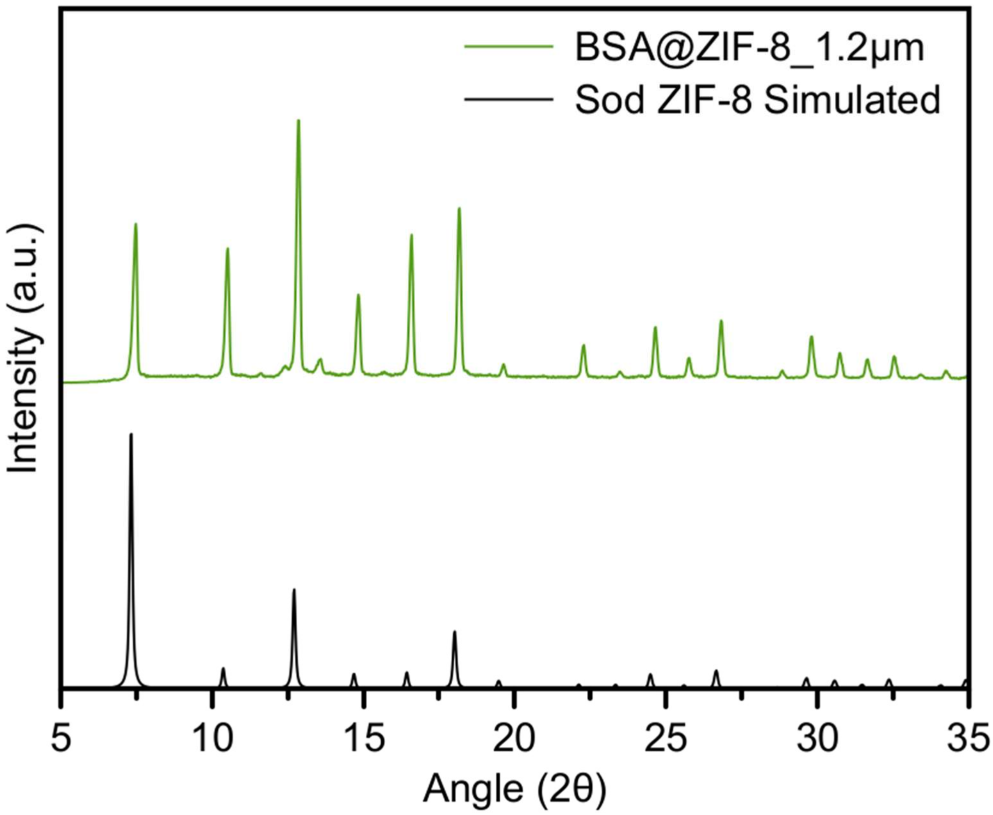

Figure S32. Powder X-Ray Diffraction pattern of BSA@ZIF-8_1.2µm biocomposite (experimental, green; sod ZIF-8 calculated, black).

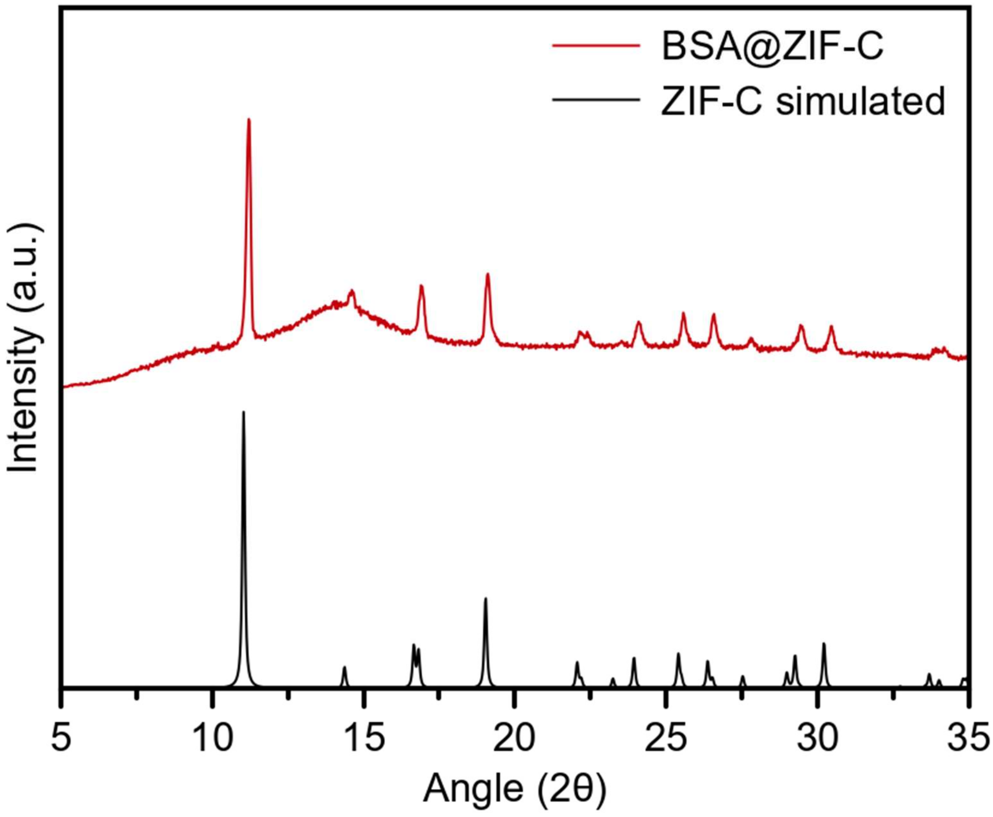

Figure S33. Powder X-Ray Diffraction pattern of BSA@ZIF-C biocomposite (experimental, dark red; ZIF-C calculated, black).

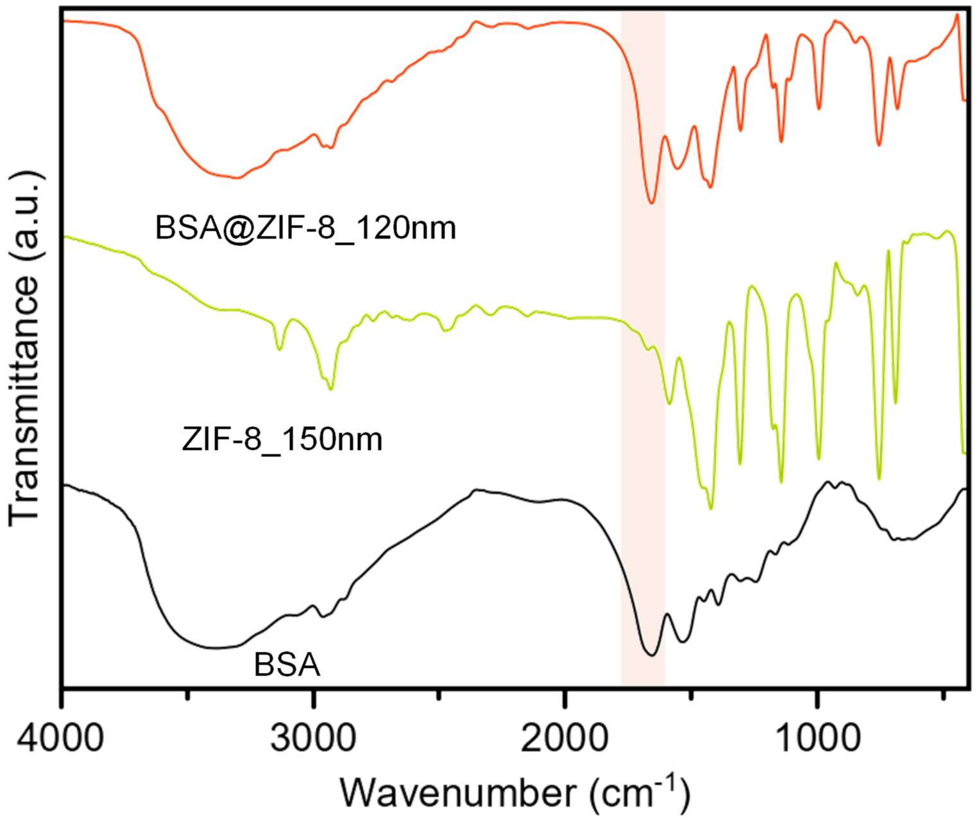

Figure S34. FTIR of BSA (black), ZIF-8_150nm (light green) and BSA@ZIF-8_120nm (red) materials. The analysis of the FTIR data confirms the presence of the characteristic Amide I band (1700-1610 cm^{-1}) of the peptide backbone of BSA highlighted in light orange.

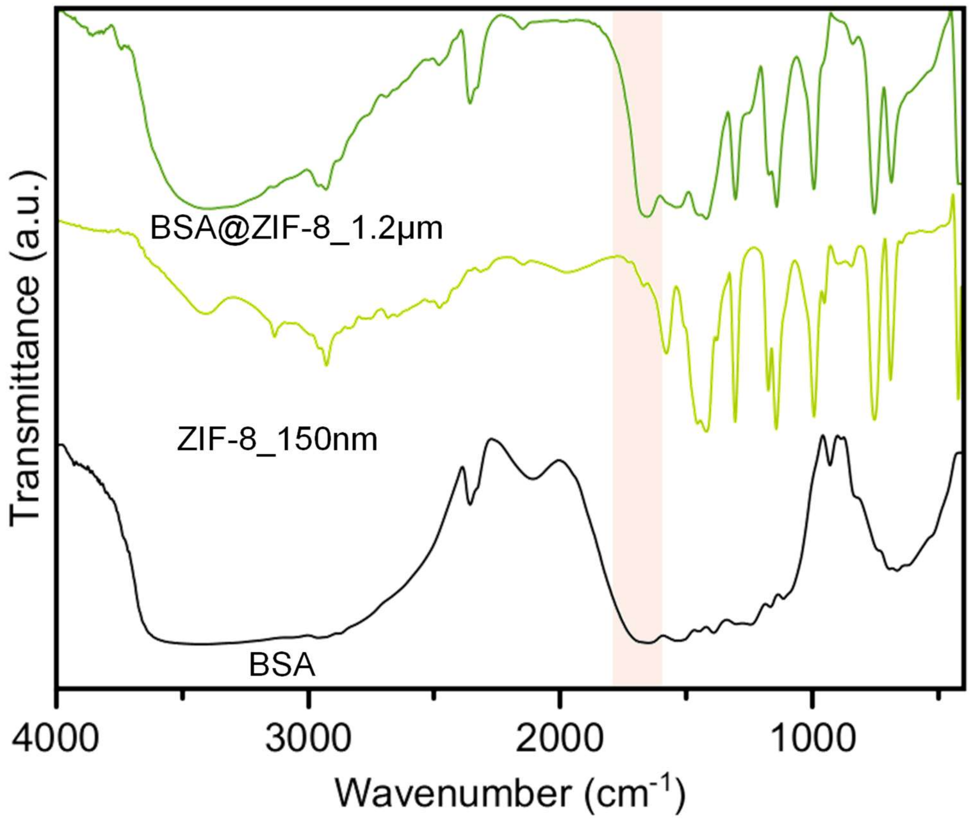

Figure S35. FTIR of BSA (black), ZIF-8_150nm (light green) and BSA@ZIF-8_1.2μm (dark green) materials. The analysis of the FTIR data confirms the presence of the characteristic Amide I band (1700-1610 cm^{-1}) of the peptide backbone of BSA highlighted in light orange.

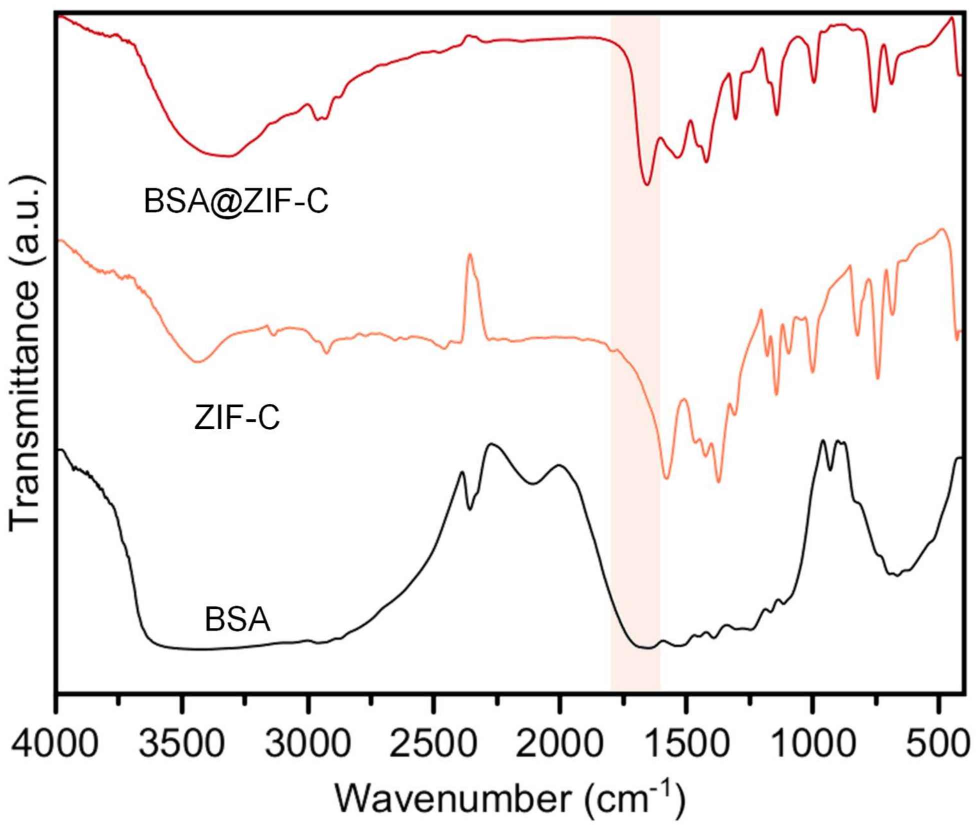

Figure S36. FTIR of BSA (black), ZIF-C (light red) and BSA@ZIF-C (dark red) materials. The analysis of the FTIR data confirms the presence of the characteristic Amide I band (1700-1610 cm^{-1}) of the peptide backbone of BSA highlighted in light orange.

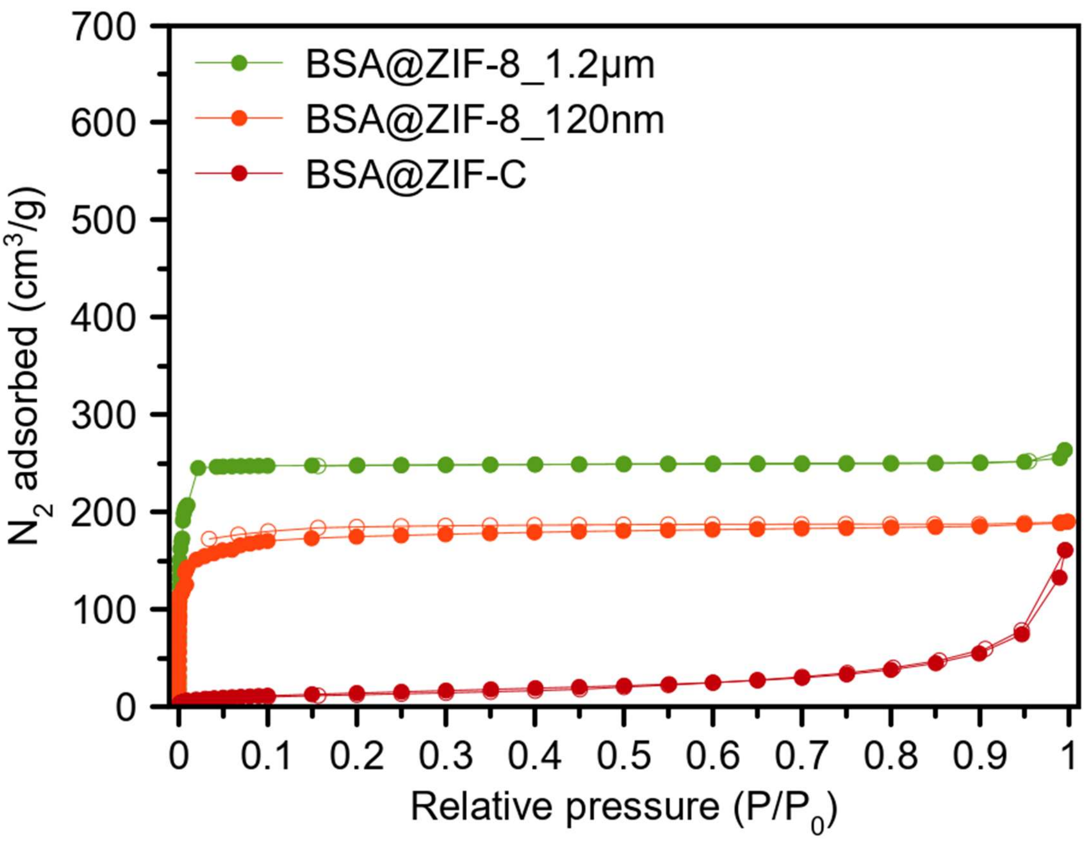

Figure S37. Nitrogen adsorption isotherm at 77 K of BSA@ZIF-8_1.2µm (green), BSA@ZIF-8_120nm (light red) and Esterase@ZIF-C (dark red) biocomposites. Each sample was activated at 120 ºC for 12 h under dynamic vacuum. The adsorption and desorption process of each N_2 isotherm are represented by filled and empty dots, respectively.

Table S17. BET surface area of BSA@ZIF biocomposites in m^2 per gram of total material and per gram of MOF and their comparison to the ZIF original phases.

	S_{BET} (m^2/g)	S_{BET}(m^2/g) normalized to MOF mass	S_{BET} (m^2/g_{MOF})
BSA@ZIF-8_1.2µm	1031	1610	-
BSA@ZIF-C	42	63	-
BSA@ZIF-8_120nm	680	1214	-
ZIF-8_150nm	-	-	1890
ZIF-C	-	-	2

BET values obtained are referred to the Biocomposites taking into account both the ZIF and BSA mass (column 1). We have carried out the calculation of the BET surface area related to the amount of ZIF present in the total material (column 2) and comparison to ZIF materials alone (column 3). To do this, we have considered the percentage of ZIF of each biocomposite (see Table S8).

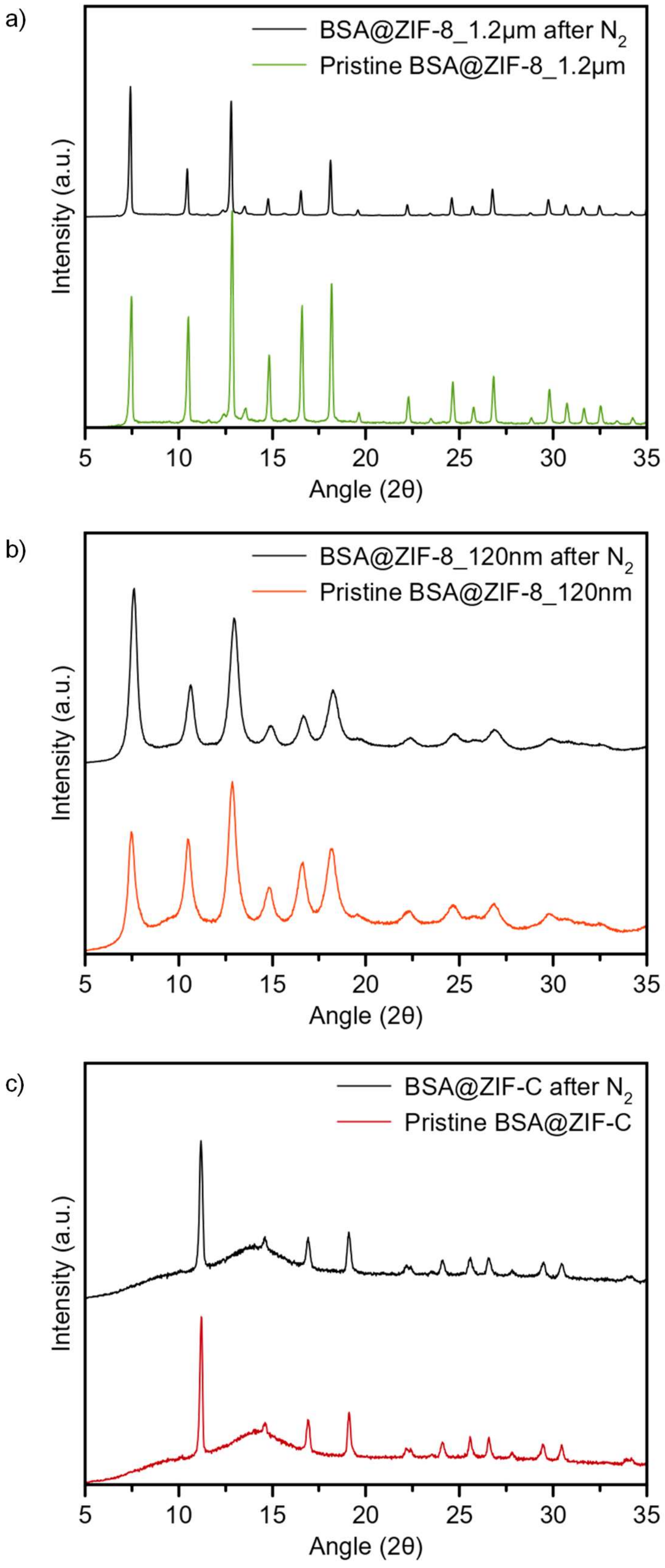

Figure S38. Powder X-Ray Diffraction patterns of (a) BSA@ZIF-8_1.2μm and (b) BSA@ZIF-C biocomposites after nitrogen adsorption isotherm protocol. The comparison between each material before and after the activation protocol indicates that crystal phase is well maintained.

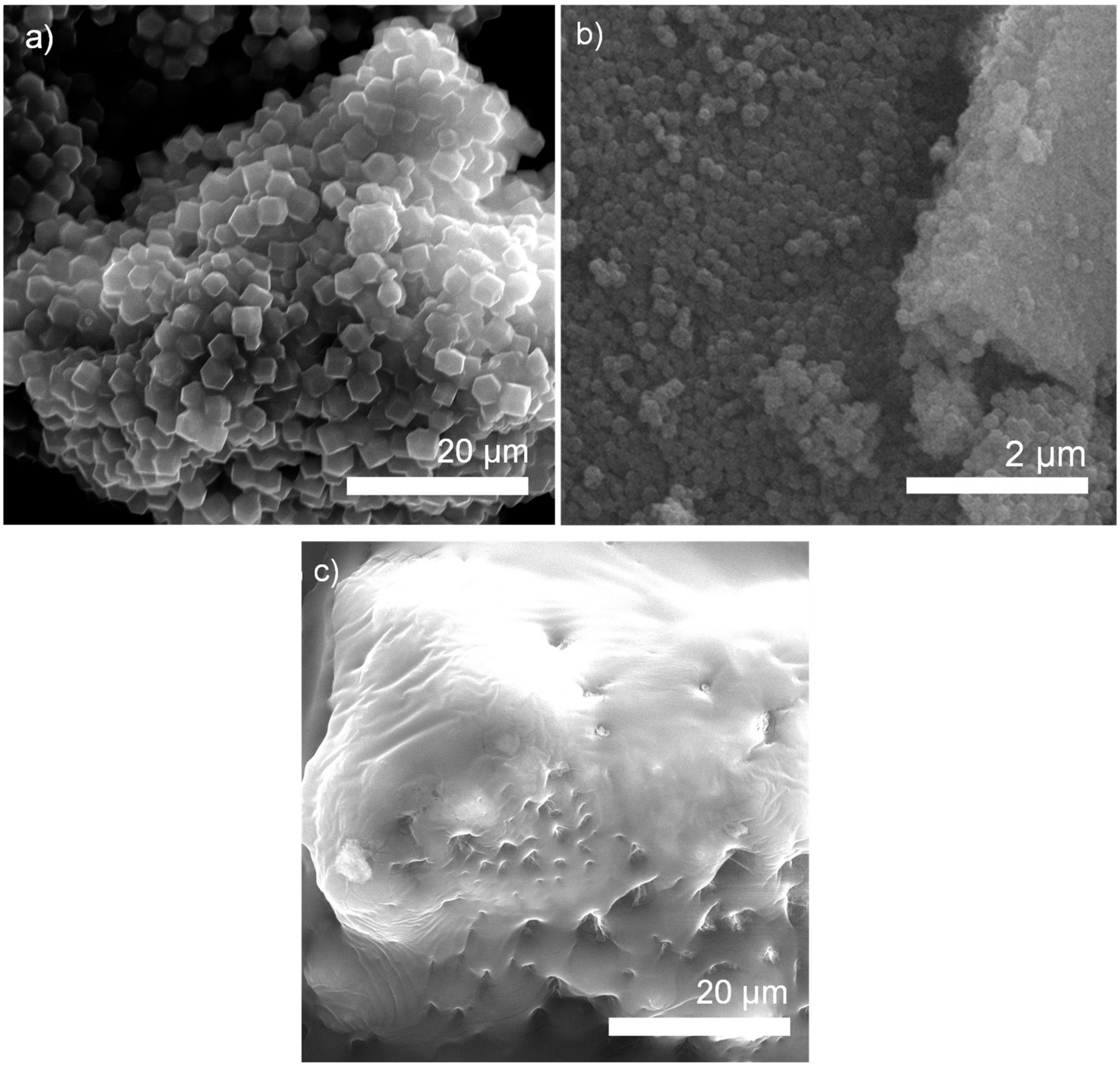

Figure S39. Scanning Electron Microscopy (SEM) images of (a) BSA@ZIF-8_1.2µm, (b) BSA@ZIF-8_120nm and (c) BSA@ZIF-C.

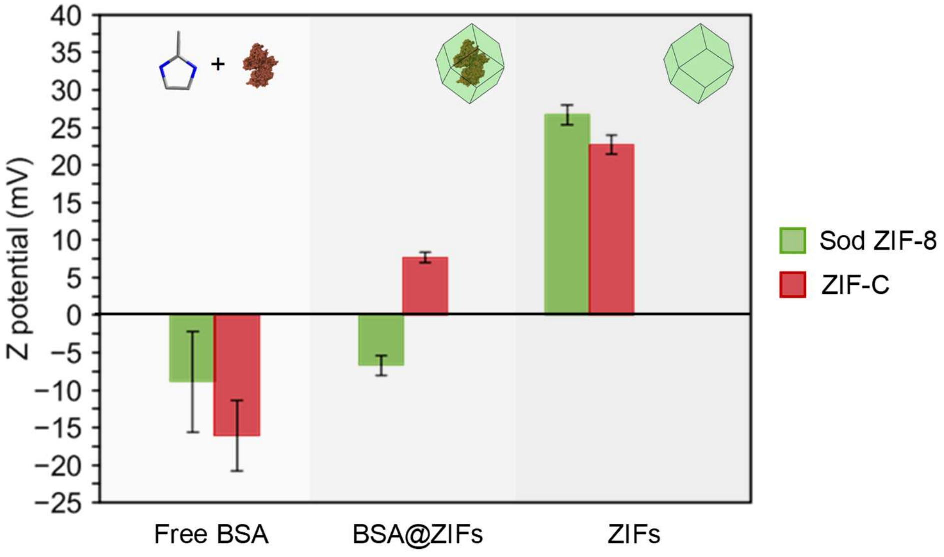

Figure S40. Z potential values of free BSA in precursor ligand solution (left), BSA@ZIF biomineralized (middle) and Zeolitic Imidazolate Frameworks alone (right).

Since BSA has an isoelectric point of 4.7[11], in an aqueous solution (pH ≈ 7) it possesses a net negative charge. When BSA is added to a basic 2-methylimidazole ligand solution (see synthetic section S2.1.5.), the surface charge of this protein becomes even more negative (green and red bars for the BSA + ZIF-8_150nm and BSA+ZIF-C ligand solutions, respectively). After the addition of the metal solution and biomineralization of the ZIFs, the net charge of the particles changes due to the Zn ions in their crystalline structural surface (BSA@ZIF-8_120nm, green; and BSA@ZIF-C, dark red).

S3.2. Synergistic interplay of esterase and ZIF on biocomposite function

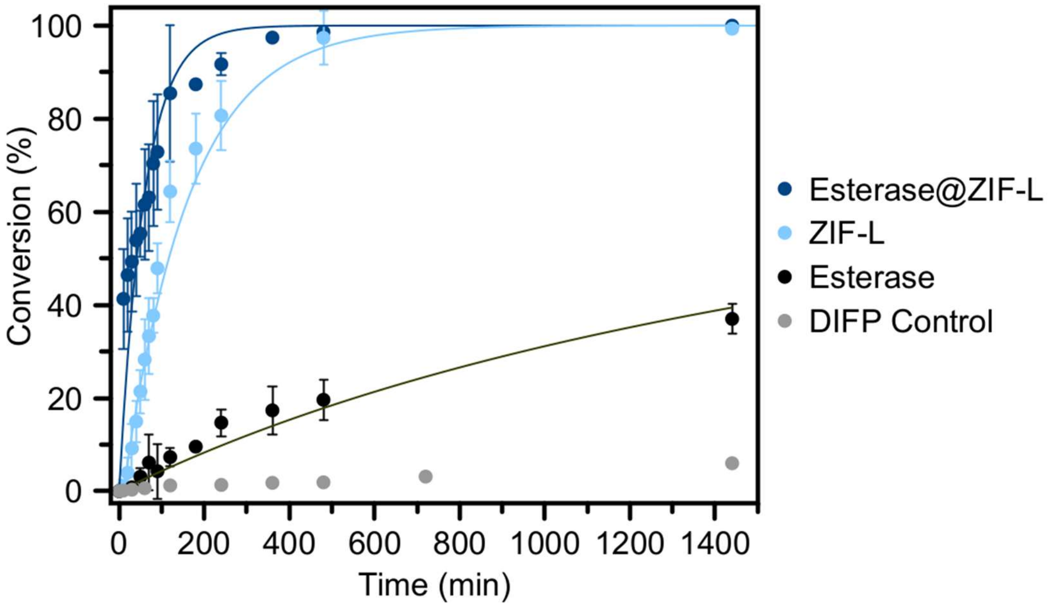

Figure S41. Profiles of diisopropylfluorophosphate hydrolytic degradation by Esterase, ZIF-L and Esterase@ZIF-L materials in simulated biological conditions. Experimental conditions: DIFP (0.029 M), 0.084 mmol of ZIF (in the case of Esterase, we considered the same amount of Esterase employed in the Esterase@ZIF material), DMF (0.029 M, internal reference) and Tris-HCl (0.1 M, pH = 7.4, 0.5 mL) at room temperature. Profile of DIFP degradation in the absence of these materials is represented in grey dots.

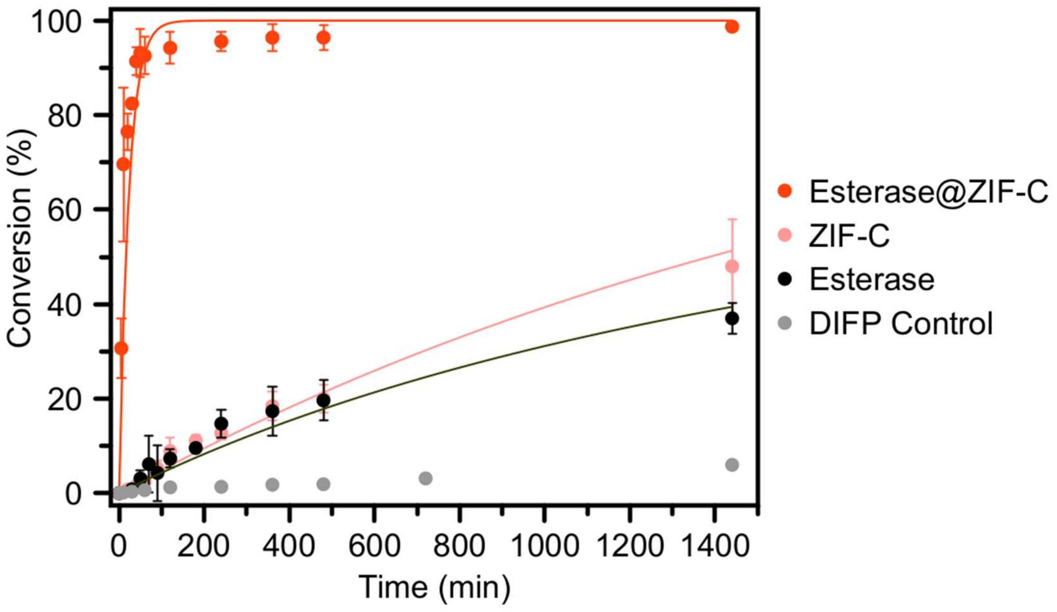

Figure S42. Profiles of diisopropylfluorophosphate hydrolytic degradation by Esterase, ZIF-C and Esterase@ZIF-C materials in simulated biological conditions. Experimental conditions: DIFP (0.029 M), 0.084 mmol of ZIF (in the case of Esterase, we considered the same amount of Esterase employed in the Esterase@ZIF material), DMF (0.029 M, internal reference) and Tris-HCl (0.1 M, pH = 7.4, 0.5 mL) at room temperature. Profile of DIFP degradation in the absence of these materials is represented in grey dots.

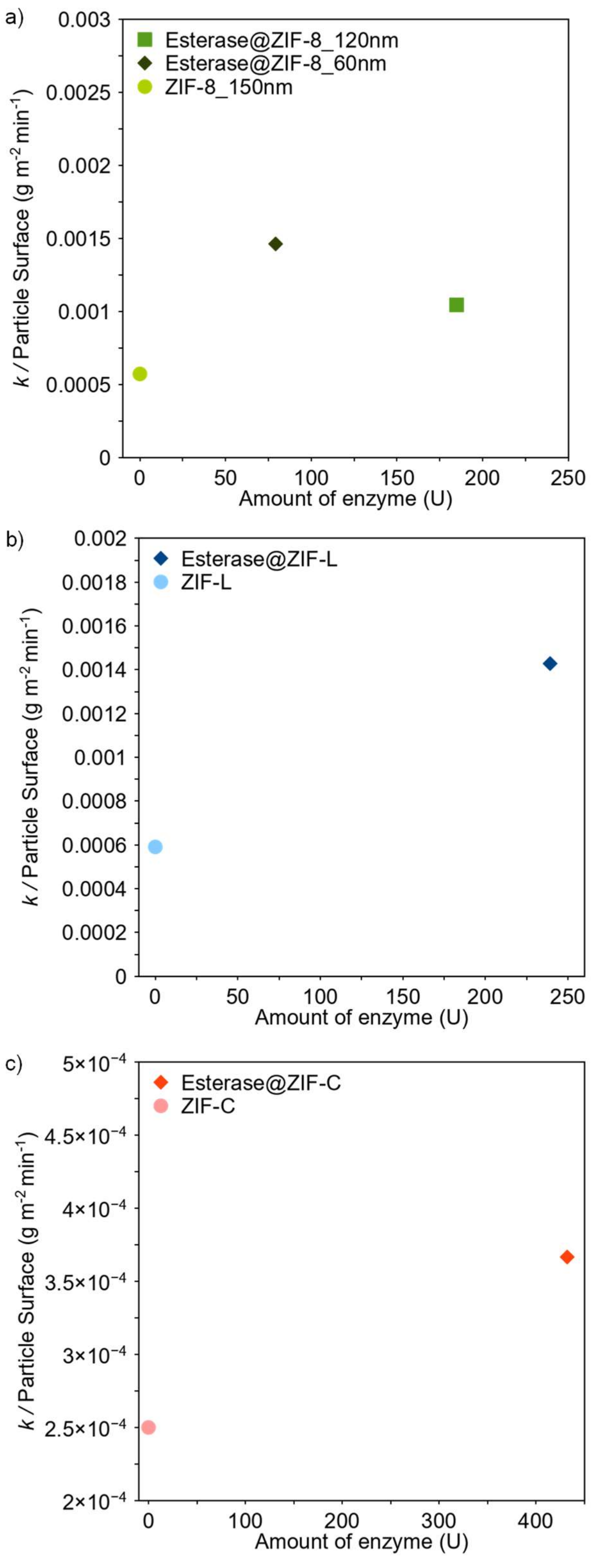

Figure S43. Relationship between kinetics constant normalized to the external particle surface area vs the amount of enzyme activity units for (a) ZIF-8, (b) ZIF-L and (c) ZIF-C biocomposites.

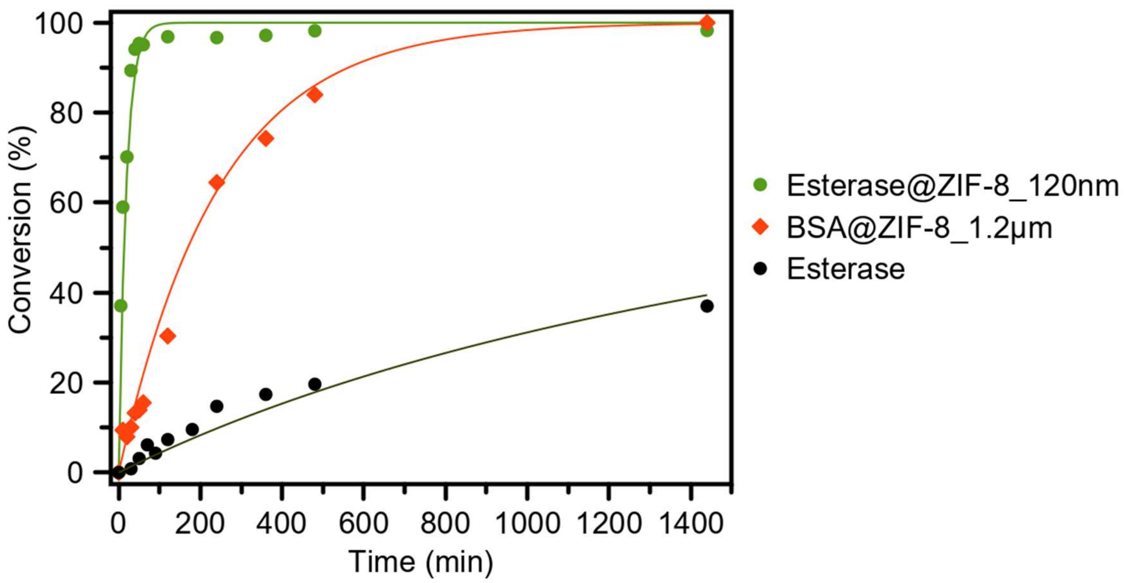

Figure S44. Profiles of diisopropylfluorophosphate hydrolytic degradation by Esterase@ZIF-8_120nm, BSA@ZIF-8_1.2µm and free Esterase in simulated biological conditions as control experiments. Experimental conditions: DIFP (0.029 M), 0.084 mmol of ZIF, DMF (0.029 M, internal reference) and Tris-HCl (0.1 M, pH = 7.4, 0.5 mL) at room temperature.

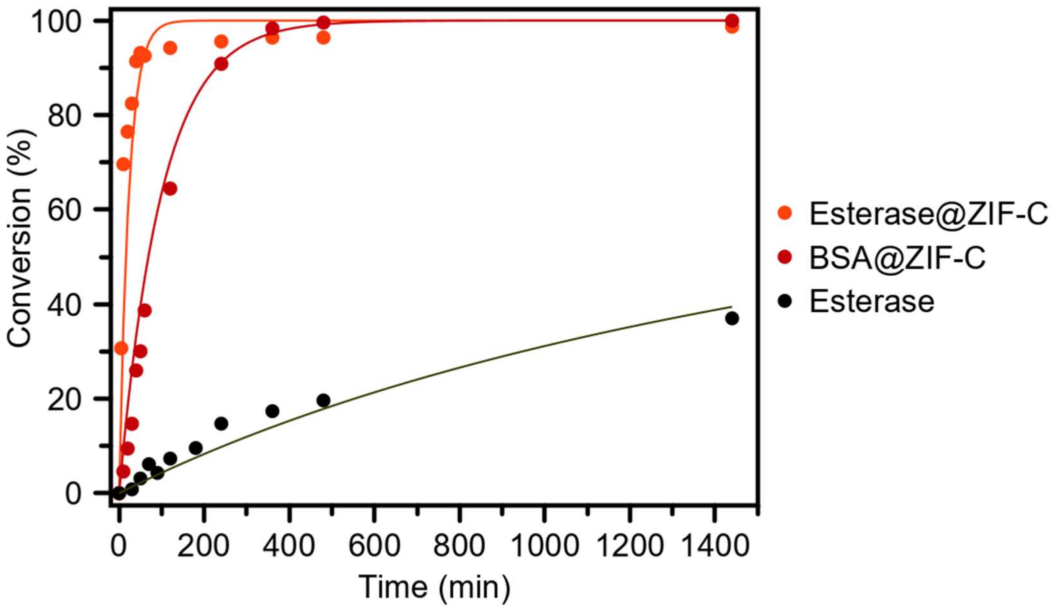

Figure S45. Profiles of diisopropylfluorophosphate hydrolytic degradation by Esterase@ZIF-C, BSA@ZIF-C and free Esterase in simulated biological conditions as control experiments. Experimental conditions: DIFP (0.029 M), 0.084 mmol of ZIF, DMF (0.029 M, internal reference) and Tris-HCl (0.1 M, pH = 7.4, 0.5 mL) at room temperature.

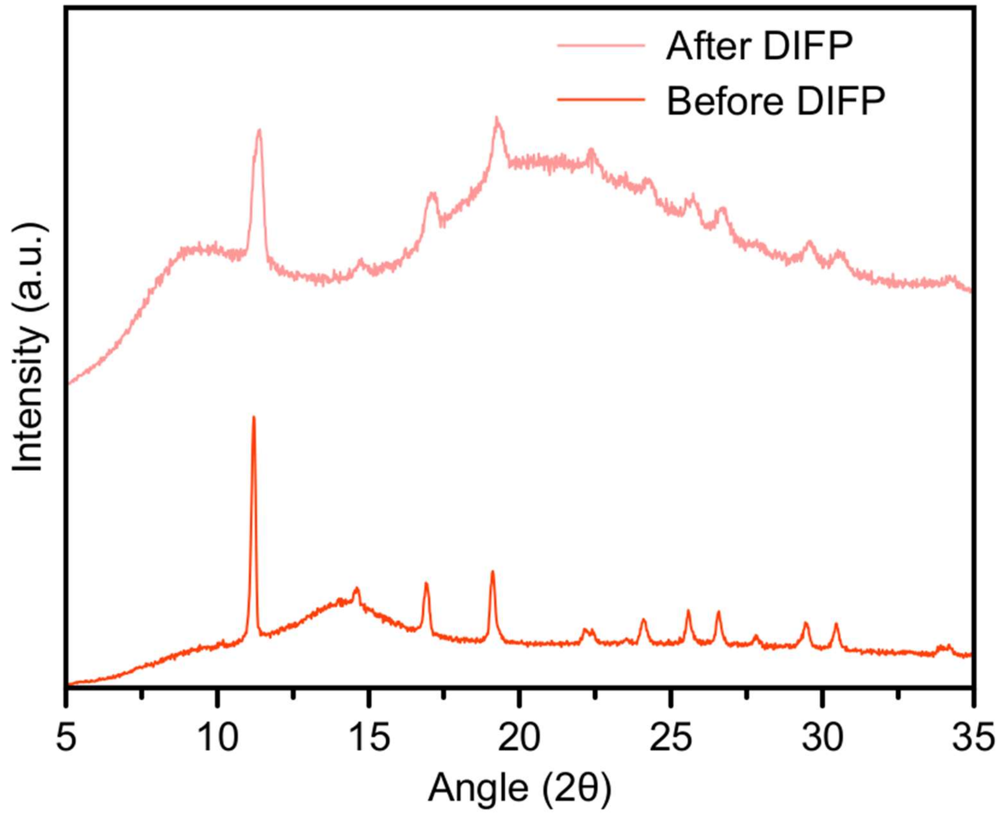

Figure S46. Powder X-Ray Diffraction patterns of BSA@ZIF-C biocomposite before and after DIFP reaction. Experimental conditions: DIFP (0.029 M), BSA@ZIF-C (0.084 mmol of ZIF) and Tris-HCl (0.1 M, pH = 7.4, 0.5 mL) at room temperature.

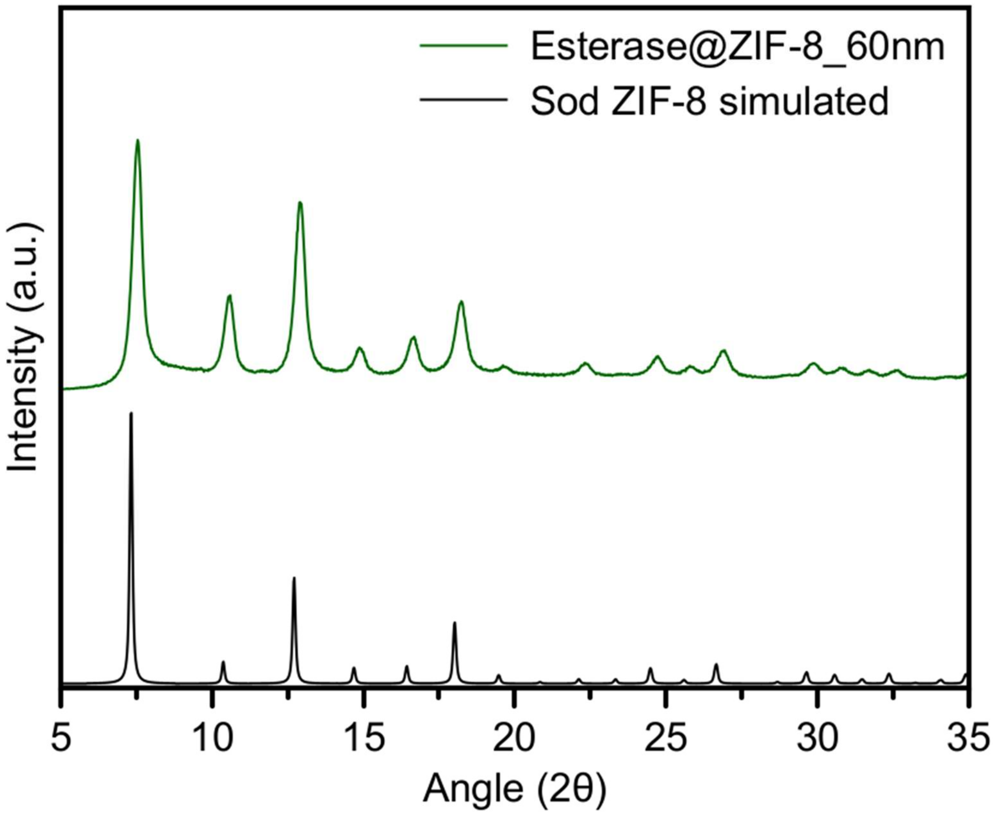

Figure S47. Powder X-Ray Diffraction pattern of Esterase@ZIF-8_60nm biocomposite (experimental, dark green; sod ZIF-8 calculated, black).

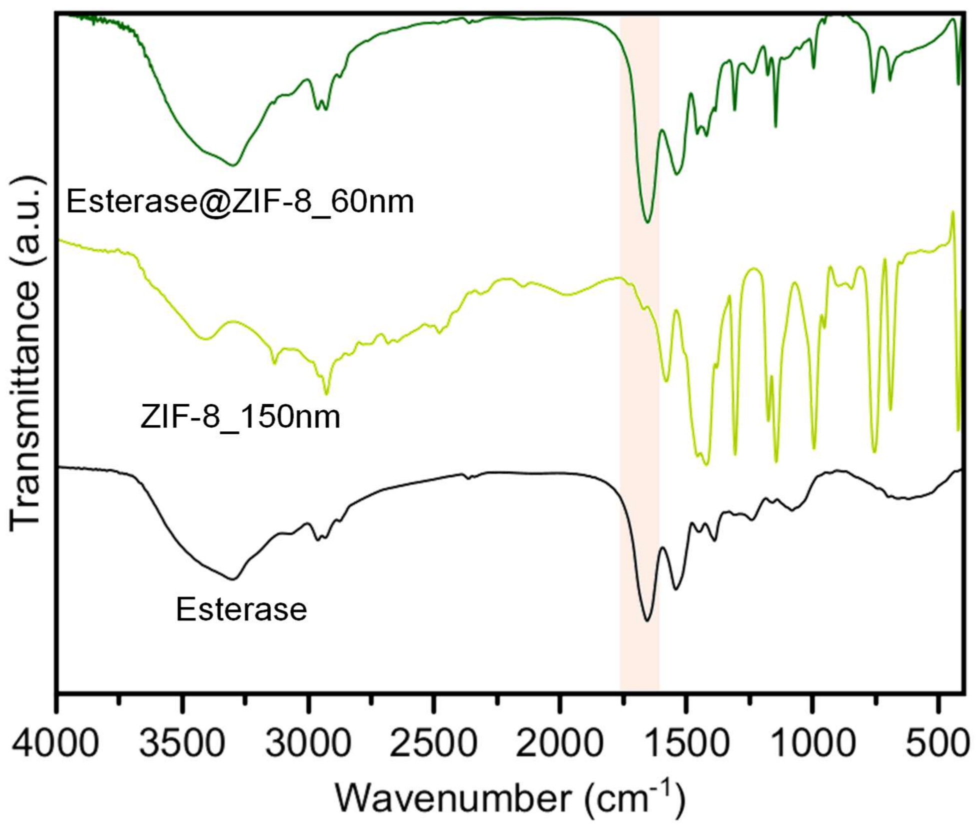

Figure S48. Fourier Transformed Infrared spectra (FTIR) of Esterase (black), ZIF-8_150nm (light green) and Esterase@ZIF-8_60nm (dark green) materials. The analysis of the FTIR data confirms the presence of the characteristic Amide I band (1700-1610 cm^{-1}) of the peptide backbone of Esterase highlighted in light orange.

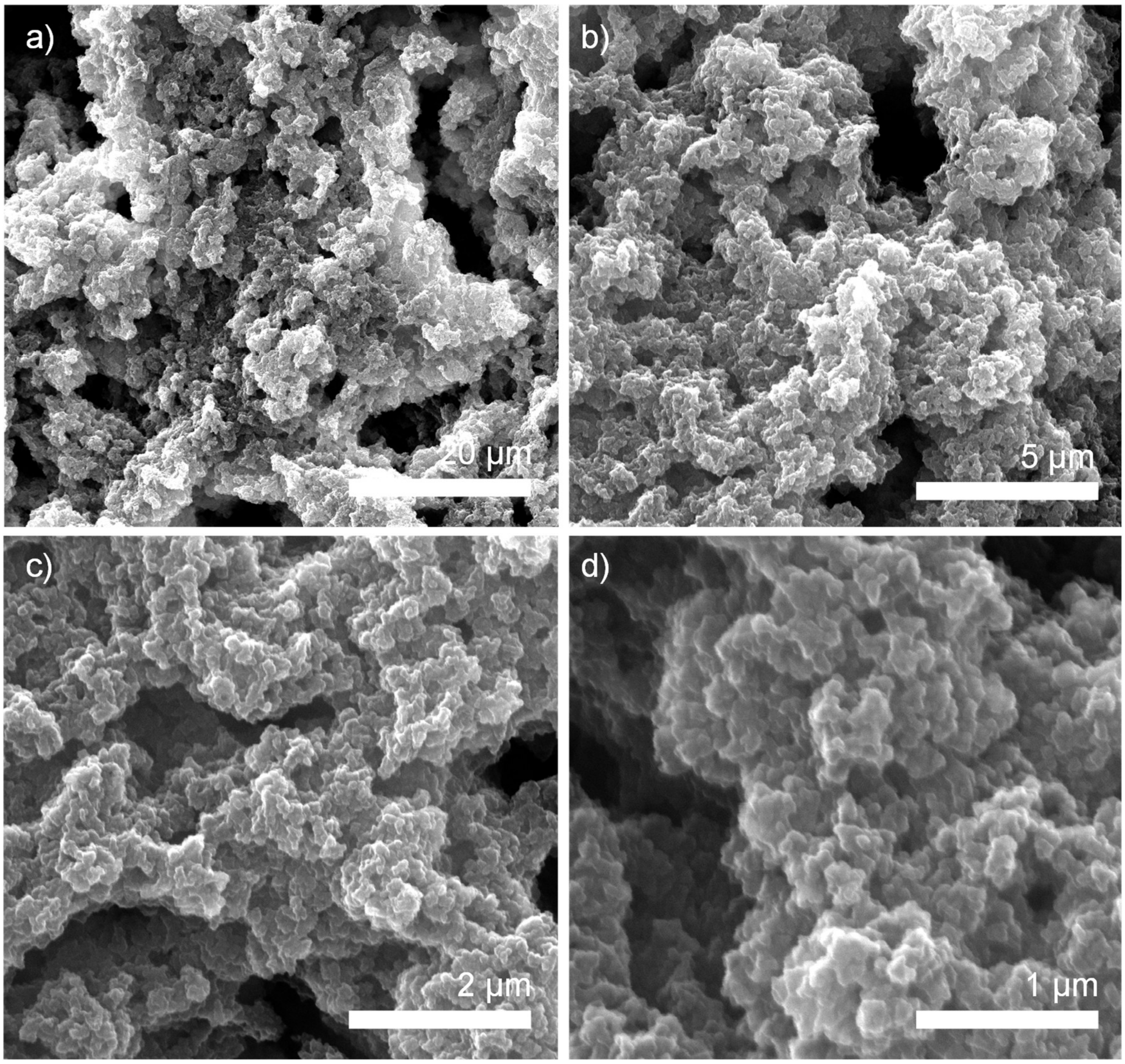

Figure S49. Scanning Electron Microscopy (SEM) images of Esterase@ZIF-8_60nm at different magnifications: a) 20 µm, b) 5 µm, c) 2 µm and d) 1 µm.

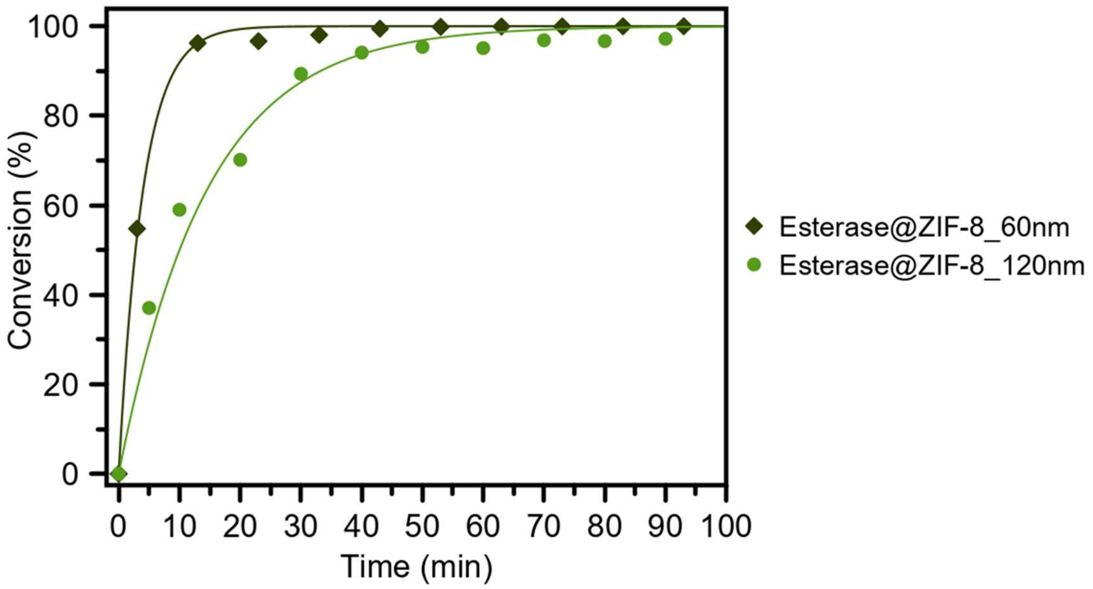

Figure S50. Profiles of diisopropylfluorophosphate hydrolytic degradation by Esterase@ZIF-8 biocomposites of two different particle size: Esterase@ZIF-8_60nm and Esterase@ZIF-8_120nm in simulated biological conditions. Experimental conditions: DIFP (0.029 M), 0.084 mmol of ZIF (we considered the amount of ZIF employed in each Esterase@ZIF material), DMF (0.029 M, internal reference) and Tris-HCl (0.1 M, pH = 7.4, 0.5 mL) at room temperature.

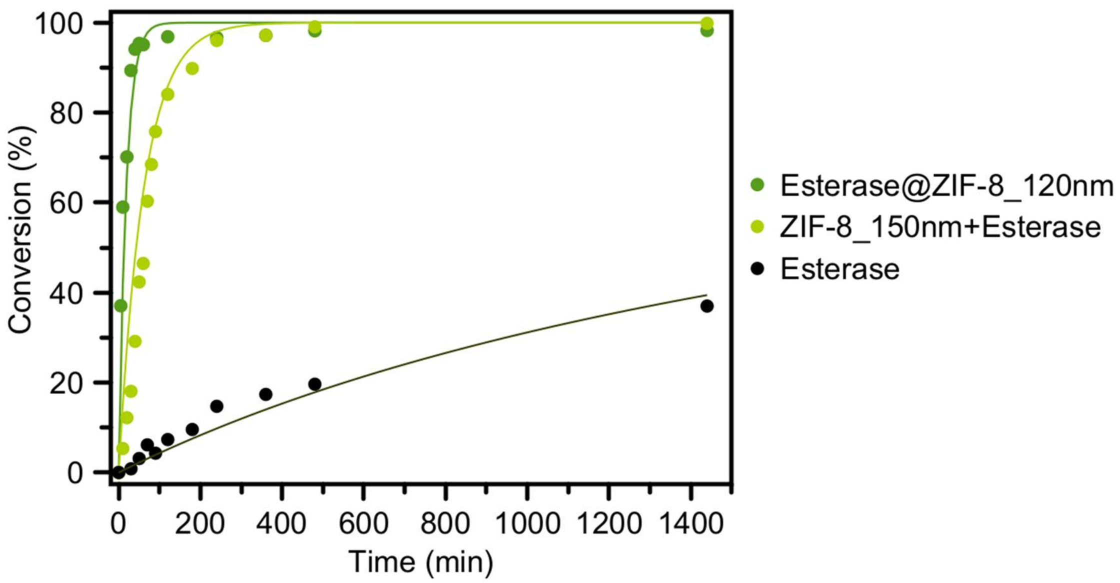

Figure S51. Profiles of diisopropylfluorophosphate hydrolytic degradation by Esterase@ZIF-8_120nm, ZIF-8_150nm + Esterase physical mixture and free Esterase in simulated biological conditions. Experimental conditions: DIFP (0.029 M), 0.084 mmol of ZIF (in the case of ZIF-8_150nm+Esterase physical mixture, we considered the amount of sod ZIF-8 and Esterase employed in the Esterase@ZIF-8_120nm material), DMF (0.029 M, internal reference) and Tris-HCl (0.1 M, pH = 7.4, 0.5 mL) at room temperature.

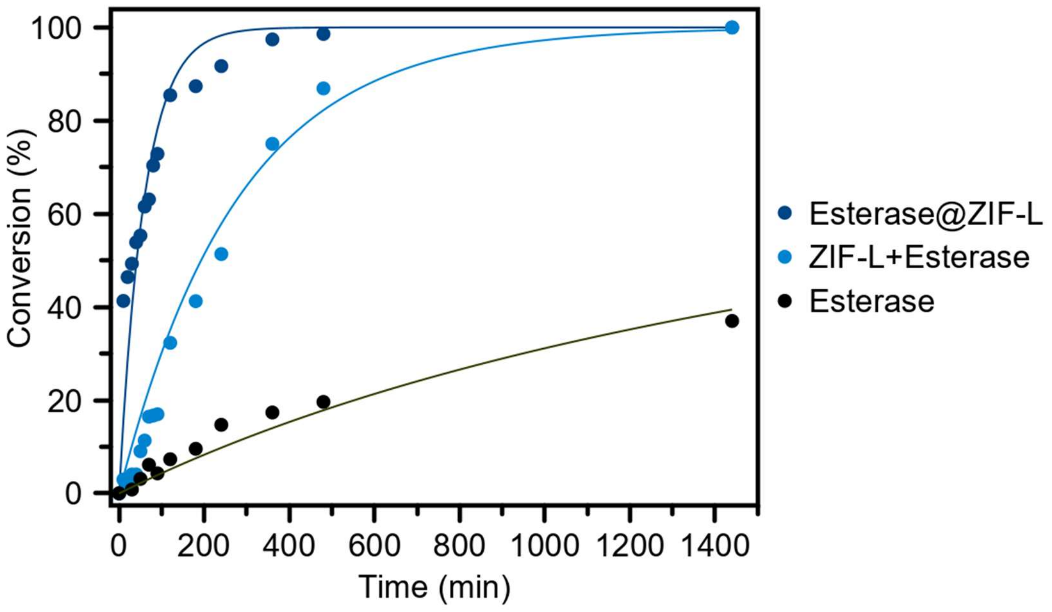

Figure S52. Profiles of diisopropylfluorophosphate hydrolytic degradation by Esterase@ZIF-L, ZIF-L + Esterase physical mixture and free Esterase in simulated biological conditions. Experimental conditions: DIFP (0.029 M), 0.084 mmol of ZIF (in the case of ZIF-L+Esterase physical mixture, we considered the amount of ZIF-L and Esterase employed in the Esterase@ZIF-L material), DMF (0.029 M, internal reference) and Tris-HCl (0.1 M, pH = 7.4, 0.5 mL) at room temperature.

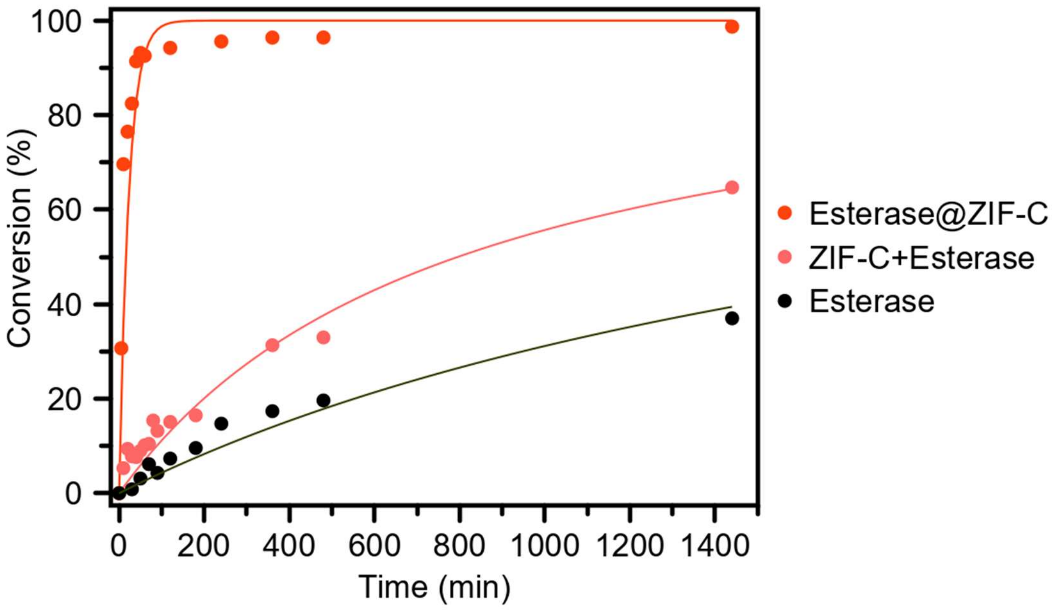

Figure S53. Profiles of diisopropylfluorophosphate hydrolytic degradation by Esterase@ZIF-C, ZIF-C + Esterase physical mixture and free Esterase in simulated biological conditions. Experimental conditions: DIFP (0.029 M), 0.084 mmol of ZIF (in the case of ZIF-C+Esterase physical mixture, we considered the amount of ZIF-C and Esterase employed in the Esterase@ZIF-C material), DMF (0.029 M, internal reference) and Tris-HCl (0.1 M, pH = 7.4, 0.5 mL) at room temperature.

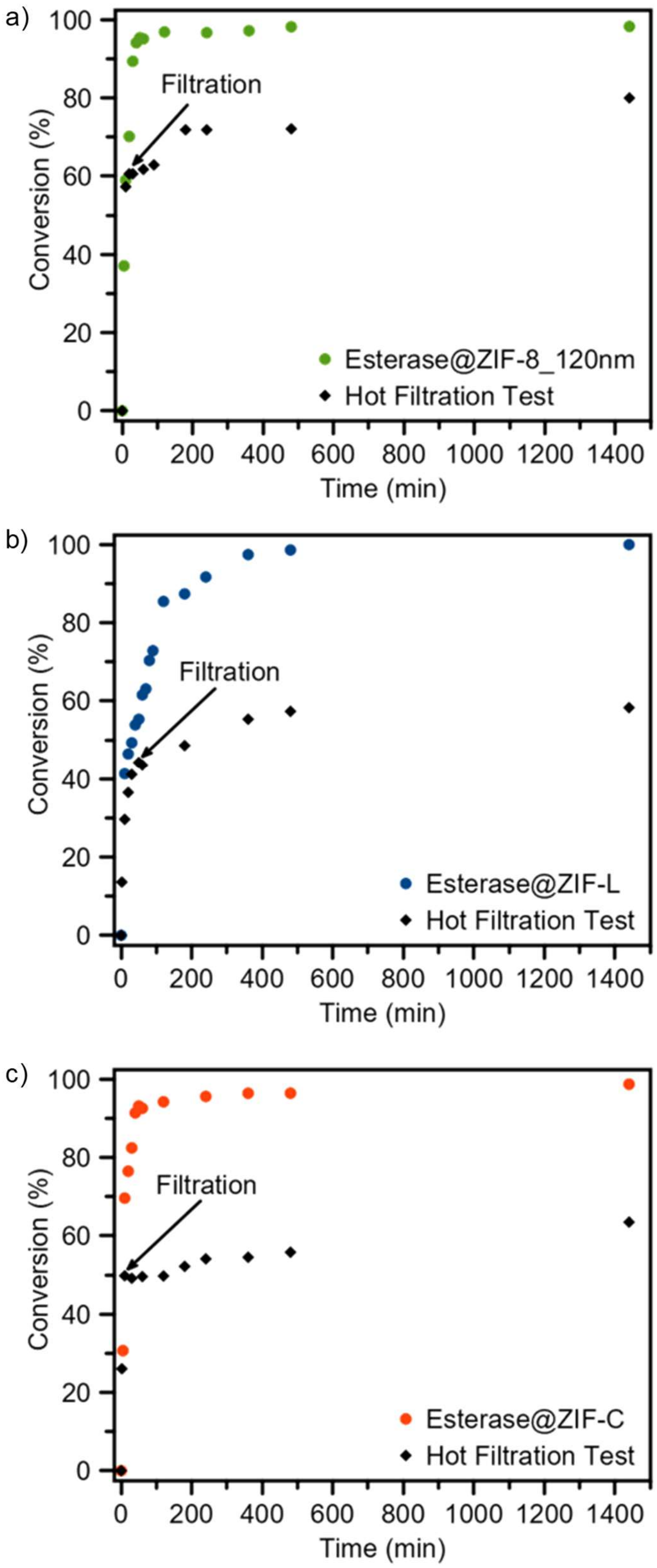

Figure S54. Hot filtration tests profiles of diisopropylfluorophosphate (DIFP) hydrolytic degradation by (a) Esterase@ZIF-8_120nm, (b) Esterase@ZIF-L and (c) Esterase@ZIF-C materials in simulated biological conditions. Experimental conditions: DIFP (0.029 M), 0.084 mmol of ZIF, DMF (0.029 M, internal reference) and Tris-HCl (0.1 M, pH = 7.4, 0.5 mL) at room temperature.

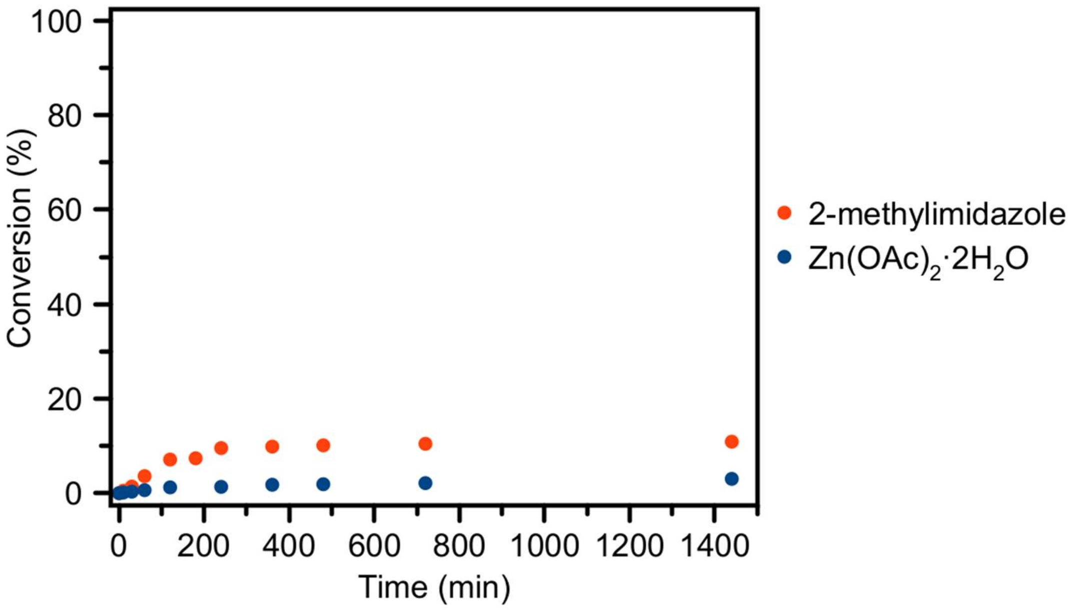

Figure S55. Profiles of diisopropylfluorophosphate hydrolytic degradation by 2-methylimidazole and $Zn(OAc)_2 \cdot 2H_2O$ control experiments in simulated biological conditions. Experimental conditions: DIFP (0.029 M), 0.029 M of 2-methylimidazole/$Zn(OAc)_2 \cdot 2H_2O$, DMF (0.029 M, internal reference) and Tris-HCl (0.1 M, pH = 7.4, 0.5 mL) at room temperature.

S3.3. Esterase enzymatic activity of biocomposites

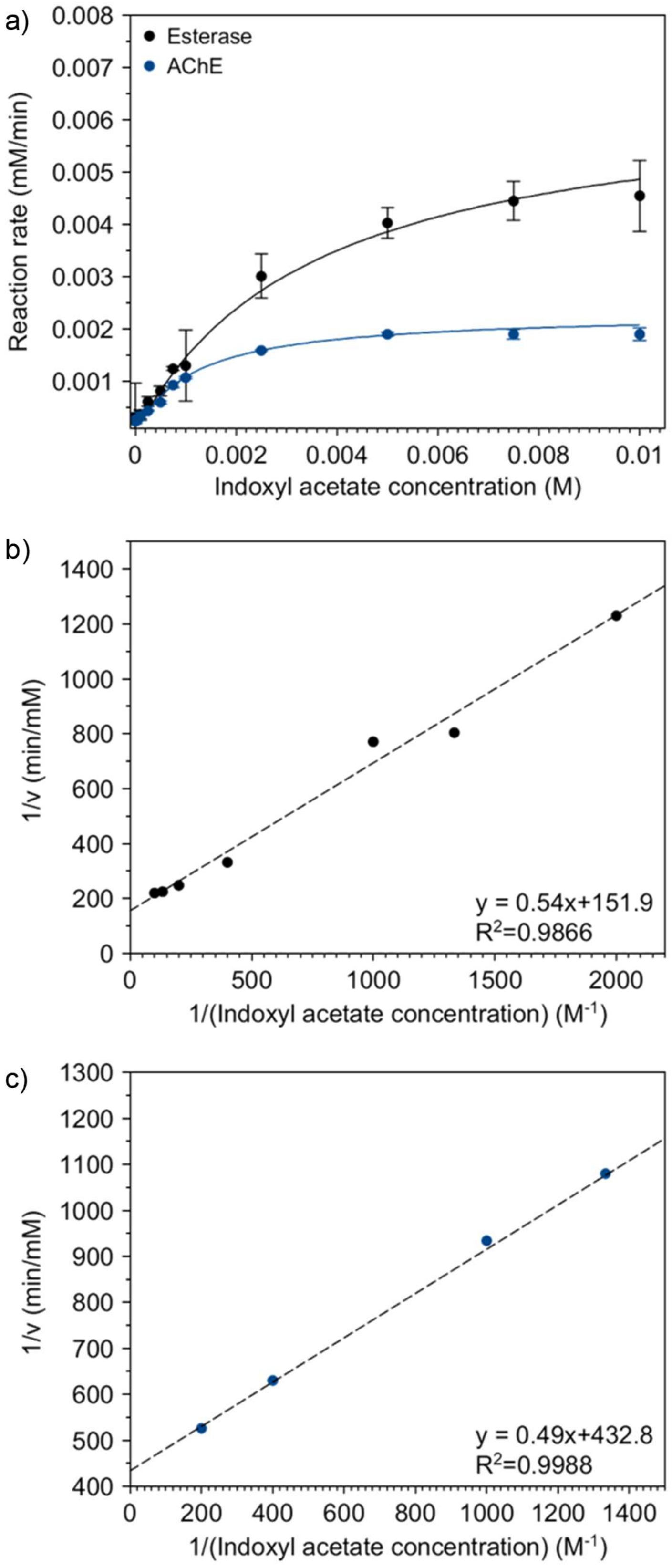

Figure S56. (a) Michaelis-Menten kinetic for Esterase (black) and AChE (blue) for indoxyl acetate test. Lineweaver-Burk double reciprocal plot adjustment for (b) Esterase and (c) AChE enzymes. See S2.4.1. for further experimental details.

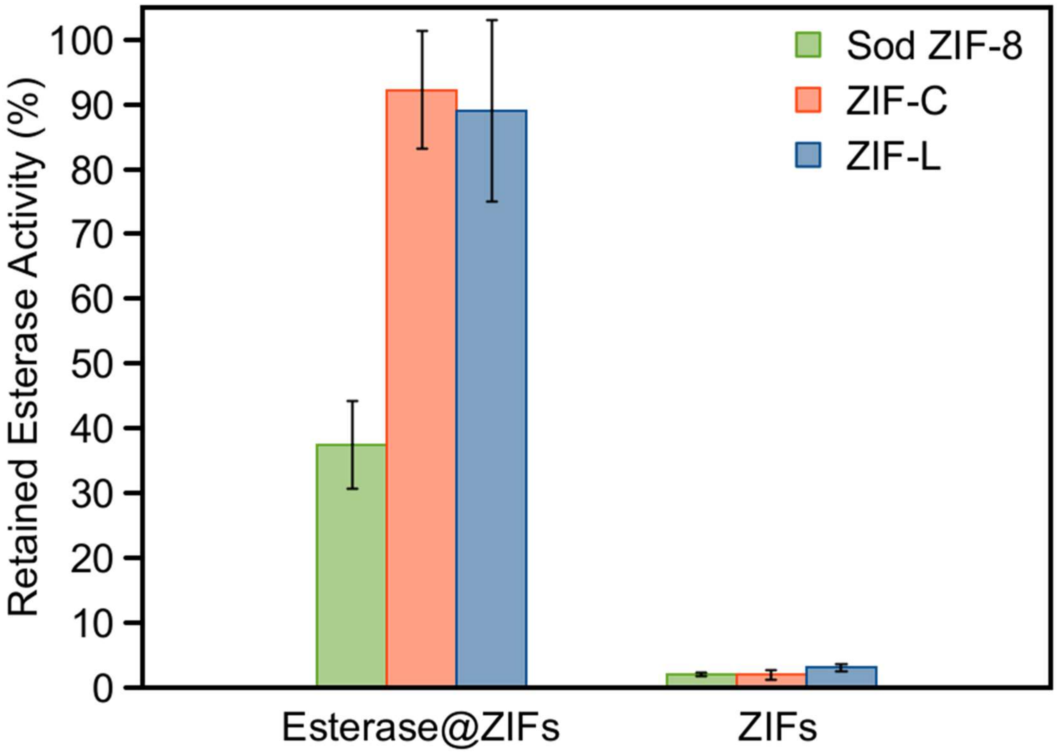

Figure S57. Retained enzymatic Esterase activity of Esterase@ZIFs after the biomineralization process. The enzymatic activity of the three new biocomposites (Esterase@ZIF-8_120nm, Esterase@ZIF-C and Esterase@ZIF-L) was compared to the enzymatic activity of free Esterase at 37 ºC. In addition, we also compared the enzymatic activity of ZIFs (without Esterase) as control. See S.2.4.3. for further experimental details.

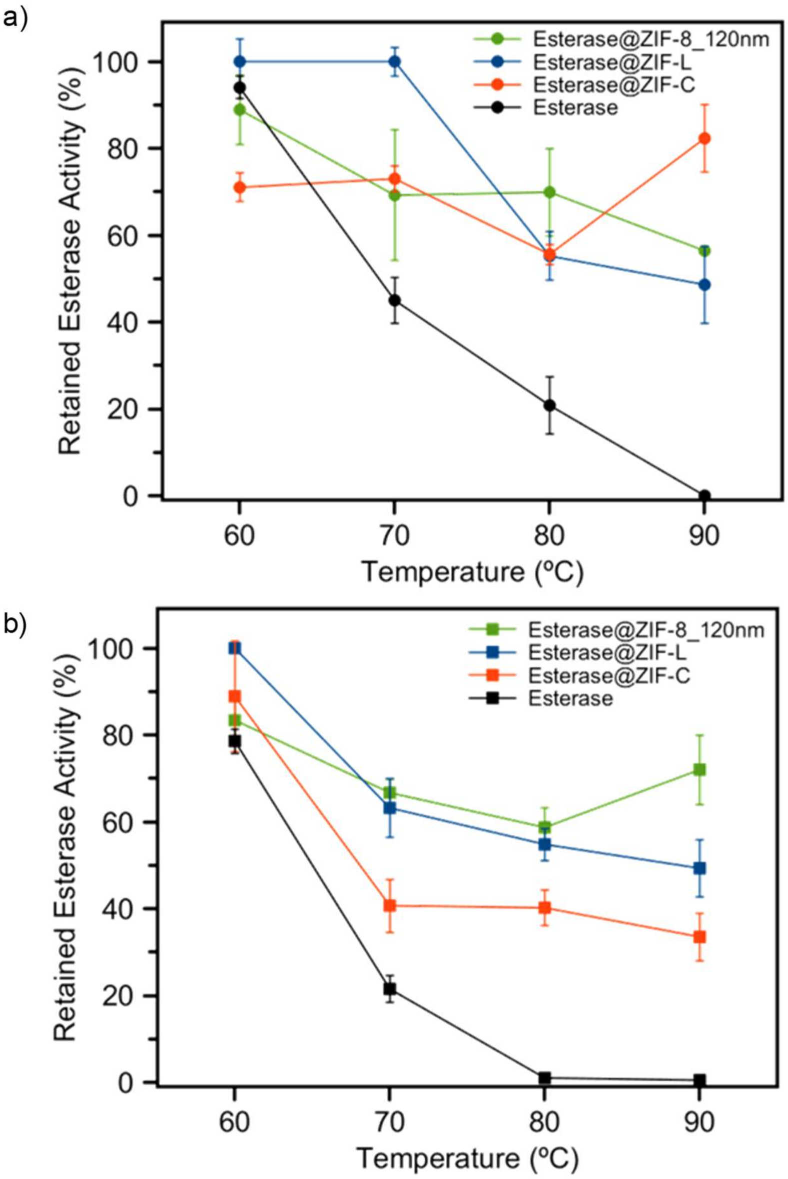

Figure S58. Retained enzymatic activity of free Esterase, Esterase@ZIF-8_120nm, Esterase@ZIF-L and Esterase@ZIF-C materials after incubation at different temperatures for (a) 30 min and (b) 1 h. See S.2.4.5. for further experimental details.

Table S18. Protein secondary structure assignment according to deconvoluted peaks

Average Position (cm^{-1})	Assignment	Reference
1600-1620	Intermolecular β-sheet	12
1620-1640, 1680-1700	β-sheet	12–17
1640-1650	Random coil	13,15
1648-1660	α-helix	13,15
1660-1680	β-turn	12–15

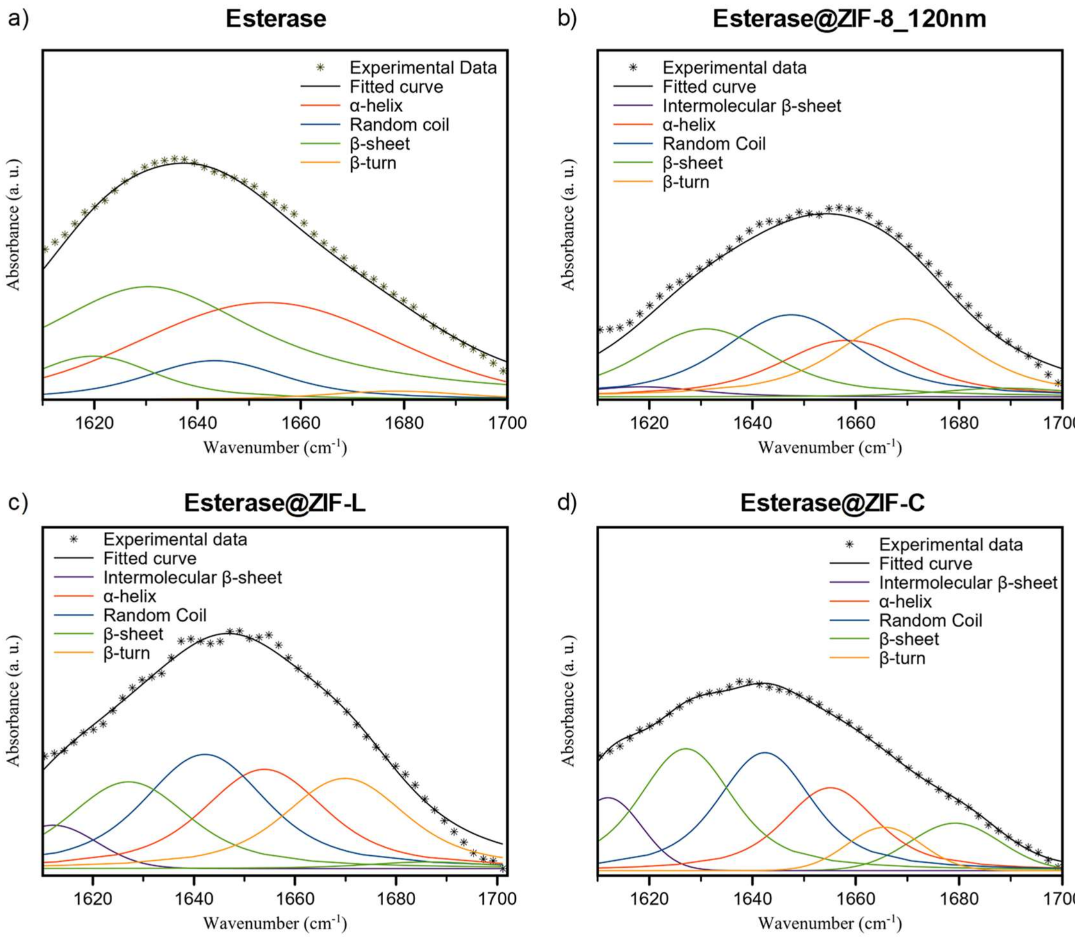

Figure S59. ATR-FTIR spectra and the corresponding second derivative ATR-FTIR spectra of (a) Esterase (b) Esterase@ZIF-8_120nm, (c) Esterase@ZIF-L and (d) Esterase@ZIF-C showing the structural content of intermolecular β-sheet, α-helix, random coil, β-sheet and β-turn. The structural contents were determined by the Voigt function fitted in Origin software.

Table S19. Protein secondary structure assignment according to deconvoluted peaks for studied materials

	β-sheet (%)	β-turn (%)	α-helix (%)	Random coil (%)	Intramolecular β-sheet (%)
Free Esterase	56.9	2.0	32.1	9.0	0
Esterase@ZIF-8_120nm	25.2	25.9	18.7	27.2	3.0
Esterase@ZIF-C	36.9	6.3	19.4	27.8	9.6
Esterase@ZIF-L	22.1	21.4	23.6	27.1	5.8

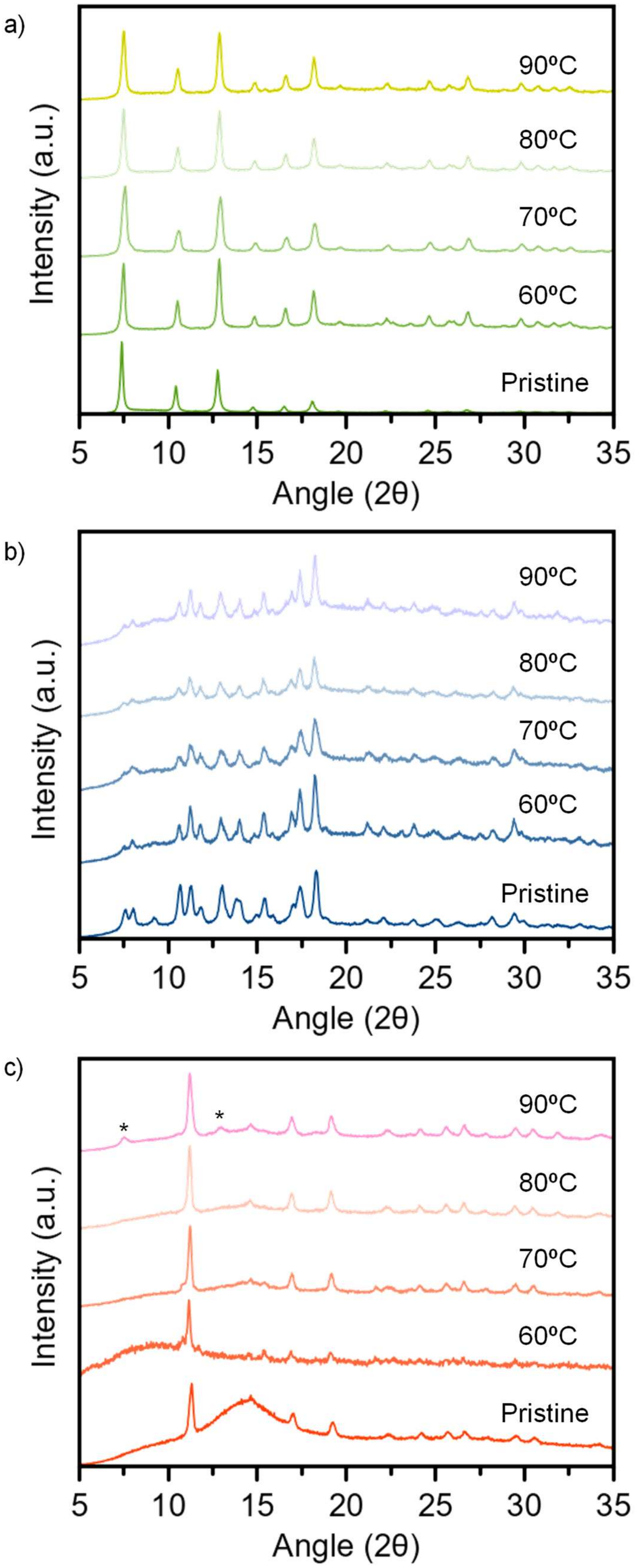

Figure S60. Powder X-Ray Diffraction patterns of (a) Esterase@ZIF-8_120nm, (b) Esterase@ZIF-L and (c) Esterase@ZIF-C biocomposites after different incubation temperatures. Experimental conditions: Esterase@ZIF (1.87 U/mL of Esterase) and Tris-HCl (0.1 M, pH = 7.4, 1 mL) incubated at different temperatures for 1 h. Asterisk: appearance of sodalite phase.

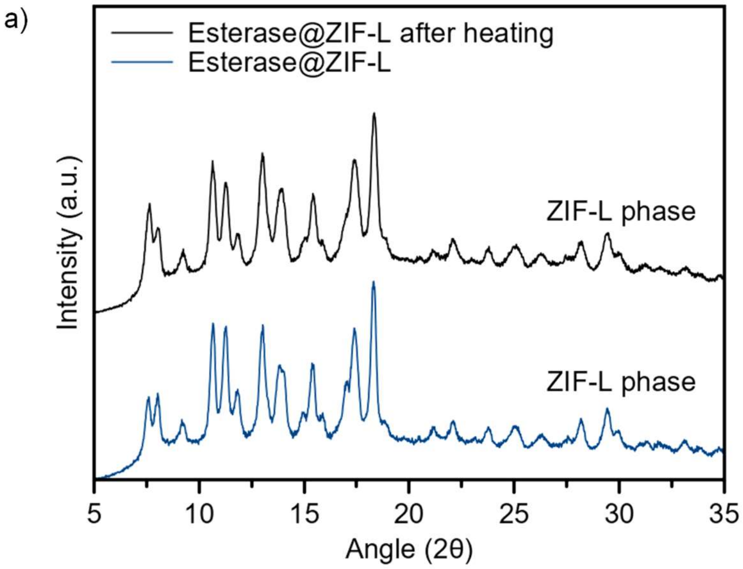

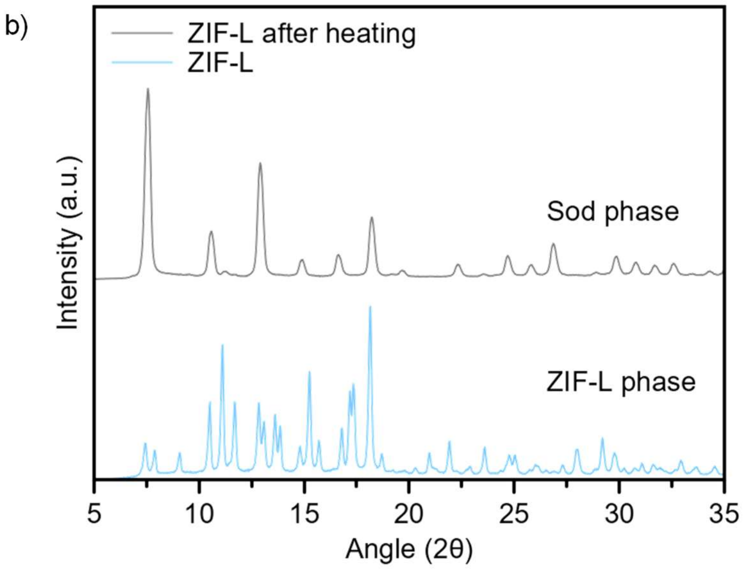

Figure S61. Powder X-Ray Diffraction patterns of (a) Esterase@ZIF-L and (b) ZIF-L materials after incubation in EtOH at 60 ºC. Experimental conditions: ZIF-L or Esterase@ZIF-L (50 mg) were dispersed in ethanol (5 mL) and heated to 60 °C for 72 h. This was left to cool before separation by centrifuge at 6000 rpm for 10 min. The samples were dried at 60 °C overnight. The results indicate that ZIF-L crystalline phase is well maintained in the Esterase@ZIF biocomposite, probably due to a stabilization with the esterase encapsulation. In the ZIF-L material alone, there is a crystal phase change to the more stable sodalite phase as reported in literature[18].

S3.4. DIFP induced ZIF crystal surface degradation.

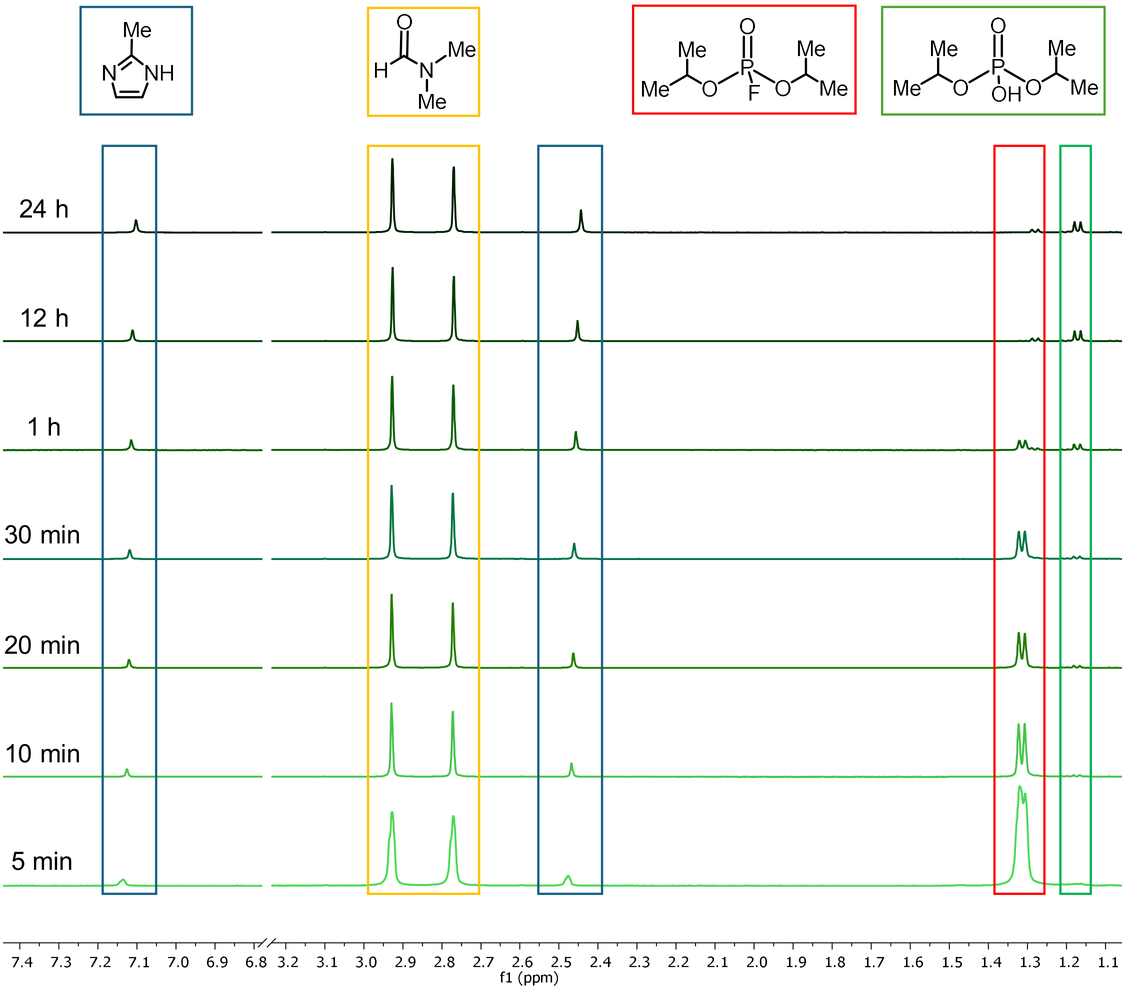

Figure S62. ^{1}H-NMR follow-up of diisopropylfluorophosphate (DIFP) hydrolysis and ZIF structural degradation with concomitant mImH release by Esterase@ZIF-8_120nm biocomposite. Experimental conditions: DIFP (0.029 M), Esterase@ZIF-8_120nm (0.084 mmol of sod ZIF-8), dimethylacetamide (0.029 M, internal reference) and Tris-DCl (0.1 M, pD = 7.8, 0.5 mL) at room temperature.

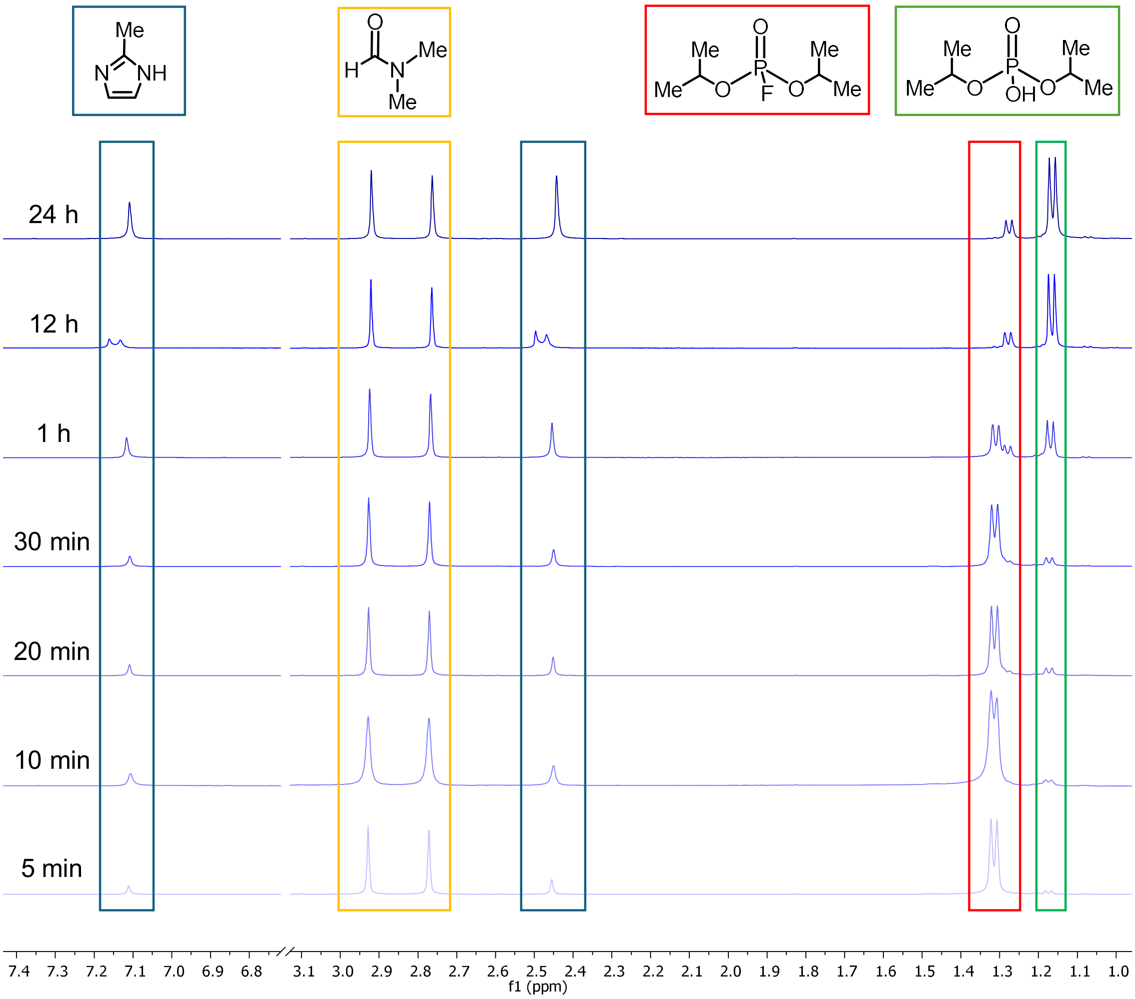

Figure S63. ^{1}H-NMR follow-up of diisopropylfluorophosphate (DIFP) hydrolysis and ZIF structural degradation with concomitant mImH release by Esterase@ZIF-L biocomposite. Experimental conditions: DIFP (0.029 M), Esterase@ZIF-L (0.084 mmol of ZIF-L), dimethylacetamide (0.029 M, internal reference) and Tris-DCl (0.1 M, pD = 7.8, 0.5 mL) at room temperature.

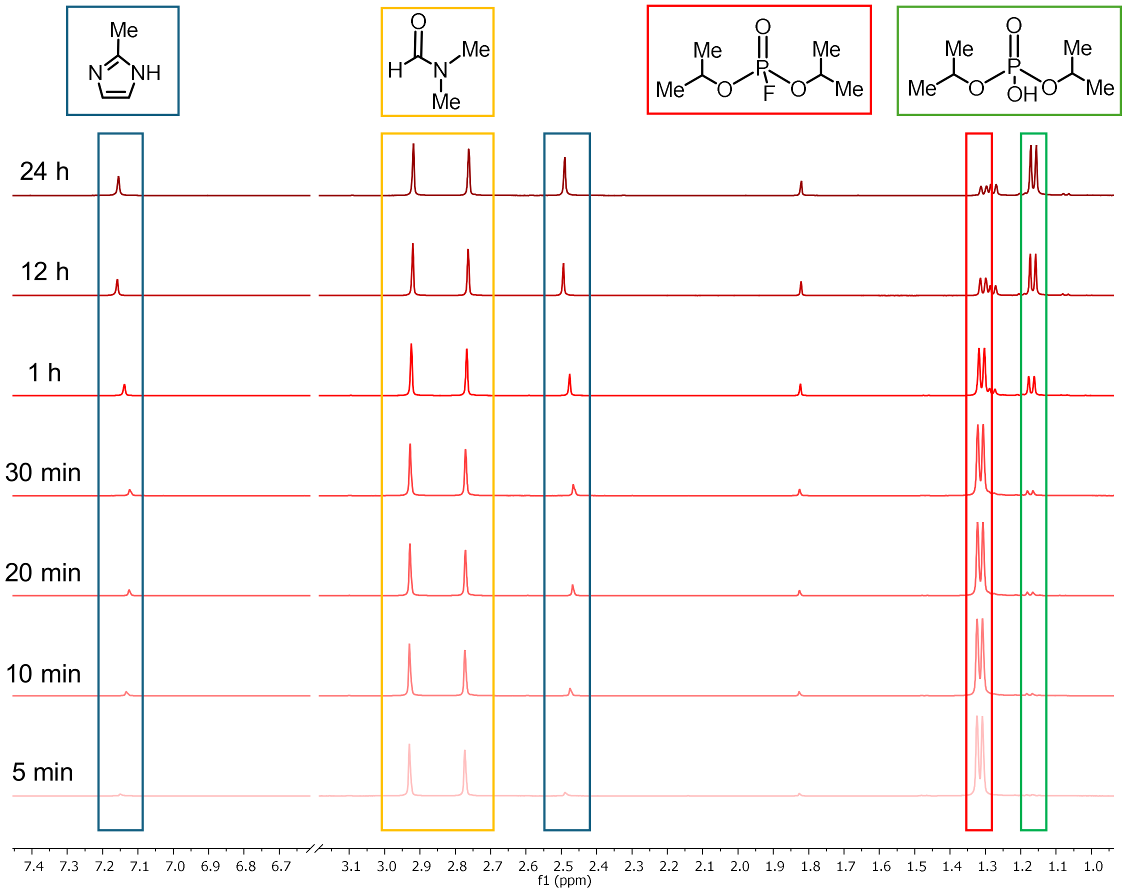

Figure S64. ^{1}H-NMR follow-up of diisopropylfluorophosphate (DIFP) hydrolysis and ZIF structural degradation with concomitant mImH release by Esterase@ZIF-C biocomposite. Experimental conditions: DIFP (0.029 M), Esterase@ZIF-C (0.084 mmol of ZIF-C), dimethylacetamide (0.029 M, internal reference) and Tris-DCl (0.1 M, pD = 7.8, 0.5 mL) at room temperature.

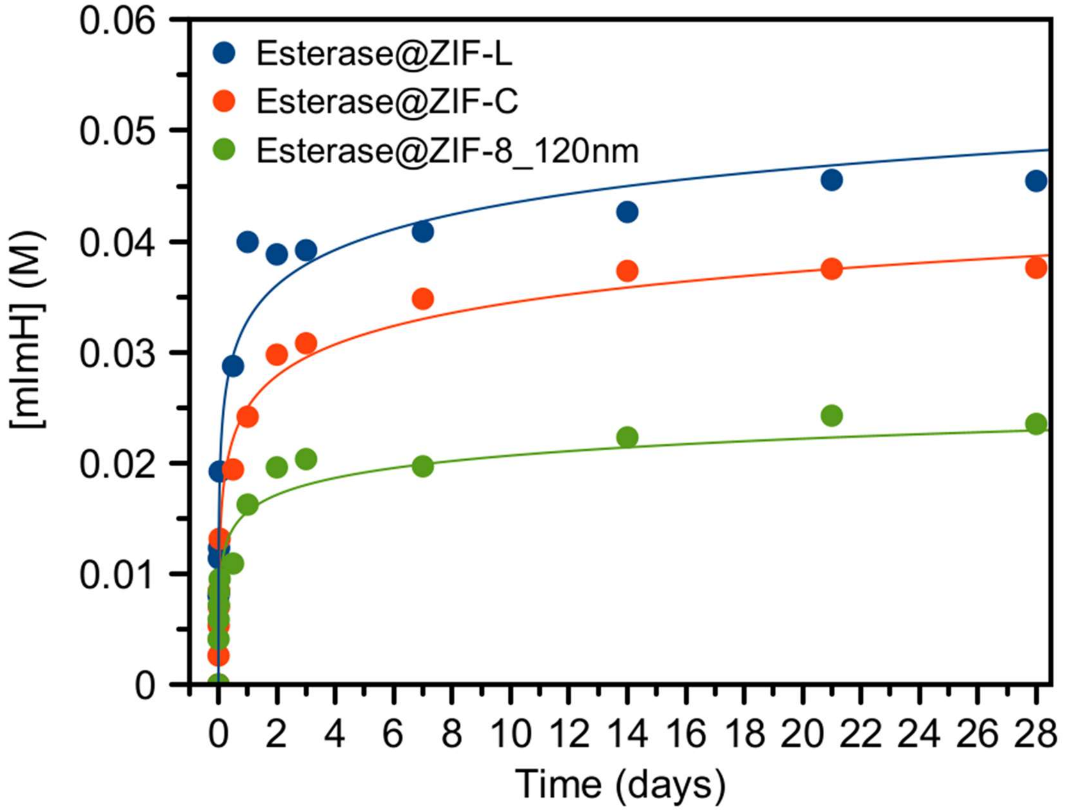

Figure S65. 2-methylimidazole release profile for Esterase@ZIF-8_120nm (green), Esterase@ZIF-L (blue) and Esterase@ZIF-C (red) along the reaction of biocomposites with diisopropylfluorophosphate (DIFP) over several days. Experimental points were collected by ^{1}H-NMR follow-up. Experimental conditions: DIFP (0.029 M), Esterase@ZIF (0.084 mmol of ZIF), dimethylacetamide (0.029 M, internal reference) and Tris-DCl (0.1 M, pD = 7.8, 0.5 mL) at room temperature.

Table S20. Concentration of 2-methylimidazole (mImH) release by Esterase@ZIF biocomposites along different times.

Time (days)	Concentration of mImH release (mM)		
	Esterase@ZIF-8_120nm	Esterase@ZIF-L	Esterase@ZIF-C
0.0035	4.1	8.0	2.6
0.0070	5.9	8.3	5.3
0.014	7.2	11.4	7.0
0.021	8.4	12.3	8.5
0.042	9.5	19.2	13.2
0.5	10.9	28.7	19.4
1	16.2	39.9	24.2
2	19.6	38.8	29.8
3	20.3	39.2	30.8
7	19.7	40.9	34.8
14	22.3	42.7	37.3
21	24.3	45.5	37.5
28	23.5	45.5	37.6

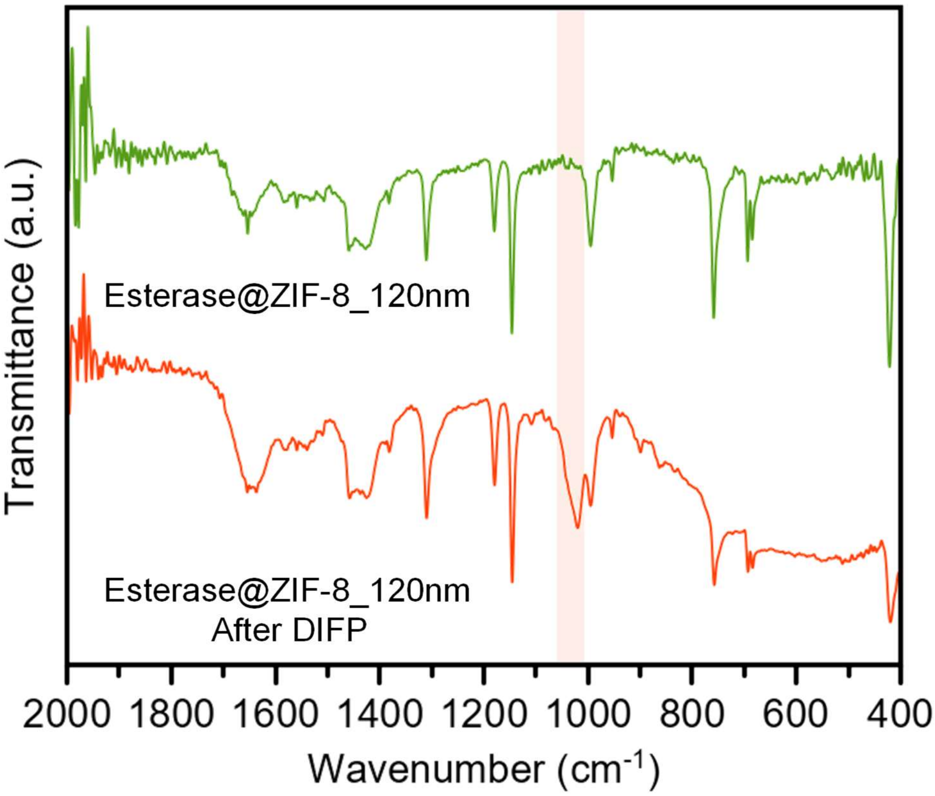

Figure 66. Attenuated total reflection-Fourier Transformed Infrared spectra (ATR-FTIR) of Esterase@ZIF-8_120nm before (green) and after (red) diisopropylfluorophosphate (DIFP) degradation. The analysis of the ATR-FTIR data after DIFP reaction confirms the presence of the characteristic P-O stretches of diisopropylphosphate in the (1000-1100 cm^{-1}) region highlighted in light orange.

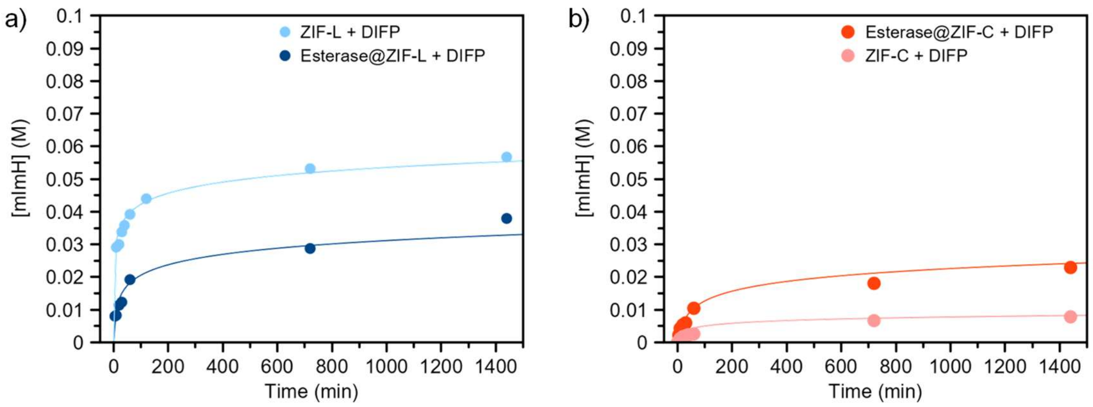

Figure S67. Comparison of 2-methylimidazole release profile for (a) Esterase@ZIF-L/ZIF-L and (b) Esterase@ZIF-C/ZIF-C materials during DIFP hydrolysis. Experimental points were collected by ^{1}H-NMR follow-up at different times. Experimental conditions: DIFP (0.029 M), 0.084 mmol of ZIF, dimethylacetamide (0.029 M, internal reference) and Tris-DCl (0.1 M, pD = 7.8, 0.5 mL) at room temperature.

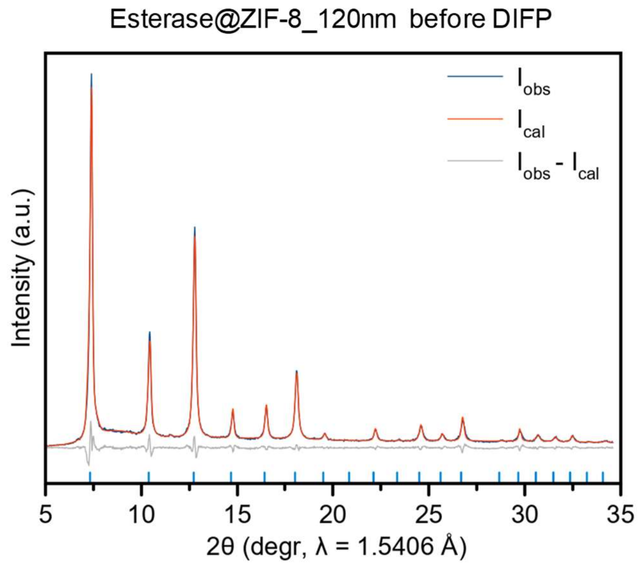

Figure S68. Graphical result of the whole powder pattern refinement carried out with the Le Bail method (R_p= 0.05738; R_{wp}= 0.07395) on the PXRD pattern of Esterase@ZIF-8_120nm before DIFP treatment in terms of observed, calculated and difference traces (blue, red and grey, respectively). The positions of the Bragg reflection are indicated by blue ticks. Cell parameters are summarized in Table S19.

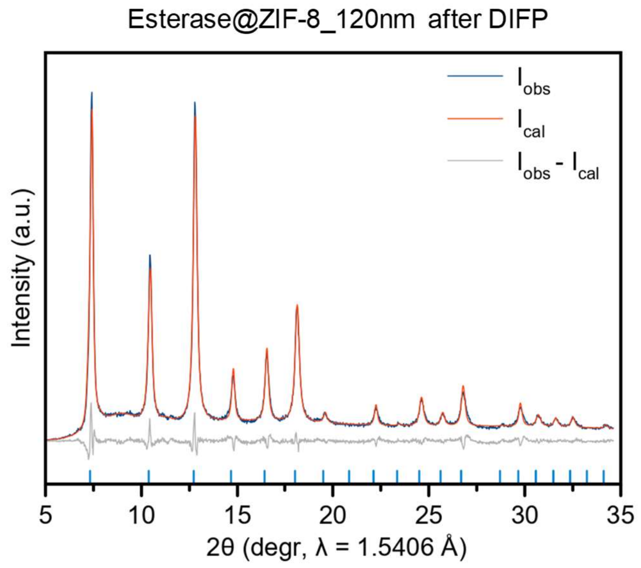

Figure S69. Graphical result of the whole powder pattern refinement carried out with the Le Bail method (R_p= 0.05633; R_{wp}= 0.07152) on the PXRD pattern of Esterase@ZIF-8_120nm after DIFP treatment in terms of observed, calculated and difference traces (blue, red and grey, respectively). The positions of the Bragg reflection are indicated by blue ticks. Cell parameters are summarized in Table S19.

Table S21. Cell parameters for Esterase@ZIF-8_120nm before and after DIFP treatment determined by PXRD Le Bail analysis. Crystalline domain size was estimated using a Lorentzian-convolution of Bragg peaks performed with the software Topas v3.

	Cell Parameter (Å)	Error (Å)	CS_L (nm)	Error_L (nm)
Esterase@ZIF-8_120nm Before DIFP	17.034	0.001	82	1
Esterase@ZIF-8_120nm After DIFP	17.031	0.002	54	1

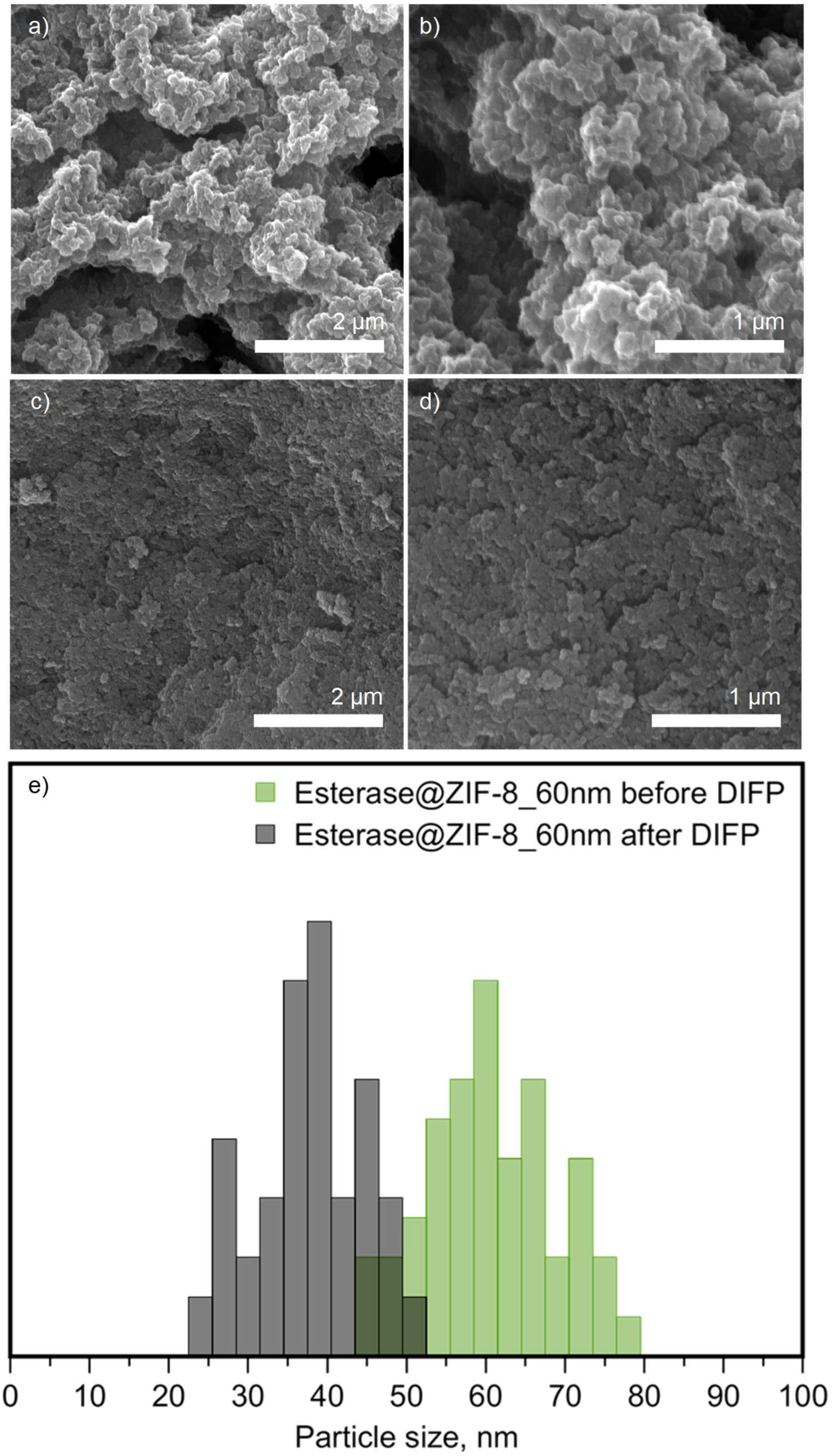

Figure S70. Scanning Electron Microscopy (SEM) images of Esterase@ZIF-8_60nm (a, b) before and (c, d) after DIFP reaction at different magnifications. (e) Particle size distribution of Esterase@ZIF-8_60nm before (green bars) and after (black bars) DIFP incubation. Experimental conditions: DIFP (0.029 M), Esterase@ZIF-8_60nm (0.084 mmol of sod ZIF-8) and Tris-HCl (0.1 M, pH = 7.4, 0.5 mL) at room temperature.

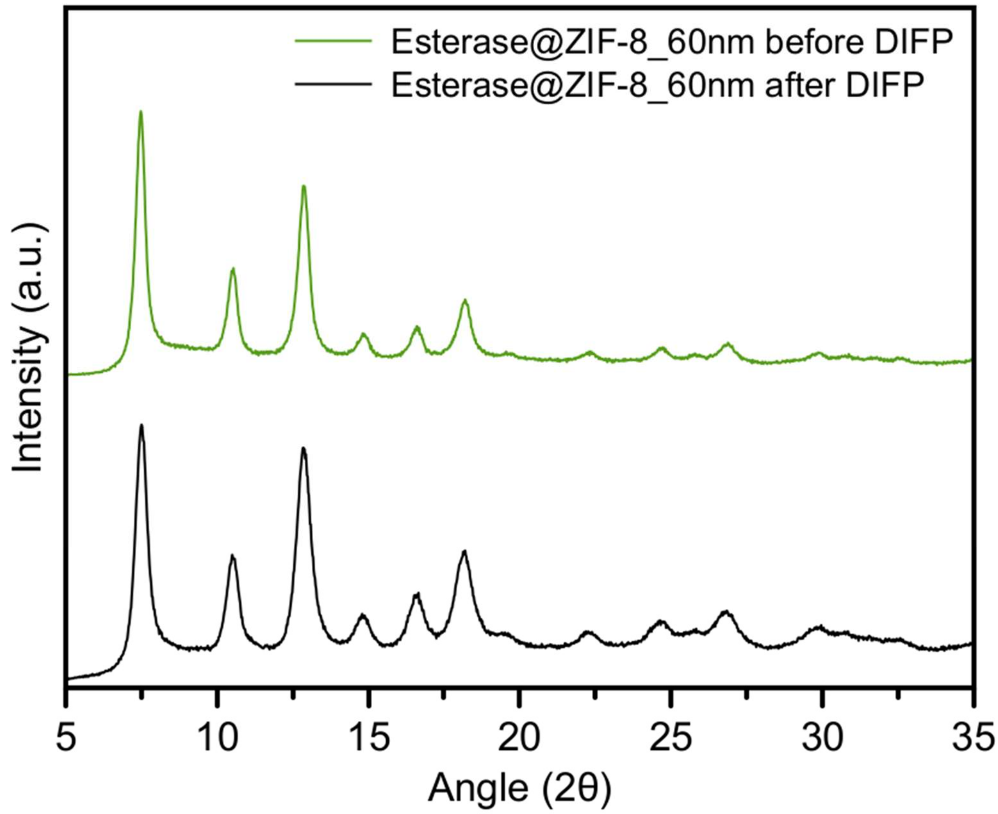

Figure S71. Powder X-Ray Diffraction patterns of Esterase@ZIF-8_60nm biocomposite before and after DIFP reaction. The comparison between each material before and after the activation protocol indicates that there is a slight widening of peaks due to particle size diminution. Experimental conditions: DIFP (0.029 M), Esterase@ZIF-8_60nm (0.084 mmol of ZIF) and Tris-HCl (0.1 M, pH = 7.4, 0.5 mL) at room temperature.

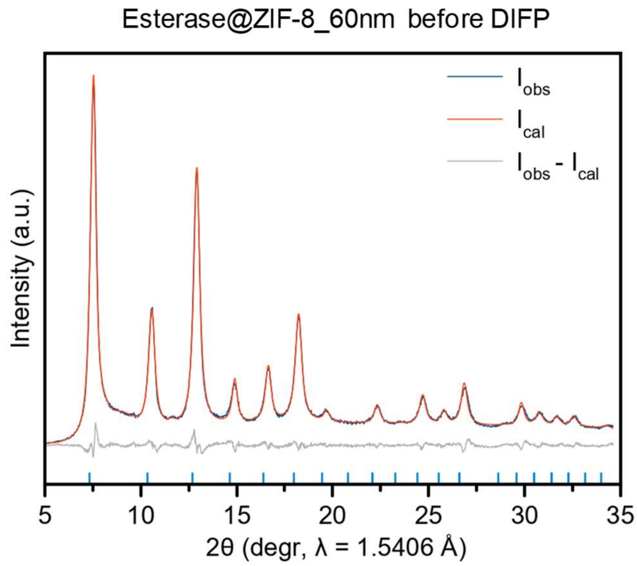

Figure S72. Graphical result of the whole powder pattern refinement carried out with the Le Bail method (R_p= 0.03647; R_{wp}= 0.04754) on the PXRD pattern of Esterase@ZIF-8_60nm before DIFP treatment in terms of observed, calculated and difference traces (blue, red and grey, respectively). The positions of the Bragg reflection are indicated by blue ticks. Cell parameters are summarized in Table S20.

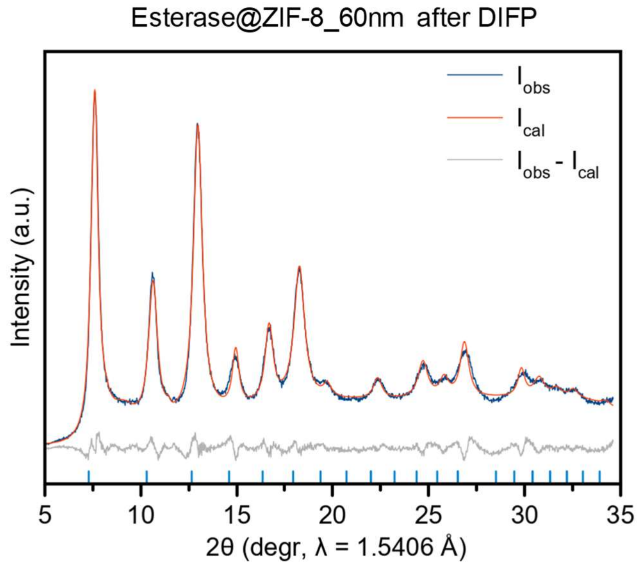

Figure S73. Graphical result of the whole powder pattern refinement carried out with the Le Bail method (R_p= 0.05633; R_{wp}= 0.07152) on the PXRD pattern of Esterase@ZIF-8_60nm after DIFP treatment in terms of observed, calculated and difference traces (blue, red and grey, respectively). The positions of the Bragg reflection are indicated by blue ticks. Cell parameters are summarized in Table S20.

Table S22. Cell parameters for Esterase@ZIF-8_60nm before and after DIFP treatment determined by PXRD Le Bail analysis. Crystalline domain size was estimated using a Lorentzian-convolution of Bragg peaks performed with the software Topas v3.

	Cell Parameter (Å)	Error (Å)	CS_L (nm)	Error_L (nm)
Esterase@ZIF-8_60nm Before DIFP	17.077	0.002	27.4	0.3
Esterase@ZIF-8_60nm After DIFP	17.129	0.004	18.2	0.3

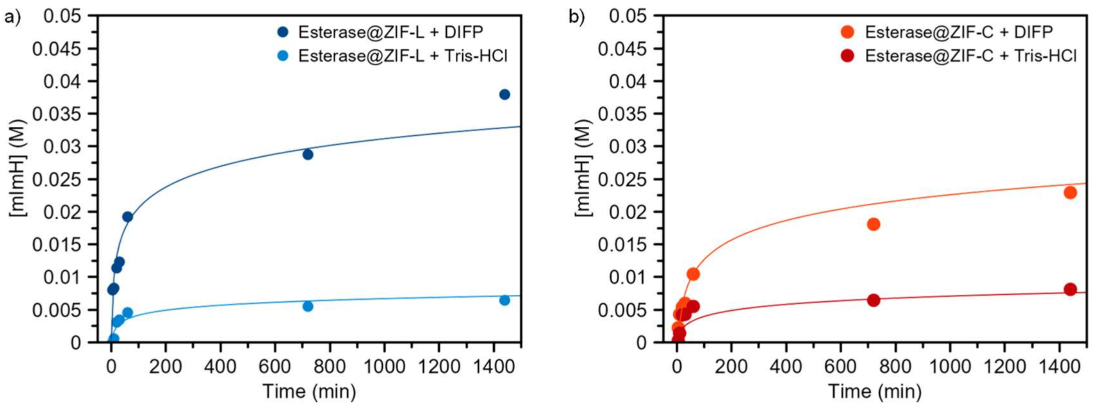

Figure S74. Comparison of 2-methylimidazole release profile for (a) Esterase@ZIF-L and (b) Esterase@ZIF-C biocomposites in the presence and absence of DIFP. Experimental points were collected by ^{1}H-NMR follow-up at different times. Experimental conditions: DIFP (0.029 M), 0.084 mmol of ZIF, dimethylacetamide (0.029 M, internal reference) and Tris-DCl (0.1 M, pD = 7.8, 0.5 mL) at room temperature.

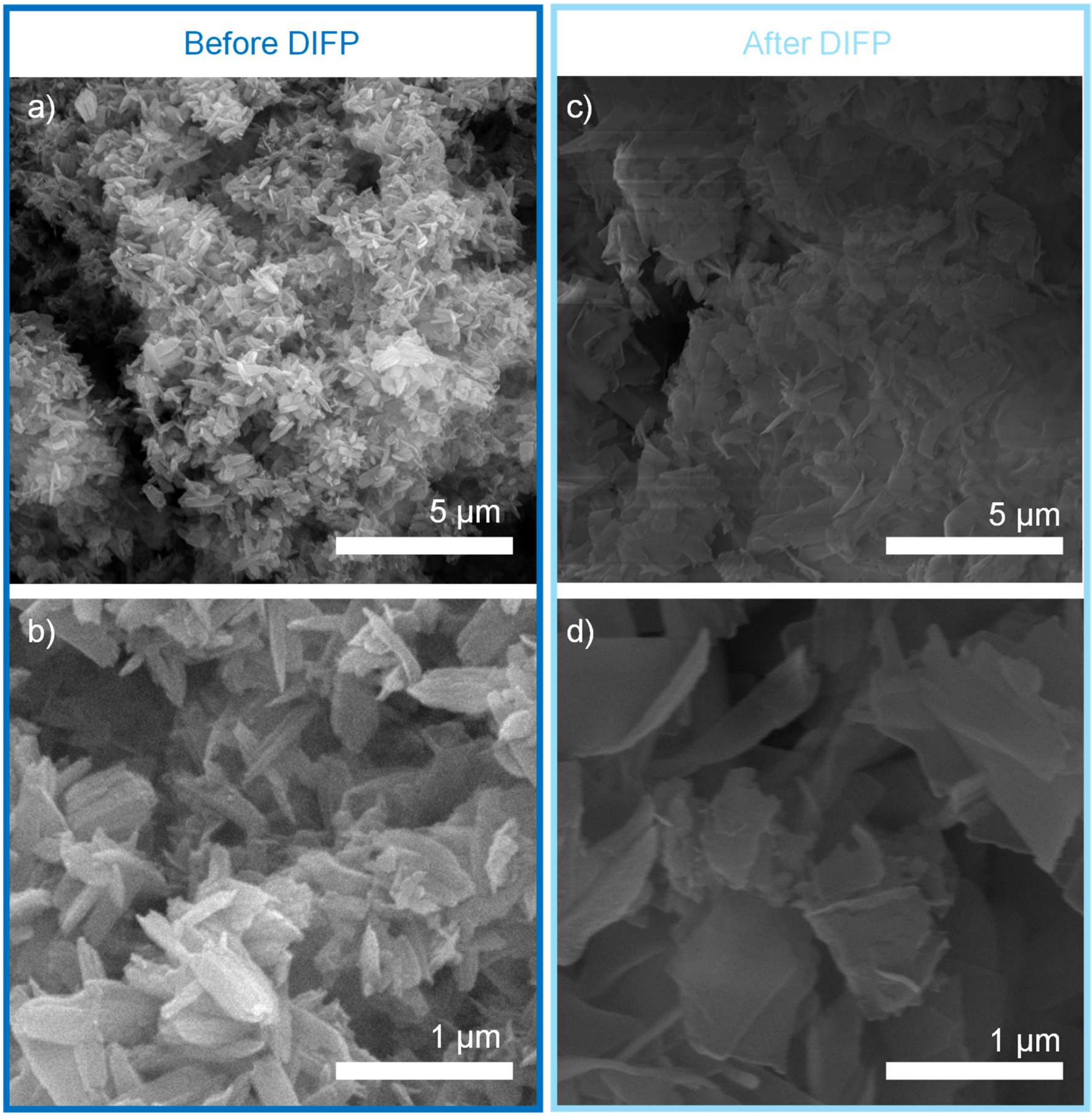

Figure S75. Scanning Electron Microscopy (SEM) images of Esterase@ZIF-L (a, b) before and (c, d) after DIFP reaction at different magnifications. Experimental conditions: DIFP (0.029 M), Esterase@ZIF-L (0.084 mmol of ZIF-L) and Tris-HCl (0.1 M, pH = 7.4, 0.5 mL) at room temperature.

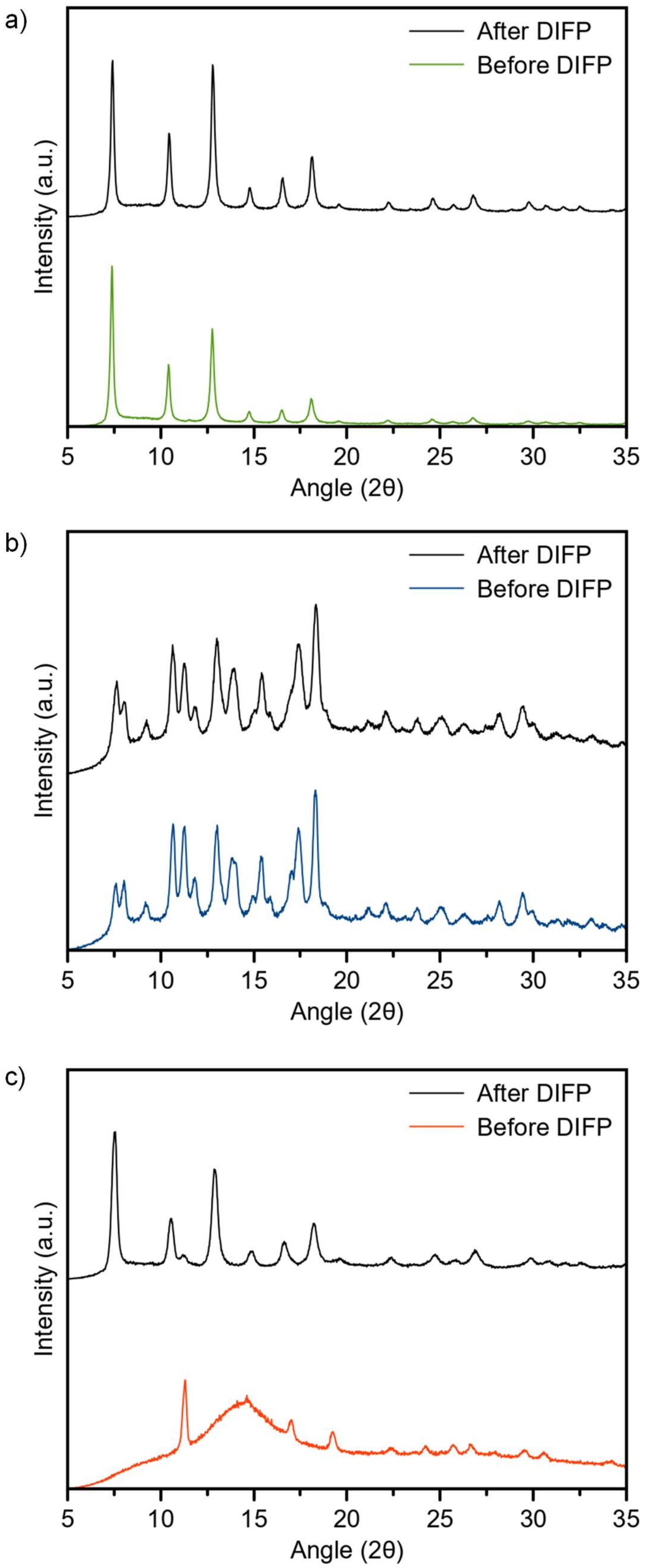

Figure S76. Powder X-Ray Diffraction patterns of (a) Esterase@ZIF-8_120nm, (b) Esterase@ZIF-L and (c) Esterase@ZIF-C biocomposites before and after DIFP reaction. Experimental conditions: DIFP (0.029 M), Esterase@ZIF (0.084 mmol of ZIF) and Tris-HCl (0.1 M, pH = 7.4, 0.5 mL) at room temperature.

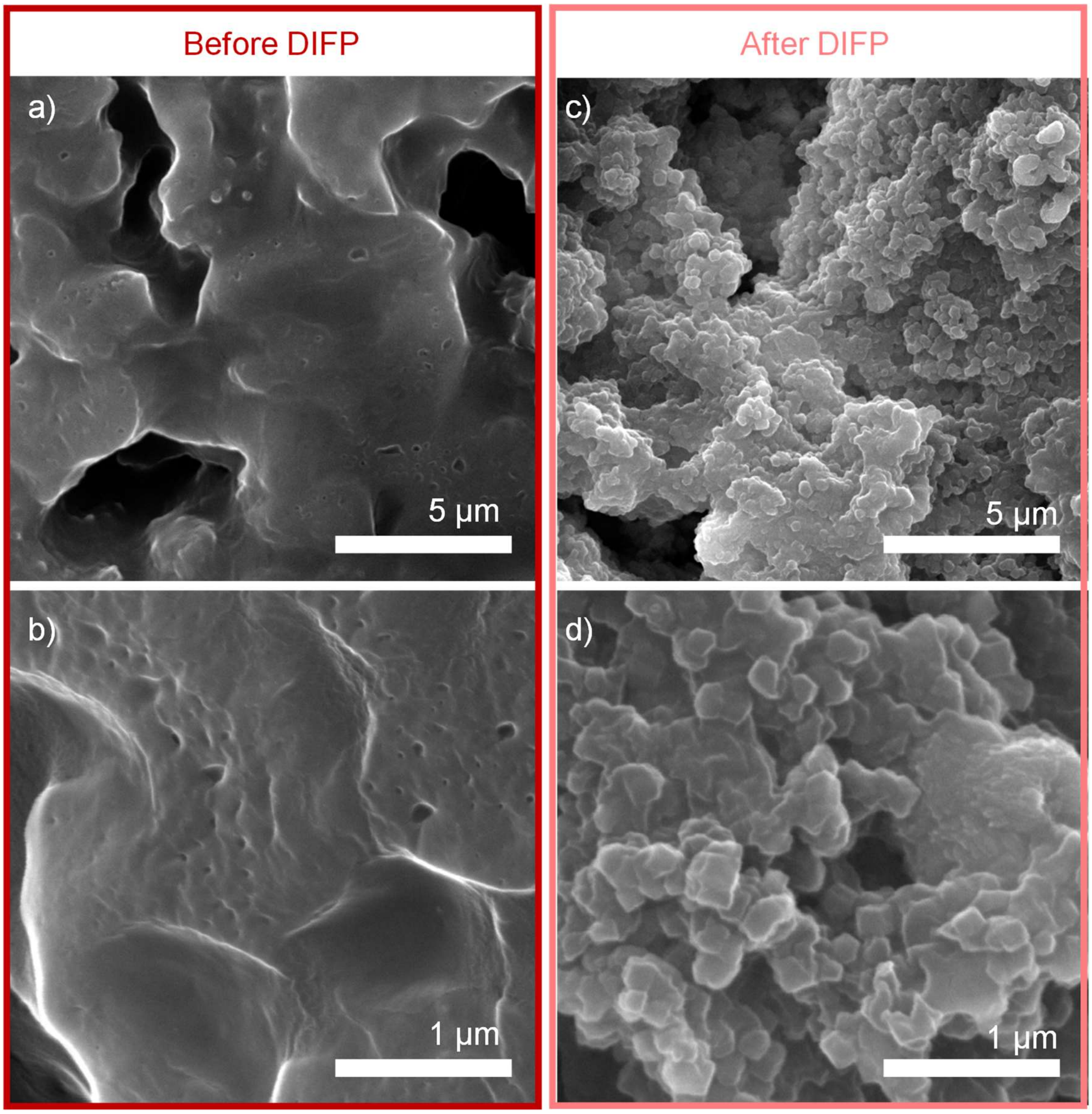

Figure S77. Scanning Electron Microscopy (SEM) images of Esterase@ZIF-C (a, b) before and (c, d) after DIFP reaction at different magnifications. Experimental conditions: DIFP (0.029 M), Esterase@ZIF-C (0.084 mmol of ZIF-C) and Tris-HCl (0.1 M, pH = 7.4, 0.5 mL) at room temperature.

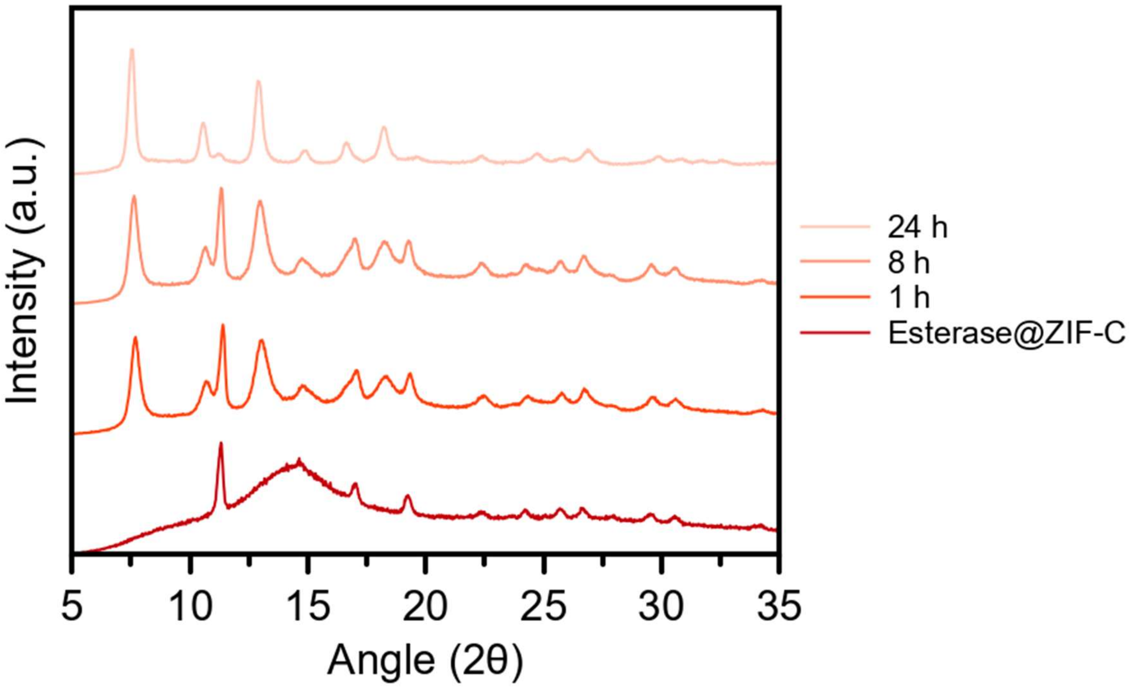

Figure S78. Powder X-Ray Diffraction patterns of Esterase@ZIF-C biocomposite along DIFP reaction at different times. We can observe a crystal phase transition from ZIF-CO_3-1 to sodalite. Experimental conditions: DIFP (0.029 M), Esterase@ZIF-C (0.084 mmol of ZIF-C) and Tris-HCl (0.1 M, pH = 7.4, 0.5 mL) at room temperature.

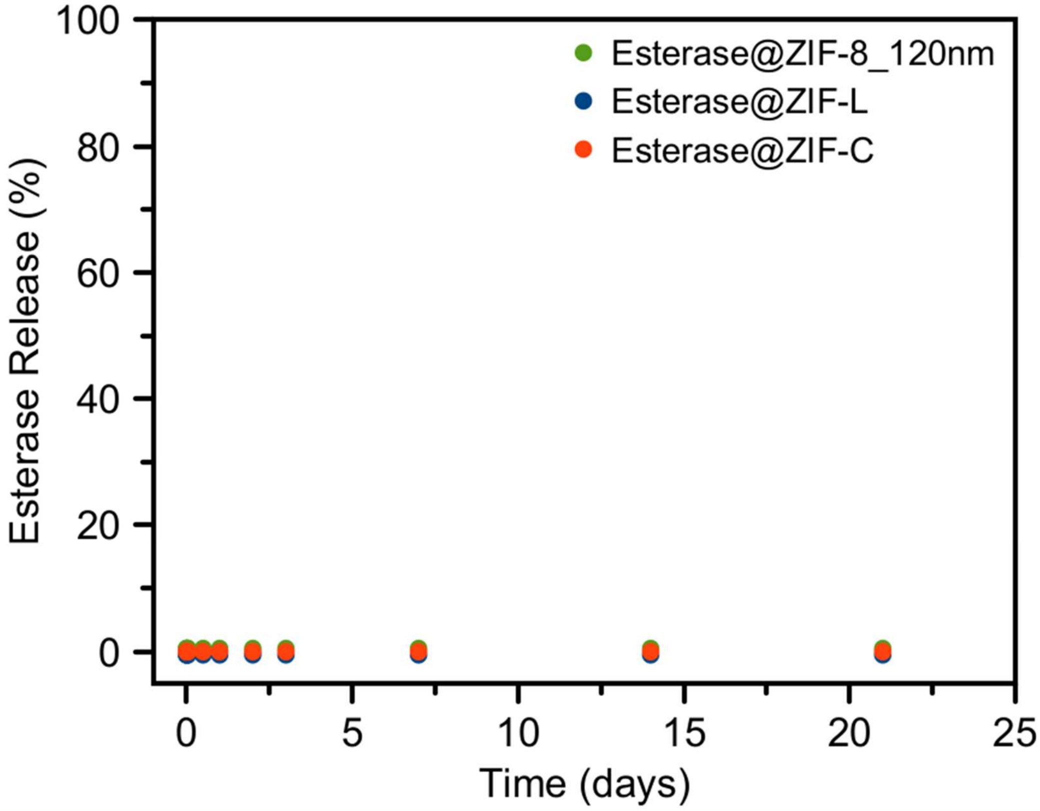

Figure S79. Esterase release profile for Esterase@ZIF-8_120nm (green), Esterase@ZIF-L (blue) and Esterase@ZIF-C (red) along the reaction of biocomposites with diisopropylfluorophosphate (DIFP) over several days. Experimental points were collected by UV-vis follow-up using the Bradford assay (see experimental protocol in S2.2.). Experimental conditions: DIFP (0.029 M), Esterase@ZIF (0.084 mmol of ZIF) and Tris-HCl (0.1 M, pH = 7.4, 0.5 mL) at room temperature.

S3.4. Detoxification ability of Esterase@ZIF biocomposites

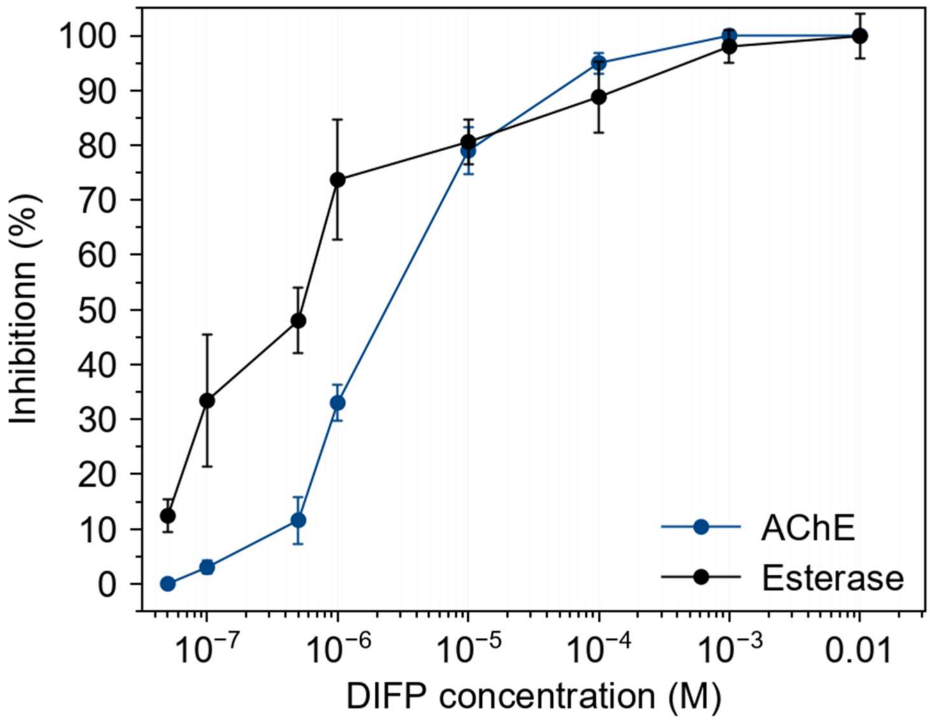

Figure S80. Esterase (black) and AChE (blue) inhibition percentage of their enzymatic activity by diisopropylfluorophosphate (DIFP) at different concentrations under simulated biological conditions (Tris-HCl buffer solution, 0.1 M, pH = 7.4). See S2.4.2. for further experimental details.

.

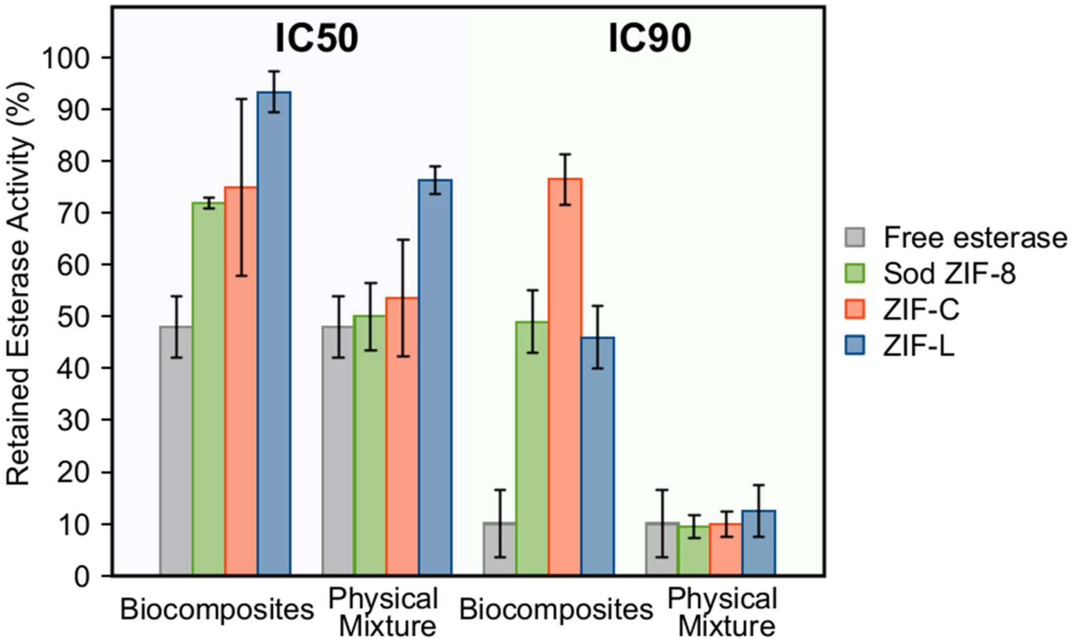

Figure S81. Retained enzymatic Esterase activity of free Esterase, Esterase@ZIFs and ZIFs+Esterase physical mixture after incubation with diisopropylfluorophosphate at Inhibitory Concentrations (IC) IC50 and IC90. The enzymatic activity of the three biocomposites was compared to each biocomposite enzymatic activity at 37 ºC. See S2.4.6. for further experimental details.

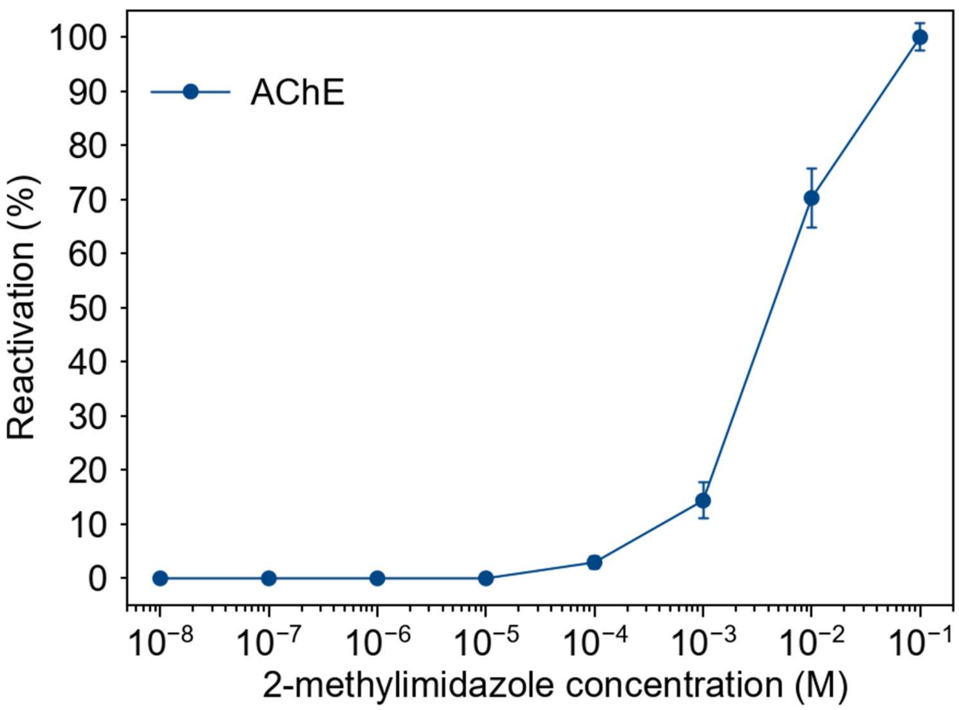

Figure S82. AChE (blue) reactivation percentage of its enzymatic activity by mImH linker at different concentrations under simulated biological conditions (Tris-HCl buffer solution, 0.1 M, pH = 7.4). See S2.4.4. for further experimental details.

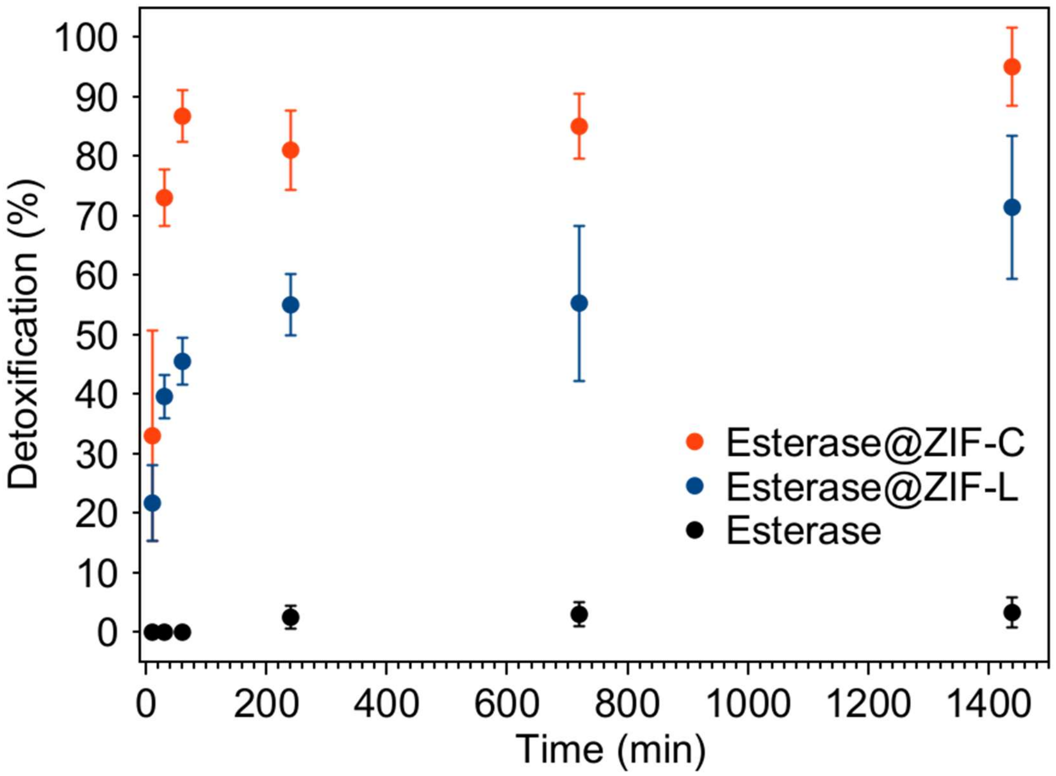

Figure S83. Detoxification profiles for Esterase@ZIF-C (red), Esterase@ZIF-L (blue) and Esterase (black) under simulated biological conditions (Tris-HCl, 0.1 M, pH = 7.4). See S2.4.7. for further experimental details.

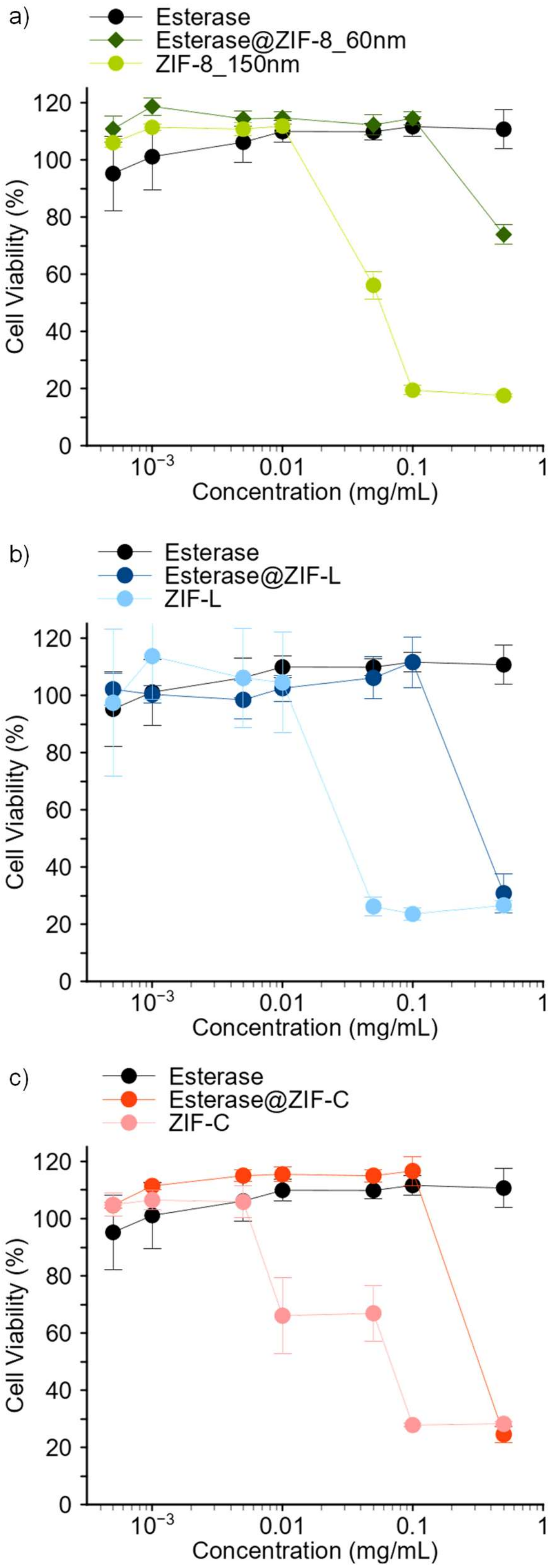

Figure S84. Cell viability of human neuroblastoma SH-SY5Y cells treated with increasing concentrations of esterase, (a) Esterase@ZIF-8_60nm/ZIF-8, (b) Esterase@ZIF-L/ZIF-L and (c) Esterase@ZIF-C/ZIF-C after 24 h of exposure.

S4. References

(1) Sánchez-Laínez, J.; Zornoza, B.; Friebe, S.; Caro, J.; Cao, S.; Sabetghadam, A.; Seoane, B.; Gascon, J.; Kapteijn, F.; Le Guillouzer, C.; Clet, G.; Daturi, M.; Téllez, C.; Coronas, J. Influence of ZIF-8 Particle Size in the Performance of Polybenzimidazole Mixed Matrix Membranes for Pre-Combustion CO2 Capture and Its Validation through Interlaboratory Test. *J Memb Sci* **2016**, *515*, 45–53. https://doi.org/10.1016/j.memsci.2016.05.039.

(2) Van Houten, J.; Barberi, R. C.; King, J.; Ogata, A. F. Improving the Colloidal Stability of Protein@ZIF-8 Nanoparticles in Biologically Relevant Buffers. *Mater Adv* **2024**, *5* (14), 5945–5957. https://doi.org/10.1039/d4ma00377b.

(3) Shu, Y.; Liang, W.; Huang, J. One-Pot Encapsulation of Enzymes in a Calcium Carboxylate Metal-Organic Framework for Improved Buffer Stability. *Advanced Science* **2025**. https://doi.org/10.1002/advs.202510960.

(4) Liang, W.; Flint, K.; Yao, Y.; Wu, J.; Wang, L.; Doonan, C.; Huang, J. Enhanced Bioactivity of Enzyme/MOF Biocomposite via Host Framework Engineering. *J Am Chem Soc* **2023**, *145* (37), 20365–20374. https://doi.org/10.1021/jacs.3c05488.

(5) Yu, T.; Ma, H.; Zhang, H.; Xiong, M.; Liu, Y.; Li, F. Fabrication and Characterization of Purified Esterase-Embedded Zeolitic Imidazolate Frameworks for the Removal and Remediation of Herbicide Pollution from Soil. *J Environ Manage* **2021**, *288*. https://doi.org/10.1016/j.jenvman.2021.112450.

(6) Greifenstein, R.; Ballweg, T.; Hashem, T.; Gottwald, E.; Achauer, D.; Kirschhöfer, F.; Nusser, M.; Brenner-Weiß, G.; Sedghamiz, E.; Wenzel, W.; Mittmann, E.; Rabe, K. S.; Niemeyer, C. M.; Franzreb, M.; Wöll, C. MOF-Hosted Enzymes for Continuous Flow Catalysis in Aqueous and Organic Solvents. *Angewandte Chemie - International Edition* **2022**, *61* (18). https://doi.org/10.1002/anie.202117144.

(7) Li, P.; Moon, S. Y.; Guelta, M. A.; Harvey, S. P.; Hupp, J. T.; Farha, O. K. Encapsulation of a Nerve Agent Detoxifying Enzyme by a Mesoporous Zirconium Metal-Organic Framework Engenders Thermal and Long-Term Stability. *J Am Chem Soc* **2016**, *138* (26), 8052–8055. https://doi.org/10.1021/jacs.6b03673.

(8) Li, P.; Moon, S. Y.; Guelta, M. A.; Lin, L.; Gómez-Gualdrón, D. A.; Snurr, R. Q.; Harvey, S. P.; Hupp, J. T.; Farha, O. K. Nanosizing a Metal-Organic Framework Enzyme Carrier for Accelerating Nerve Agent Hydrolysis. *ACS Nano* **2016**, *10* (10), 9174–9182. https://doi.org/10.1021/acsnano.6b04996.

(9) Pohanka, M.; Hrabinova, M.; Kuca, K.; Simonato, J. P. Assessment of Acetylcholinesterase Activity Using Indoxylacetate and Comparison with the Standard Ellman's Method. *Int J Mol Sci* **2011**, *12* (4), 2631–2640. https://doi.org/10.3390/ijms12042631.

(10) Barker, D. L.; Jencks, W. P. Pig Liver Esterase. Physical Properties*. *Biochemistry* **1969**, *8* (10), 3879–3889. https://doi.org/https://doi.org/10.1021/bi00838a001.

(11) Tantra, R.; Tompkins, J.; Quincey, P. Characterisation of the De-Agglomeration Effects of Bovine Serum Albumin on Nanoparticles in Aqueous Suspension. *Colloids Surf B Biointerfaces* **2010**, *75* (1), 275–281. https://doi.org/10.1016/j.colsurfb.2009.08.049.

(12) Serrano, V.; Liu, W.; Franzen, S. An Infrared Spectroscopic Study of the Conformational Transition of Elastin-like Polypeptides. *Biophys J* **2007**, *93* (7), 2429–2435. https://doi.org/10.1529/biophysj.106.100594.

(13) Kong, J.; Yu, S. Fourier Transform Infrared Spectroscopic Analysis of Protein Secondary Structures. *Acta Biochim Biophys Sin (Shanghai)* **2007**, *39* (8), 549–559. https://doi.org/10.1111/j.1745-7270.2007.00320.x.

(14) Singh, B. R. *Basic Aspects of the Technique and Applications of Infrared Spectroscopy of Peptides and Proteins*; ACS Symposium Series, 2000; Vol. 750. https://doi.org/10.1021/bk-2000-0750.ch001.

(15) Adochitei, A.; Drochioiu, G. RAPID CHARACTERIZATION OF PEPTIDE SECONDARY STRUCTURE BY FT-IR SPECTROSCOPY. *Rev. Roum. Chim* **2011**, *56* (8), 783–791.

(16) Vass, E.; Hollósi, M.; Besson, F.; Buchet, R. Vibrational Spectroscopic Detection of Beta- and Gamma-Turns in Synthetic and Natural Peptides and Proteins. *Chem Rev* **2003**, *103* (5), 1917–1954. https://doi.org/10.1021/cr000100n.

(17) GOORMAGHTIGH, E.; CABIAUX, V.; RUYSSCHAERT, J. -M. Secondary Structure and Dosage of Soluble and Membrane Proteins by Attenuated Total Reflection Fourier-transform Infrared Spectroscopy on Hydrated Films. *Eur J Biochem* **1990**, *193* (2), 409–420. https://doi.org/10.1111/j.1432-1033.1990.tb19354.x.

(18) Deacon, A.; Briquet, L.; Malankowska, M.; Massingberd-Mundy, F.; Rudić, S.; Hyde, T. I.; Cavaye, H.; Coronas, J.; Poulston, S.; Johnson, T. Understanding the ZIF-L to ZIF-8 Transformation from Fundamentals to Fully Costed Kilogram-Scale Production. *Commun Chem* **2022**, *5* (1). https://doi.org/10.1038/s42004-021-00613-z.
